# Competing gene regulatory networks drive naive and memory B cell differentiation

**DOI:** 10.1038/s44320-026-00207-8

**Published:** 2026-04-16

**Authors:** Pietro Demela, Laura Esposito, Pietro Marchesan, Leonardo Nossa, Davide Bolognini, Edoardo Giacopuzzi, Eugenia Ricciardelli, Paolo Ferrari, Silvia Bombelli, Clelia Peano, Daniele Prati, Luca Valenti, Blagoje Soskic

**Affiliations:** 1 Human Technopole, Viale Rita Levi-Montalcini 1, 20157 Milan, Italy; 2 Institute of Genetics and Biomedical Research, UoS of Milan, National Research Council, Milan, Italy; 3 Department of Transfusion Medicine, Fondazione IRCCS Ca’ Granda Ospedale Maggiore Policlinico, Milan, Italy; 4 Department of Pathophysiology and Transplantation, Università degli Studi di Milano, Milan, Italy; 5 Precision Medicine and Biological Resource Center, Fondazione IRCCS Ca’ Granda Ospedale Maggiore Policlinico Milano, Milan, Italy

**Keywords:** Computational Biology, Immunology

## Abstract

Elucidating the gene regulatory networks (GRNs) that control human B cell differentiation is crucial for understanding immune responses to infection, vaccination, and autoimmunity. Here, we map the GRNs guiding naive and memory B cell differentiation. Early in activation, both cell types engage highly similar GRNs. However, at later stages, naive B cells diverge into two opposing GRNs that promote differentiation into either plasma cells or germinal center (GC) cells. In contrast, memory B cells predominantly activate the GRNs associated with plasma cell differentiation, such as IRF4. CRISPR perturbations of IRF4 and its downstream effector PRDM1 rewired the GRNs, blocking plasma cell programs and promoting GC fate. Finally, we trained machine learning algorithms to predict sister cells, solely based on the transcriptome. This showed that plasma cell gene expression is tightly correlated among sister cells, revealing heritable transcriptional programs. Collectively, our findings reveal distinct regulatory trajectories in naive and memory B cells and uncover heritable transcriptional states that shape human B cell fate decisions.

## Synopsis

Human naive and memory B cells were shown to follow distinct differentiation trajectories governed by gene regulatory network dynamics, revealing stochastic, cell-intrinsic control of fate choice, clonal behaviour, and isotype switching.

- Integrated scRNA-seq and BCR-seq revealed that memory B cells preferentially differentiated into plasma cells, whereas naive B cells bifurcated into plasma cell and germinal centre (GC) fates.
- Temporal gene regulatory network analysis identified sustained IRF4 activity as a key driver of efficient plasma cell differentiation in memory B cells.
- IRF4 knockout redirected both naive and memory B cells toward an aberrant GC-like state, whereas PRDM1 knockout selectively blocked plasma cell differentiation while preserving GC potential.
- Clonal tracking demonstrated that individual naive B cell adopted multiple fates, with clonal identity inferable from gene expression alone.
- Isotype switching was shown to occur stochastically and cell-intrinsically, independent of clonal transcriptional state, despite strong differences in germline transcript expression between GC and plasma cells.

## Abstract (web summary)

Human naive and memory B cells were shown to follow distinct differentiation trajectories governed by gene regulatory network dynamics, revealing stochastic, cell-intrinsic control of fate choice, clonal behaviour, and isotype switching.

## Graphical abstract

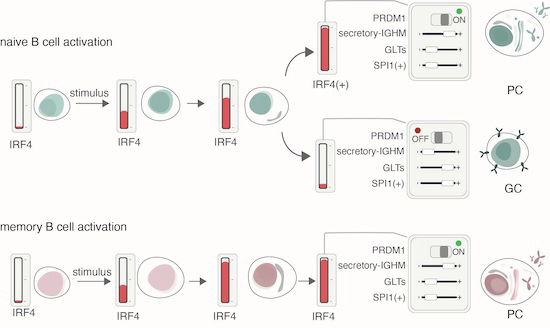

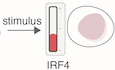

## Introduction

Upon encountering an antigen, naive B cells proliferate and their progeny can differentiate into germinal center (GC) cells, memory B cells, short-lived plasmablasts, and long-lived plasma cells, each playing a distinct role in defending the organism against pathogens (Inoue and Kurosaki 2024; Nutt et al, 2015; De Silva and Klein 2015; Victora and Nussenzweig 2022; Cyster and Allen 2019). Within the GC, B cells diversify their antibodies to respond to a wide array of pathogens. The default antibody isotype expressed by naive B cells is IgM. To enhance the diversity of the antibody repertoire, B cells undergo two key processes: somatic hypermutation (SHM) and class switch recombination (CSR) (De Silva and Klein 2015; Xu et al, 2012; Allen et al, 2007; Victora and Nussenzweig 2022). SHM improves antigen binding via point mutations in V(D)J segments of the immunoglobulin gene, while CSR replaces the constant region to alter antibody isotype. CSR generates antibodies with different effector functions (IgG, IgA or IgE) while preserving their original antigen specificity. Even though the molecular machinery responsible for CSR has been largely known (Xu et al, 2012), the causes of differential CSR outcomes are still unclear. Specifically, in a work by Horton and colleagues, CSR outcomes were explained assuming an underlying stochastic mechanism (Horton et al, 2022). In another study, intrinsic transcriptional heterogeneity of naive B cells was indicated as the leading cause of differential isotype switching outcomes (Wu et al, 2017). The dynamics and probability of antibody class switching have been largely studied in mouse models by isolating switched and unswitched cells and comparing their proportions and phenotype before and after antigen exposure. Technical advances in single-cell sequencing that allow exploration of both gene expression and antibody repertoire provide a powerful approach to capturing the transcriptomic landscapes of individual human B cells, their progeny and their antibody repertoire, allowing us to investigate transcriptional dynamics involved in this critical decision point of the immune response.

While naive B cells are critical in the primary response to antigen, memory B cells are particularly important during a secondary infection. Upon activation, memory B cells rapidly give rise to plasmablasts (Arpin et al, 1997), that increase circulating levels of high-affinity antibodies, leading to more efficient clearance of the pathogen (Inoue and Kurosaki 2024; Lam et al, 2024). The heightened responses of memory B cells could be partially attributed to the antibodies they express, which have already undergone diversification via SHM and/or CSR and exhibit high affinity for the antigen (Inoue and Kurosaki 2024; Mesin et al, 2020; Gitlin et al, 2016). This increased affinity may lower the activation threshold of memory B cells (Ambegaonkar et al, 2024; Anderson et al, 2009; Shao et al, 2024). However, experiments with naive B cells engineered to express memory-derived antibodies, showed that these cells responded similarly to wild-type naive B cells (Kometani et al, 2013). Thus, the heightened responses of memory B cells cannot be explained only by their higher antigen affinity. Finally, clonal analysis of memory B cells in mice, showed that secondary GCs are composed predominantly of clones without primary GC experience, and thus likely naive in origin (Mesin et al, 2020). As such, re-enter and new diversification of antibodies in previously diversified memory B cells is a rare event (Mesin et al, 2020; Li et al, 2024; Schiepers et al, 2024). It has been speculated that the dominance of naive-derived cells in secondary GCs may be due to precursor abundance rather than suppression of MBCs—i.e., a number of different naive B cells with high enough affinity to initiate a primary GC reaction is larger than the number of memory cells (Mesin et al, 2020; Tas et al, 2022). Another possibility is that high-affinity antibodies limit GC entry for targeted clones (Schiepers et al, 2024; Tas et al, 2022). Importantly, these studies have not investigated whether differentiation differences can also be attributed to the cell-intrinsic phenomenon of human naive and memory B cells, and what are differentiation trajectories in the absence of competition or selection.

B cell activation is mainly controlled by two opposite genetic circuits (Nutt et al, 2015; Sciammas et al, 2011; Xu et al, 2015). High levels of IRF4 induce PRDM1 expression to dictate the differentiation of B cells towards the plasma cell fate (Ochiai et al, 2013). On the contrary, when the levels of IRF4 are low, BCL6 and BACH2 steer B cell responses towards the GC fate, where B cells proliferate and diversify their antibodies. PRDM1 and BCL6 inhibit each other, and the balance between them dictates which cell state B cell will acquire (Shao et al, 2024; Sciammas et al, 2011; Ochiai et al, 2024, 2013; Kometani et al, 2013; Xu et al, 2015; Scharer et al, 2020; Nutt et al, 2015). However, it is still unclear how naive and memory B cells differently activate these opposite gene program networks and which downstream genes are in turn regulated. An in-depth understanding of how human naive and memory B cell activation is regulated over time is critical in understanding pathogen responses and, in turn, increase the effectiveness of vaccination and therapies.

To investigate the gene programs involved in B cell activation and antibody switching, we profiled the gene expression at the single cell level as well as the antibody repertoire of human naive and memory B cells at resting and following multiple time points of activation. We showed that individual naive B cells can give rise to both plasma cells and GC cells, with expression of key differentiation genes tightly regulated in a clonal manner. In contrast, memory B cells predominantly activate plasma cell-associated GRNs, characterized by progressive upregulation of IRF4 and culminating in PRDM1 expression. Through CRISPR perturbations, we defined IRF4 as a central regulator of both GC and plasma cell fates, acting independently of PRDM1 in GC commitment. Finally, leveraging machine learning classification methods trained solely on transcriptomic profiles, we accurately predicted sister cell relationships, revealing that plasma cell gene expression programs are heritable and tightly conserved within a clone. Together, these results reveal dynamic and distinct regulatory trajectories underlying naive and memory B cell differentiation, underscore the heritability of transcriptional states within clones, and advance our understanding of the complex GRNs that coordinate human B cell activation and fate determination at single-cell resolution.

## Results

### Naive and memory B cells have distinct differentiation outcomes

To investigate the activation dynamics, differentiation outcomes and control of CSR in naive and memory B cells, we established an in vitro model of B cell activation. We purified human naive and memory B cells from PBMCs (Methods). To elucidate GRNs driving isotype switching, we removed B cells expressing switched antibody isotypes. Therefore, here naive B cells are defined as CD20^+^ CD27^-^ IgG^-^ IgA^-^ and unswitched memory B cells are defined as CD20^+^ CD27^+^ IgG^-^ IgA^-^ (Appendix Fig. S1A). Next, we optimized a stimulation cocktail that models the T-dependent B cell activation (Appendix Figs. S1B,C and S2A,B). We used an IgM cross-linker providing BCR stimulation, multimeric CD40L that engages costimulatory receptor CD40 on the B cell, and cytokines IL-2 and IL-21 (Methods). Our data indicate that CD40L and IL-21 are fundamental for driving B cell proliferation, while IL-2 primarily enhances B cell survival (Appendix Fig. S2A,B). Since we isolated naive and unswitched memory B cells, we engaged the BCR with IgM cross-linker, although the strength of BCR stimulation played a lesser role in regulating B cell proliferation and survival (Appendix Fig. S2A,B).

**Figure 1:**
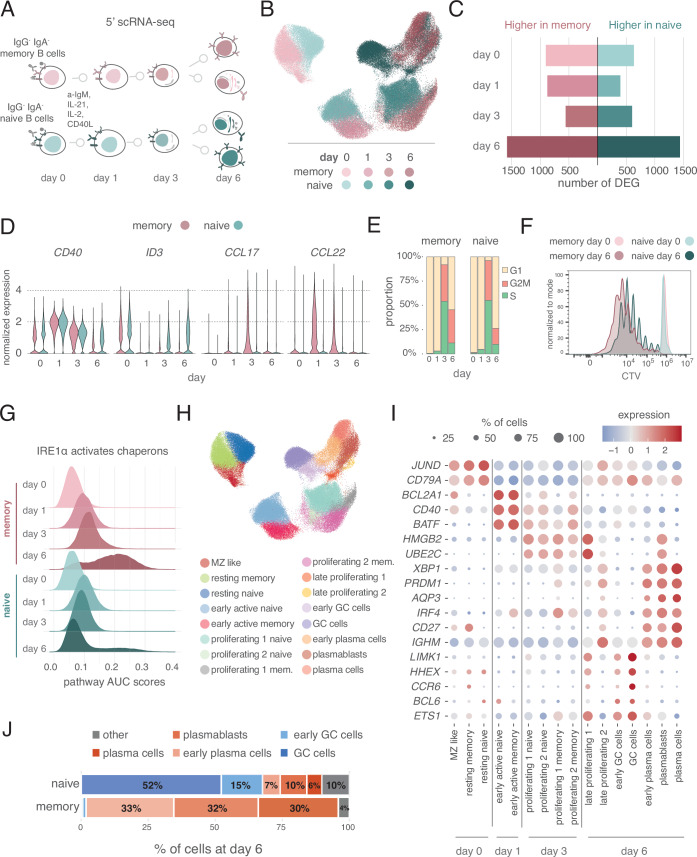
Naive and memory B cells have distinct differentiation outcomes. (**A**) Schematic overview of the study design. (**B**) UMAP embeddings of scRNA-seq of naive and memory B cells at resting and 1, 3, and 6 days following stimulation. Colors represent cell type and time point. (**C**) Barplot illustrating the number of differentially expressed genes (DEG) between naive and memory B cells at each time point. (**D**) Normalized expression level of CD40, ID3, CCL22, and CCL17. Single-cell data from *n* = 4 biological replicates. (**E**) Proportion of cells in different cell cycle phases by cell type and time point. (**F**) CellTrace violet (CTV) staining of naive and memory B cells at day six. (**G**) Density plot of AUCell scores for the “IRE1α activates chaperons” pathway stratified by cell type and time point. (**H**) UMAP embeddings of scRNA-seq of naive and memory B cells at resting and 1, 3, and 6 days following stimulation, colored by cell states. (**I**) Dotplot of cell state-specific genes. Scaled gene expression is represented on a blue-to-red gradient: blue indicates below-average expression, red indicates above-average expression. Dot size reflects the proportion of cells expressing each gene. (**J**) Proportion of cells in different cell states for naive and memory cells at day 6, colors represent cell states. (**A**–**D**, **F**, **G**) shades of purple and green represent memory and naive B cells, respectively. Source data are available online for this figure.

To comprehensively characterize activation dynamics of naive and memory B cells, we performed single-cell RNA sequencing (scRNA-seq) and B cell receptor sequencing (BCR-seq) in resting cells (day 0), before the first division (day 1), at the time of the first division (day 3) and following the full B cell differentiation (day 6) (Fig. 1A). Following quality control (Appendix Fig. S3A, Methods), we retained 118,891 cells. We used uniform manifold approximation and projection (UMAP) for dimensionality reduction and observed that cells separated by activation time point and cell type (Fig. 1B). This indicated that the transcriptional response to activation is both dynamic and cell type-specific. To further elucidate the molecular differences between naive and memory B cells, we performed differential gene expression analysis. This revealed distinct transcriptional responses following activation, with 1537, 1275, 1159, and 3017 genes significantly differentially expressed between the two cell types across days 0, 1, 3, and 6, respectively (Fig. 1C; Dataset EV1). On day 1, both naive and memory cells upregulated costimulatory receptor CD40 (Fig. 1D), indicating early activation and preparation for efficient interaction with T cells expressing CD40L. On the other hand, a proinflammatory chemokine CCL22 is highly expressed in memory B cells on day 1 and day 3 but not in naive cells (Fig. 1D). Likewise, CCL17 showed specific expression in day 3 memory B cells (Fig. 1D). CCL17 and CCL22 have been shown as critical in recruiting CCR4-expressing T cells, suggesting that memory B cells are more efficient in recruiting T cells (Liu et al, 2021). In contrast, ID3, which has been demonstrated to promote the differentiation of GC B cells and negatively regulate plasma cell differentiation (Gloury et al, 2016; Chen et al, 2016), showed markedly higher expression in activated naive cells (Fig. 1D).

Both cell types showed a progressive increase in cell cycle activity, peaking at day 3, when ~90% of cells were in S or G2M phases in both cell types (Fig. 1E). By day 6, however, ~70% of naive and 50% of memory cells had returned to the G1 phase, indicating a reduction in proliferation and increased differentiation. This observation is consistent with the higher proportion of memory B cells in later divisions (Fig. 1F) and overall higher number of memory B cells than naive B cells at day 6 (Appendix Fig. S1C).

We noted the highest number of differentially expressed genes between naive and memory B cells at day 6 (Fig. 1C; Dataset EV1). Upregulated genes in memory B were significantly enriched for pathways and signatures related to endoplasmic reticulum (ER) stress response (Fig. 1G; Dataset EV2). This included pathways such as “IRE1α activates chaperones”, “XBP1(S) activates chaperone genes”, ”Unfolded Protein Response (UPR)” and “Transport to the Golgi and subsequent modification” (Fig. 1G). As these signatures are characteristic of plasma cell differentiation (Nutt et al, 2015), we hypothesized that memory B cells are more efficient in differentiating into plasma cells than naive B cells. Therefore, to further dissect the heterogeneity of these populations, we performed unsupervised clustering of B cells and identified 16 cell states (Fig. 1H,I). Strikingly, memory B cells almost exclusively differentiated into plasmablasts and plasma cells (Fig. 1I,J). Unlike plasma cells, plasmablasts were predominantly in S or G2M phases of the cell cycle (Appendix Fig. S4A), and expressed a lower amount of IGHM antibody than plasma cells (Appendix Fig. S4B). In contrast, naive B cells had a bifurcated differentiation and generated two distinct cell states (Fig. 1J). One differentiation branch resulted in plasmablasts and plasma cells that clustered with memory counterparts, while the other resulted in a germinal center (GC) phenotype. This GC cluster was defined based on the high expression of GC markers, including BCl6, CD81, LIMK1 and high expression of multiple activation-related genes, and the absence of plasma cell markers (e.g. XBP1, IRF4, PRDM1 and low IGHM) (Fig. 1I). These cells also expressed markers of memory precursor state, such as CCR6 and HHEX (Laidlaw et al, 2020; Suan et al, 2017) suggesting that they contain the source of new memory B cell population. Differential gene expression analysis between plasma cells and GCs identified more than 8000 differentially expressed genes, suggesting a significant fate divergence. (Dataset EV3).

To further validate these lineage relationships using an independent computational approach, we applied Moscot (Klein et al, 2025), an optimal transport–based trajectory inference framework. Consistent with our observations, Moscot showed that GC cells were almost exclusively generated from proliferating naive B cells, whereas plasmablasts originated from either proliferating naive or memory B cells (Appendix Fig. S5A). These results independently confirm that, unlike memory B cells, naive B cells bifurcate into two major trajectories.

Taken together, our data demonstrate that following the same stimulation, naive and memory B cells upregulate distinct genes and pathways, resulting in significant divergence in the end cell states.

### Opposing transcription factor activities regulate fate decision

**Figure 2:**
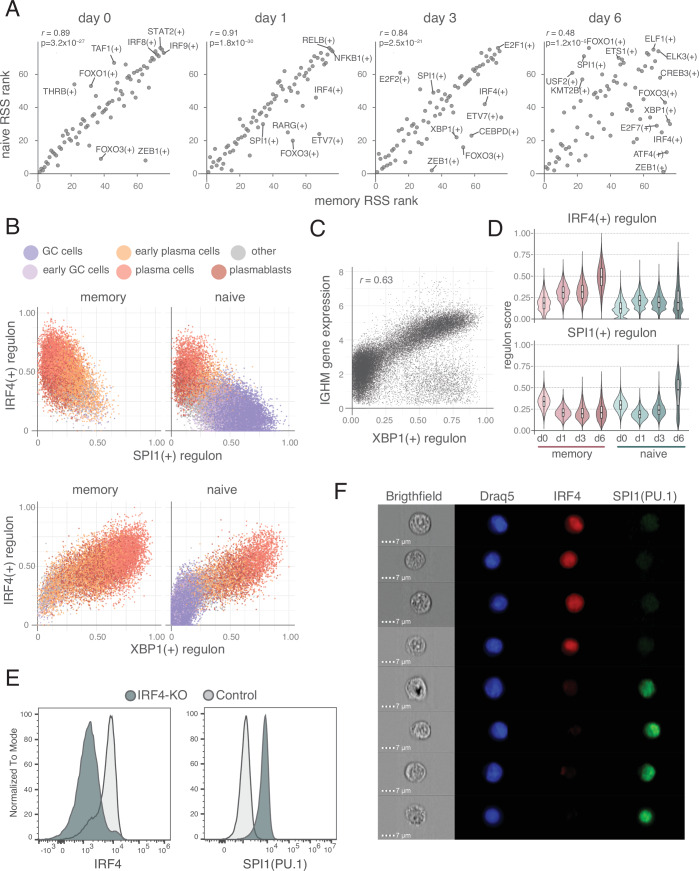
Opposing transcription factor activities regulate fate decision. (**A**) Comparison of regulon specificity score (RSS) rankings between naive and memory B cells across time points. For each condition and cell type, RSS are ranked from lowest to highest. Pearson correlation coefficient (r) and its two-sided test *P* values are shown. (**B**) Dot plots showing the SPI1, IRF4, and XBP1 regulon scores in day six naive and memory B cells. Colors represent cell states. (**C**) Dotplot showing the correlation between XBP1 regulon scores and normalized IGHM gene expression in naive and memory B cells at day 6. (**D**) IRF4 and SPI1 regulon scores across time points for naive and memory B cells. Shades of purple and green represent memory and naive B cells, respectively. Single-cell data from *n* = 4 biological replicates. In the boxplots, the center line represents the median; boxes indicate the interquartile range (25th–75th percentiles); whiskers extend to the most extreme values within 1.5× the interquartile range. (**E**) Histogram showing intracellular staining of IRF4 and PU.1 (SPI1) in IRF4 knock-out activated naive B cells. (**F**) Representative ImageStream images of activated naive B cells at day six stained for nucleus (Draq5), IRF4 and SPI1. Images were acquired at 60× magnification. Source data are available online for this figure.

Next, we hypothesized that the divergent differentiation outcomes of naive and memory B cells are driven by differences in transcription factor activity across stimulation. We reasoned that a cell’s identity is a consequence of GRNs in which a defined set of TFs regulates downstream targets. We investigated changes in the activity of transcription factors and their target genes, which are referred to as regulons. In order to infer GRN activity during differentiation of naive and memory B cells, we applied SCENIC (Aibar et al, 2017). Briefly, we identified modules of co-expressed genes and filtered them based on the presence of transcription factor-binding motifs in their promoters. At the resting state (day 0), naive and memory B cells had highly similar regulon activity for the vast majority of TFs (*r* = 0.89, *p* value = 3.2 × 10^−37^) (Fig. 2A; Appendix Fig. S6A; Dataset EV4). Among a few differences we observed, FOXO1 and FOXO3, both TFs, were previously implicated in B cell development and BCR signaling (Ottens et al, 2018; Dengler et al, 2008). Overall, the high degree of similarity of GRNs in the resting state is not surprising given that naive and memory B cells belong to the same cell type and share many core biological functions. However, TF activity became increasingly distinct between naive and memory B cells as the activation progressed, culminating on day 6 (*r* = 0.4) (Fig. 2A; Appendix Fig. S6A,B). A large number of regulons appeared to exhibit opposing activity in naive and memory cells on day 6. Notably, IRF4 regulon activity (i.e., high expression of IRF4 target genes) showed stronger activity in day 6 activated memory B cells compared to naive, while SPI1 (PU.1) regulon was most active in naive cells (Fig. 2B; Appendix Fig. S6A). These two regulons were anticorrelated and mapped onto distinct fate trajectories. While IRF4 regulon activity was strongly associated with the plasma cell fate, SPI1 activity characterized the GC state (Fig. 2B; Appendix Fig. S6B). Cells with high IRF4 activity also showed high XBP1 activity (Fig. 2B) and high antibody expression (Fig. 2C), confirming their identity as plasma cells. Moreover, we further confirmed the negative correlation between SPI1 and IRF4 by quantifying the activity of their respective regulons, using the B cell regulon annotations from (Basso et al, 2005) (Appendix Fig. S7A). Finally, we examined IRF4 and SPI1 expression along the pseudotime (Methods) and found that the level of IRF4 increases in the plasma cell trajectory, whereas SPI1 level increases in the GC trajectory (Appendix Fig. S7B,C).

Interestingly, IRF4 activity was already increased in memory B cells during early activation and continued to rise throughout the activation trajectory, consistent with the observation that memory B cells almost exclusively differentiate into plasma cells (Fig. 2D). In contrast, in naive B cells we observed a transient increase in IRF4 activity during early activation, which was subsequently downregulated and remained high only in the subset of cells that differentiated into plasma cells (Fig. 2D). Strikingly, the dynamics of SPI1 (PU.1) activity showed an inverse pattern (Fig. 2D). In memory B cells, SPI1 activity was strongly suppressed upon activation and remained low over time. However, in naive B cells, following an initial downregulation, SPI1 activity increased progressively, peaking by day 6 of activation. This opposing behavior of IRF4 and SPI1 across naive and memory cells led us to hypothesize that IRF4 activity may inhibit SPI1 activity. To test this relationship, we used CRISPR-Cas9 to knock out IRF4 in activated naive B cells. IRF4 deletion resulted in an increase in SPI1 (PU.1) expression, supporting the hypothesis that IRF4 negatively regulates SPI1 (Fig. 2E). Finally, we performed imaging flow cytometry to assess the subcellular localization of IRF4 and SPI1 (PU.1). This analysis revealed a clear and mutually exclusive pattern. B cells that have IRF4 localized in the nucleus were negative for SPI1 (PU.1), and vice versa (Fig. 2F; Appendix Fig. S8A–C).

Taken together, these findings demonstrate that transcription factor regulatory networks diverge significantly between naive and memory cells during activation. By day 6, opposing GRNs are active, reflecting the underlying commitment to either plasma cell or GC fates.

### Plasma cells and GC cells can arise from the activation of the same progenitor cell

**Figure 3:**
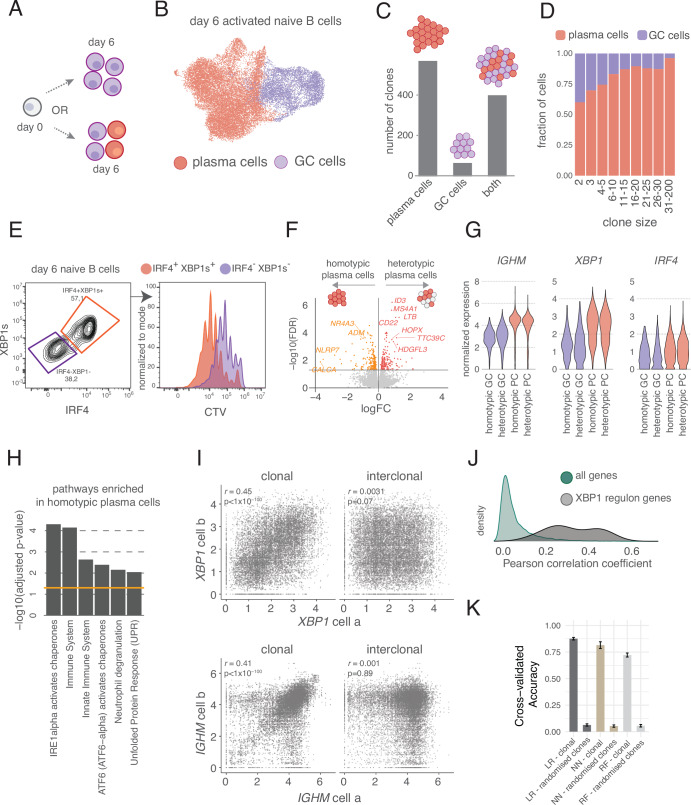
Plasma cells and GC cells can arise from the activation of the same progenitor cell. (**A**) Schematic overview of two hypotheses. (**B**) UMAP embeddings of scRNA-seq of naive B cells 6 days following stimulation. Colors represent cell state. (**C**) Barplot of the number of clones giving rise to only plasma cells, only GC cells or both. (**D**) Proportion of different cell states per clonal size. Colors represent cell state. (**E**) The left plot shows intracellular staining of IRF4 and XBP1 in naive B cells at day 6 after activation. The right plot shows CellTrace violet (CTV) proliferation analysis of the subpopulations on the left. (**F**) Volcano plot of differential gene expression between homotypic and heterotypic plasma cells. Gray indicates non-differentially expressed genes; orange and red indicate genes up- and downregulated in heterotypic plasma cells, respectively. *P* values were calculated with the glmtreat test in edgeR, and multiple test correction was applied with the BH method. Single-cell data from *n* = 2 biological replicates of homotypic and heterotypic plasma cell clones. (**G**) Normalized expression level of IGHM, XBP1, and IRF4, separated by clone type. Colors represent cell state. Single-cell data from *n* = 2 biological replicates. (**H**) Top enriched reactome pathways in homotypic plasma cells. *P* values were calculated with the hypergeometric test and corrected for multiple testing with the gprofiler-g:SCS. (**I**) Interclonal and clonal correlation of XBP1 and IGHM normalized gene expression. Pearson correlation coefficient (*r*) and its two-sided test *P* values are shown. (**J**) Density plot of clonal Pearson correlation coefficient for XBP1 regulon genes compared to all other genes. (**K**) Classification accuracy (%) of three machine learning algorithm models, used to predict clonal identity based on gene expression profiles. Each bar represents the mean and standard error of the performance metric across cross-validation folds (K = 5). Logistic regression (LR), neural network (NN), random forest (RF). Accuracy is calculated as the proportion of correctly assigned cells to clones to total cells. (**A**–**D**, **G**) Orange and purple represent plasma cells and GC cells, respectively. Source data are available online for this figure.

Given that naive B cells differentiate into plasma cells and GC cells, we next sought to identify whether this bifurcation arises from a population-level behavior (i.e., different cells commit to different fates), or whether an individual cell is capable of generating both fates (Fig. 3A). We reasoned that if one cell gives rise to both cell states at day 6, then as that cell divides and forms a clone, we should observe both cell states within that clone. To address this, we developed an improved B-cell stimulation protocol that allowed us to expand a smaller number of naive B cells in culture (2000 B cells at day 0). On day 6, we performed single-cell RNA- and BCR-sequencing on the entire culture, preserving both clonal diversity and size. We sequenced 23,484 cells, and identified 265 clones with at least ten cells (Fig. 3B). Strikingly, this revealed that a large proportion of the clones contained both plasma cell and GC cell states while others produced only plasma or GC cells (Fig. 3C). Interestingly, we also noted that clones enriched in plasma cell fraction tended to be larger (Fig. 3D). To validate this observation, we stained for XBP1 and IRF4 as proxies for plasma cell fate, and showed that XBP1^+^IRF4^+^ cells proliferate more extensively than XBP^-^ IRF4^−^ (Fig. 3E; Appendix Fig. S9A).

We next assessed whether the plasma cells from “plasma cell only” clones were different from plasma cells in mixed clones (plasma cell+GC cells) (Fig. 3F). We referred to these populations as homotypic and heterotypic plasma cells, respectively. Importantly, there was no difference in key plasma cell genes IGHM, XBP1 and IRF4, further confirming their plasma cell identity (Fig. 3G). However, 412 genes were differentially expressed between homotypic and heterotypic plasma cells. Genes upregulated in homotypic plasma cells were enriched in ER stress response pathways, including “IRE1alpha activates chaperones”, “ATF6 (ATF6−alpha) activates chaperones” and “Unfolded Protein Response (UPR)” (Fig. 3H). This demonstrated that plasma cells from homotypic clones have stronger ER stress signatures.

Next, we hypothesized that if heterotypic and homotypic plasma cells have distinct transcriptional profiles, each clone could carry its own unique gene expression signature. We reasoned that if the level of gene expression is heritable across cell divisions, then sister cells should display similar mRNA expression profiles. To test this, we analyzed the gene expression correlation between sister cells and compared them to correlations observed in random cell pairs (Appendix Fig. S9B). Strikingly, many top correlated genes were involved in plasma cell differentiation and function. For example, we observed that XBP1 and IGHM showed high gene expression correlation within a clone (*r* = 0,45 and *r* = 0.41, respectively) (Fig. 3I). In contrast, when we analysed random B cell pairs from the same donor, there was no significant correlation (Fig. 3I). This indicated that the level of antibody expression is similar between sister cells. Given that XBP1 is a transcription factor, we reasoned that if its expression is clonally correlated, then the genes it regulates should also show similar correlation patterns. To test this, we examined the expression correlation of XBP1 regulon genes among sister cells. We found that these genes displayed higher correlation between sister cells than all other genes (Fig. 3J; Appendix Fig. S9C), indicating that genes critical to the plasma cell lineage tend to be clonally inherited in their expression profiles. Finally, we asked whether these intraclonal gene correlations could lead to clone-specific gene expression signatures. To test this, we trained three machine learning classifiers, a logistic regression (LR), neural network (NN), and random forest classifier (RF) (Methods) to predict sister cells based on gene expression patterns and validated it against VDJ-assigned clonal identities. Remarkably, using genes whose clonal correlation coefficient is above 0.25 Dataset EV5), we were able to correctly predict sister cells for 80% of the cells on average (LR accuracy 0.876 ± 0.012; NN accuracy 0.828 ± 0.019, RF accuracy 0.722 ± 0.019 (Fig. 3K; Appendix Fig. S10A,B). We showed that random sets of genes whose clonal correlation was lower than 0.25 were not informative enough, and all classifiers perform poorly when using these sets of genes (average of 100 random sets of genes with *r* < 0.25; LR accuracy 0.276 ± 0.031; NN accuracy 0.274 ± 0.030, RF accuracy 0.246 ± 0.029) (Appendix Fig. S10C). Furthermore, classification performances were unaltered when cell cycle and cell state effects were regressed out (Appendix Fig. S10B), indicating that the models were able to learn clonal-specific signatures that are independent from cell cycle and cell state-specific programs. Finally, we randomly shuffled clonal assignments within each donor 1000 times to generate a distribution of prediction accuracy as expected by chance, demonstrating that the classifiers did not learn any meaningful patterns in this scenario (Appendix Fig. S11A).

To validate that this is not an in vitro artefact, we conducted classification on 11 independent in vivo mice B cell clonal datasets (Agrafiotis et al, 2023), using the logistic regression (LR) and the neural network (NN) models. For 7 out of 11 mice datasets, classification accuracy was above 70% with both LR and NN, while for all samples accuracy was above 50% (NN accuracy across all mice 0.72 ± 0.098; LR accuracy across all mice 0.71 ± 0.10) (Appendix Figs. S12A and S13A). This indicated that the expression level of a multitude of genes involved in B cell function are highly heritable across divisions, resulting in sister cells with similar gene expression patterns.

Taken together, we demonstrated that one naive B cell has the potential to generate both plasma cells and GC cells, and that gene expression levels of genes involved in B cell differentiation are highly clonally regulated.

### Class-switching is clonally independent

**Figure 4:**
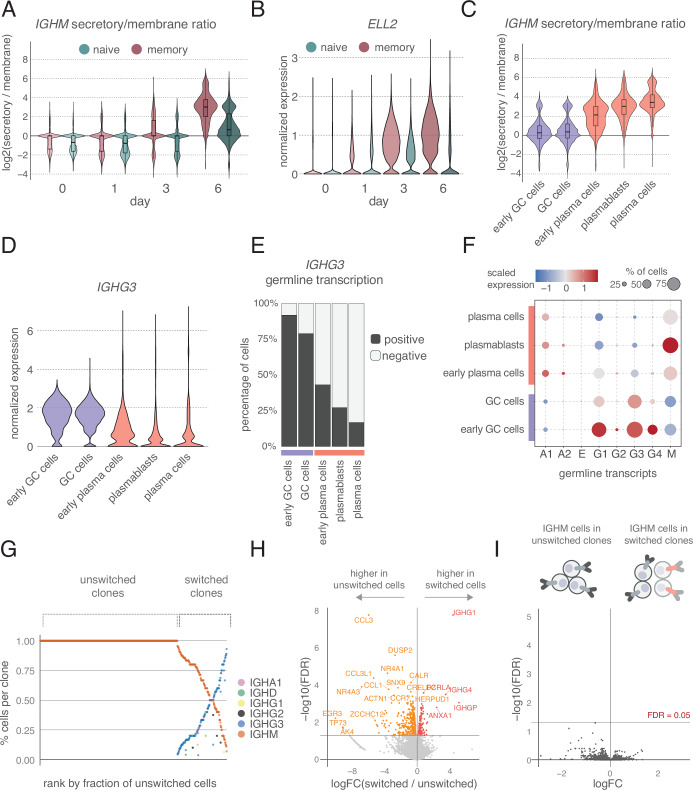
Class-switching is clonally independent. (**A**) Ratio of IGHM secretory to IGHM membrane mRNA isoforms in naive and memory B cells per time point. Single-cell data from *n* = 4 biological replicates. In the boxplots, the center line represents the median; boxes indicate the interquartile range (25th–75th percentiles); whiskers extend to the most extreme values within 1.5× the interquartile range. (**B**) Normalized expression level of ELL2 in naive and memory B cells per time point. Single-cell data from *n* = 4 biological replicates. (**C**) Ratio of IGHM secretory to IGHM membrane mRNA isoforms in GC and plasma cells. In the boxplots, the center line represents the median; boxes indicate the interquartile range (25th–75th percentiles); whiskers extend to the most extreme values within 1.5× the interquartile range. Single-cell data from *n* = 4 biological replicates. (**D**) Normalized expression level of IGHG3 in GC and plasma cells. Single-cell data from *n* = 4 biological replicates. (**E**) Proportion of cells expressing sterile and productive IGHG3 transcripts. (**F**) Dotplot of GLT expression in GC and plasma cells. Scaled gene expression is represented on a blue-to-red gradient: blue indicates below-average expression, red indicates above-average expression. Dot size reflects the proportion of cells expressing each GLT. (**G**) Isotype fractions per clone. Clones are ranked by the fraction of IGHM^+^ cells. Colors represent isotypes. (**H**) Volcano plot of differential expression between unswitched and switched cells. Gray represents non-differentially expressed genes, orange and red represent upregulated and downregulated genes in switched cells, respectively. *P* values were calculated with the glmtreat test in edgeR and multiple test correction was applied with the BH method. Single-cell data from *n* = 4 biological replicates. (**I**) Volcano plot of differential expression between IGHM^+^ cells from unswitched and switched clones. Single-cell data from *n* = 4 biological replicates.

Next, we investigated how naive and memory B cells regulate the production of antibodies. As the IgM isotype is most common in our culture (i.e., 90% of cells are IgM^+^, Appendix Fig. S14A), specifically, we quantified the ratio of secretory (sIGHM) versus membrane (mIGHM) transcript isoforms across the time course of activation. As expected, at resting (day 0) and at early activation time point (day 1), both naive and memory B cells predominantly expressed the mIGHM (Fig. 4A). However, by day 3, the isoform ratio had already shifted in favor of the sIGHM in memory B cells and not in naive. This trend continued by day 6, when nearly all memory cells expressed sIGHM at substantially higher levels than naive cells. In contrast, naive cells retained a more balanced IGHM isoform ratio. This difference in isoform ratio dynamics between naive and memory B cells is likely explained by the expression dynamics of ELL2 (Fig. 4B), known to promote the use of the secretory-specific polyadenylation site in the IGHM gene (Martincic et al, 2009). Notably, ELL2 expression is regulated by IRF4 (Dataset EV4), which is exclusively active in plasma cells (Fig. 2B), consistent with our observation that the secretory isoform was detected in plasma cells but not in GC cells at day 6 (Fig. 4C).

Next, we explored isotype switching and found that a large proportion of GC cells expressed IGHG3 (Fig. 4D), which was unexpected given that only 15% of day 6 activated naive cells switched antibody class (Appendix Fig. S14A). To investigate this discrepancy, we re-analysed the data by remapping reads using the sciCSR pipeline (Ng et al, 2023), enabling more accurate detection and quantification of antibody isotypes. Isotype switching requires the expression of short transcripts that are transcribed upstream of the transcription start site of the first exon of every isotype, known as germline transcripts (GLTs) (Chaudhuri et al, 2003; Xu et al, 2012; Lorenz et al, 1995; Chowdhury et al, 2008). This transcript does not result in a functional antibody protein, but it is required in the recruitment of AID during the class-switching process, thus marking the switching regions poised for CSR. Strikingly, above 75% of GC cells expressed germline IGHG3 while only 25% of plasma cells showed this pattern (Fig. 4E). This priming, however, did not lead to an increase in the proportion of switched cells in GC (Appendix Fig. S14B), as GLTs are necessary but not sufficient to lead to antibody class switching. We observed a similar pattern for IGHG1 and to a lesser extent for IGHG4 (Fig. 4F). Therefore, together this demonstrated that germline transcription in the IGH antibody locus is significantly more active in GC cells than in plasma cells, suggesting a cell-state specific transcription activity in the IGH locus.

To investigate potential transcriptional differences between switched and unswitched B cells, we focused on characterizing cells that had undergone CSR during in vitro stimulation. Although switched cells were excluded during the initial sorting of naive and memory populations, some may still be present due to low-level contamination. To confidently distinguish between contamination and CSR upon activation, we leveraged clonal information and included only clones containing at least one IGHM-expressing cell. Since CSR is one-directional and irreversible, the presence of IGHM within a clone ensured that any observed isotypes arose from activation rather than from pre-existing switched cells in the starting population. Using this approach, we identified 764 clones containing only unswitched cells and 258 clones containing switched cells in varying proportions (Fig. 4G). We then performed differential gene expression analysis and detected 196 upregulated and 377 downregulated genes in switched cells (Fig. 4H; Dataset EV6), indicating that CSR is associated with distinct transcriptional programs. Interestingly, we observed higher ER-stress related signatures, including “Protein processing in endoplasmic reticulum” in unswitched cells (Appendix Fig. S14C; Dataset EV7) which may be explained by the fact that due to the size and complexity of multimeric IgM, its protein folding is more complex than that of IgG. Finally, a mathematical model of isotype switching demonstrated recently that CSR is independent of clonal membership (Horton et al, 2022), suggesting an underlying stochastic process. This would, in turn, imply that there is no transcriptional determinant of switching, and the probability of switching of a single IgM^+^ cell is independent of its sister cell. To test this further, we investigated the transcriptional difference between IgM^+^ cells from clones containing switched cells, and IgM^+^ cells from unswitched clones. We observed no transcriptional differences between them (Fig. 4I), demonstrating that IgM^+^ cells from switched clones are not different from IgM^+^ cells from unswitched clones. This implies that belonging to the same clonal ancestry as a switched cell, does not increase the likelihood of further switching, validating the proposed mathematical model.

### PRDM1 and IRF4 have different roles in fate bifurcation

**Figure 5:**
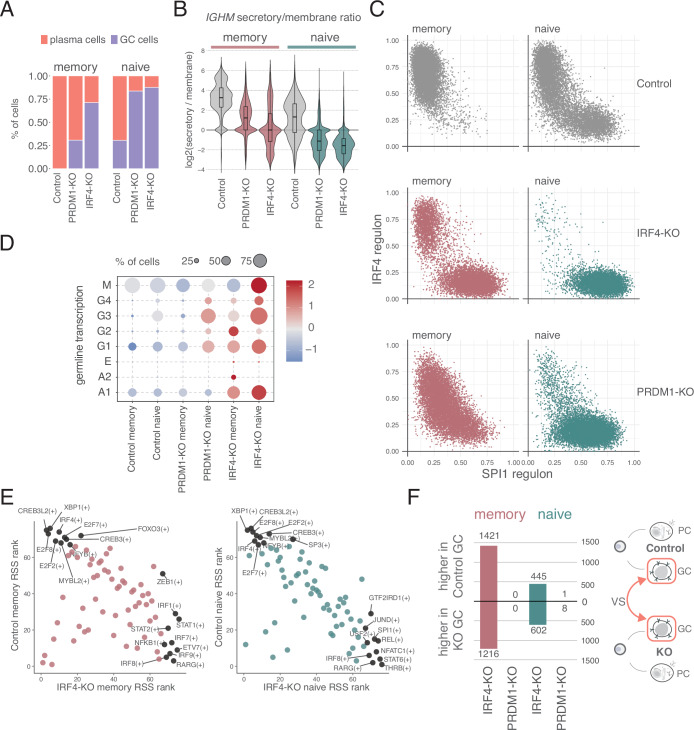
PRDM1 and IRF4 have different roles in fate bifurcation. (**A**) Proportion of cells in different cell states for naive and memory cells at day 6. Purple and orange represent GC cells and plasma cells, respectively. (**B**) Ratio of IGHM secretory to IGHM membrane mRNA isoforms in naive and memory B cells per condition. In the boxplots, the center line represents the median; boxes indicate the interquartile range (25th–75th percentiles); whiskers extend to the most extreme values within 1.5× the interquartile range. Single-cell data from *n* = 2 biological replicates. (**C**) Dotplot showing the SPI1 and IRF4 regulon scores in naive and memory B cells at day 6. (**D**) Dotplot of germline transcription expression in day 6 naive and memory cell knock-out and control conditions. Scaled gene expression is represented on a blue-to-red gradient: blue indicates below-average expression, red indicates above-average expression. Dot size reflects the proportion of cells expressing each GLT. (**E**) Comparison of RSS ranks between IRF4-KO and control conditions in naive and memory B cells. For each condition and cell type, RSS are ranked from lowest to highest. Pearson correlation coefficient (*r*) and its two-sided test *P* values are shown. Top ten highly ranked regulons per condition are highlighted in black. (**F**) Number of differentially expressed genes between naive control GC cells and all other conditions.

Since we observed that IRF4 activity has distinct dynamics during naive and memory B cell differentiation trajectory, we next investigated how it influences cell fate decisions and antibody production in both cell types. IRF4 is a well-known regulator of transcription factor PRDM1, driving plasma cell differentiation. To examine their role in both naive and memory B cell differentiation, we knocked out IRF4 and PRDM1 (Methods). By performing PCR and Sanger sequencing, we confirmed the knock-out for both IRF4 and PRDM1 with an average efficiency of 98% for IRF4 and 90% for PRDM1 (Appendix Fig. S15A) (Methods). This resulted in a marked reduction in the frequency of plasma cells across both cell types, though the effect of IRF4 KO appeared to be stronger (Fig. 5A).

We further confirmed the reduction of plasma cells in the knock-out by applying scANVI (Xu et al, 2021), which leverages labeled data from the time course to learn a latent-space cell-type classifier and subsequently predict cell types in the KO dataset. This analysis confirmed that the remaining B cells correspond to GC and early GC B cells (Appendix Fig. S16A,B). Finally, to confirm that IRF4 and PRDM1 impairment induced a rewiring of B cells gene regulatory network, we performed an in silico knock out of IRF4 and PRDM1 to simulate cell state transitions with CellOracle (Kamimoto et al, 2023). These simulations predicted that IRF4 and PRDM1 loss would suppress plasma cell differentiation, that SPI1 loss would impair GC fate acquisition (Appendix Fig. S17A,D). Our experimental KO data are in line with these predictions, exhibiting the expected shifts in cell identity.

This was accompanied by a significant decrease in the secretory to membrane IGHM isoform ratio (Fig. 5B). As expected the cells that were KO for IRF4 exhibited significant reduction in IRF4 activity, evident by decrease in IRF4 regulon scores (Fig. 5C). Strikingly, instead they all showed increased activity of the SPI1 (PU1) regulon, which has not been observed in control memory cells (Fig. 5C). A similar effect was observed following PRDM1 knockout, though the effect on memory was less pronounced than on naive B cells (Fig. 5C). Finally, we observed a strong increase in germline transcription of IgH genes with the particular increase in germline IGHG1, IGHG2, IGHG3, and IGHA1 (Fig. 5D).

In addition, we noted substantial transcriptional rewiring following IRF4 and PRDM1 KOs, with over 5000 and 2000 genes differentially expressed compared to control, respectively (Dataset EV8). However, both IRF4 and PRDM1 KOs caused a shift in GRN activity compared to control, as indicated by large deviations in regulon specificity scores from the diagonal (Fig. 5E). For instance, the XBP1 regulon which is highly active in control condition was completely inactive following IRF4 and PRDM1 KO (Fig. 5E). Next, we investigated whether the emergence of SPI1-regulon high cells in activated memory B cells following IRF4 and PRDM1 KOs resembled the GC cells arising from natural naive B cell differentiation. To investigate this, we compared GC cells from control naive cells to those arising in memory cells following IRF4 and PRDM1 KOs. Strikingly, GC cells from memory B cells lacking PRDM1 were transcriptionally indistinguishable from those arising from naive cells in the control condition (Fig. 5F). This demonstrated that PRDM1 is only essential for plasma cell generation but dispensable for GC cell development. In contrast, GC cells emerging in memory cells following IRF4 KO displayed over 2637 differentially expressed genes compared to naturally arising GC cells following activation of naive B cells (Dataset EV9). Despite expressing canonical GC markers (Appendix Fig. S18A), these cells were transcriptionally highly distinct, indicating that IRF4 plays a role in both plasma cell and GC differentiation (Fig. 5F). Similarly, following IRF4 KO in naive cells, we observed a reduction in the proportion of plasma cells and an increase in aberrant GC cells. This suggests that while the plasma cell differentiation was blocked, GC differentiation was impaired, resulting in an aberrant GC state.

**Figure 6:**
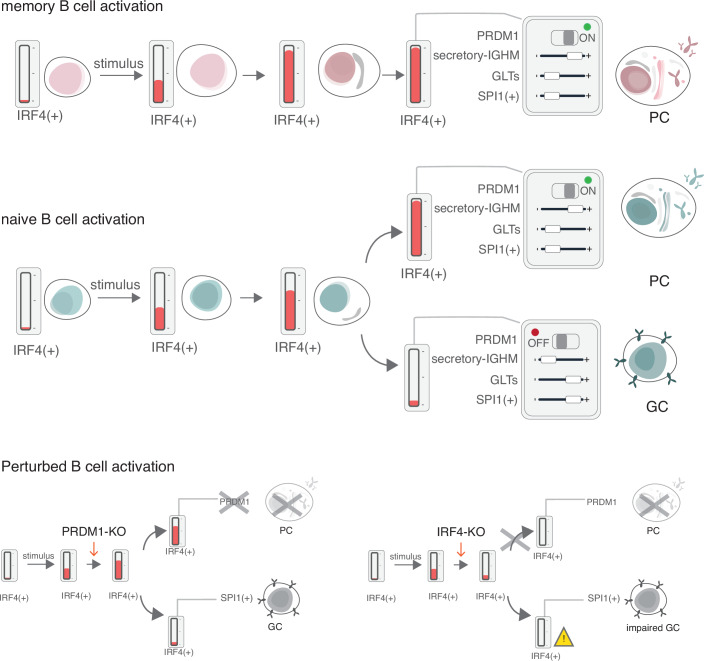
Model of IRF4 control of naive and memory B cell differentiation. Top: Memory B cell activation is characterised by sustained IRF4 upregulation following stimulation, promoting PRDM1 expression while inhibiting SPI1 activity, ultimately driving differentiation into plasma cells (PCs) and antibody secretion. Middle: Naive B cells exhibit graded IRF4 induction after stimulation, leading to bifurcation of cell fate. High IRF4 levels favor PRDM1 activation and plasma cell differentiation, whereas lower IRF4 levels maintain SPI1 activity and support germinal center (GC) cell differentiation. This process is accompanied by induction of germline transcripts (GLTs). Bottom: Perturbation of key regulators disrupts these outcomes. PRDM1 knockout (PRDM1-KO) prevents PC differentiation and biases cells toward a GC-like state, while IRF4 knockout (IRF4-KO) impairs both PC formation and proper GC responses, highlighting the central role of IRF4 in coordinating B cell fate decisions.

Together, our findings support a model (Fig. 6) in which a steady increase of IRF4 activity during activation in memory B cells drives PRDM1 and exclusive differentiation into plasma cells. This activity of IRF4 appears to suppress germline IgH transcript production, thereby reducing the probability of class switching. In naive B cells, however, IRF4 activity increases during early activation but then declines, remaining active only in the subset of cells that commit to the plasma cell fate. Since our system relies on polyclonal stimulation, we can exclude that this is a consequence of cell selection. Thus, this bifurcation likely results from stochastic oscillations in IRF4 expression. Finally, as IRF4 has been shown to interact with PU.1 (SPI1) and drive GC fate, its absence may allow PU1 activity to somewhat drive GC identity; however, the resulting cells are not equivalent to GC cells, highlighting the indispensable role of IRF4 in coordinating normal GC and plasma cell differentiation. This role of IRF4 is independent of PRDM1, as evident by the fact that PRDM1 KO does not impair GC cells.

## Discussion

Charting the GRNs that control naive and memory B cell differentiation is critical for understanding immune responses to infection, vaccination and autoantigens. In this study, we integrated sc-RNAseq and BCR-seq and investigated the cell fate decisions made by human naive and memory B cells. Strikingly, memory B cells almost exclusively differentiated into plasma cells, while naive B cells had a bifurcated differentiation and generated two distinct cell states. One differentiation branch resulted in plasmablasts and plasma cells that clustered with memory counterparts, while the other resulted in a germinal center (GC) phenotype. By analyzing gene expression dynamics over six days of stimulation, we mapped the GRNs active at different stages of B cell activation. We showed that memory B cells had a more robust IRF4 activity upon activation than naive B cells, a phenomenon already observed in the first 24 h of activation. Previous work in mice demonstrated that transient IRF4 expression results in co-binding with PU.1 (SPI1) at AICE motifs to promote GC formation, but sustained expression results in shifts to lower-affinity ISRE motifs associated with plasma cell genes (Ochiai et al, 2013; Shao et al, 2024). In line with this, we demonstrated that IRF4 is a key network responsible for the more efficient differentiation of memory than naive B cells towards plasma cells. On the other hand, in naive B cells, its expression rises more slowly during early activation but then declines, persisting only in those committing to the plasma cell fate. Because our polyclonal stimulation system excludes selection effects, this bifurcation likely arises from stochastic fluctuations in IRF4 expression.

We hypothesized that knocking out PRDM1 and its upstream regulator IRF4 would rewire the B cell transcriptome, blocking plasma cell differentiation and favoring a GC fate. Interestingly, in IRF4-knockout cells, both naive and memory B cells gave rise to a population resembling GC cells, although this state was notably distinct from the GC cells observed in controls. We therefore hypothesize that this state represents an aberrant GC differentiation. The fact that IRF4 is essential not only for plasma cell differentiation but also for aspects of the GC program aligns with previous observations that IRF4 can dimerize with PU.1 (SPI1) to activate BACH2 transcription and induction of GC genes (Carotta et al, 2014; Ochiai et al, 2024). Consistently, we observed an anticorrelated activity pattern between the IRF4 and SPI1 (PU.1) regulatory networks, with SPI1 (PU.1) activity completely silenced during the terminal differentiation of naive B cells into plasma cells. On the other hand, PRDM1-knockout naive and memory B cells had impaired plasma cell generation as expected, but strikingly produced GC cells highly similar to those in the control condition. These results demonstrate that although memory B cells are intrinsically biased toward plasma cell differentiation, they retain the capacity to adopt a GC fate when PRDM1 activity is suppressed, opening new avenues to direct memory cells to the GC state. While our integrative single-cell transcriptomic and clonal-tracking approach provides a comprehensive view of the gene regulatory networks underlying B cell fate decisions, it should be acknowledged that GRN inference from scRNA-seq data is inherently correlative. Although our knockout experiments for IRF4 and PRDM1 support their involvement in shaping differentiation trajectories, these perturbations do not establish direct regulatory interactions between transcription factors and specific target genes within the predicted networks. Future work employing genome-wide TF binding assays (e.g., CUT&RUN, CUT&Tag, and ChIP-seq), paired perturbation and reporter assays, or high-throughput CRISPR screens will be required to definitively map causal regulatory relationships.

Stochastic fluctuations in the expression of transcription factors at the single-cell level could explain why in our clonal tracking experiment, cells originating from the same ancestral cell (i.e., a clone) were observed to adopt both PC and GC fate. This finding aligns with previous observations in mice, where one cell can give multiple fates (Taylor et al, 2015). The authors showed that the ability of B cells to differentiate into these subtypes was linked to the affinity of their BCRs. Importantly, our single-cell transcriptomic profiling demonstrated that even in the absence of antigen affinity differences, a single naive B cell exhibits transcriptional plasticity, enabling it to differentiate into multiple fates. This suggests that intrinsic gene regulatory network dynamics, rather than external antigen cues alone, contribute to fate determination. Interestingly, we found a correlation between clonal size and clonal composition - smaller clones tended to have a higher proportion of GC cells, suggesting that proliferation capacity may vary even among cells from the same clone.

Our integrative analysis revealed that cells within the same clone share distinctive gene expression profiles, allowing us to accurately reconstruct clonal families solely based on their transcriptomes. This indicates that even under polyclonal stimulation and in the absence of selection, clonal identity is imprinted in their gene expression landscapes, reflecting heritable transcriptional programs. In fact, gene expression alone was sufficient to identify clonal families, using machine learning classification algorithms. Heritable gene expression patterns within clonal families have also been reported in other immune cell types, such as T cells and NK cells (Rückert et al, 2022; Mold et al, 2024). These findings support the hypothesis that the final composition of cell subsets within a clone may be an intrinsic property of that clone. One can further hypothesize that initial differences in signaling dynamics during activation, oscillation in IRF4 expression, or proliferation kinetics may contribute to shaping the ultimate distribution of B cell fates within each clone, adding an additional layer of variability to the B cell immune response. This heterogeneity adds a critical layer of complexity and variability, underscoring the importance of single-cell genomics in unraveling clonal fate decisions.

Next, we computationally distinguished between antibody transcripts that are sterile or encode for secretory and membrane isoforms. Interestingly, when comparing cell populations, over 75% of GC cells expressed germline (sterile) transcripts, while this was true for only about 25% of plasma cells. Despite this strong difference in GLT expression, there was no corresponding difference in the proportion of cells that had undergone class switching between the two populations. This aligns with the fact that while GLT expression is necessary for class switching, it is not sufficient to drive the process. Moreover, we observed that IRF4 knockout cells had a marked increase in GLT levels. This implies that high IRF4 activity may act to suppress GLT expression, adding a potential regulatory role for IRF4 in controlling the accessibility of IgH regions.

Finally, this led us to investigate whether isotype switching is clonally regulated by comparing unswitched cells from both switched and unswitched clones. We found no transcriptional differences between the two groups. Ultimately, this implies that being part of a clonal lineage that has already undergone switching does not confer an increased predisposition toward further switching. These results support the idea that isotype switching is a stochastic and cell-intrinsic process, rather than one governed by a shared transcriptional trajectory within a clone. This independence for CSR among clonally related cells aligns with and provides further experimental evidence for models (Horton et al, 2022) that describe isotype switching as a probabilistic process occurring independently across individual cells within a clone. Our results also likely explain why isotype switching in early GC B cells is highly variable in vivo and highlight the importance of integrating clonal lineage tracking with transcriptomic profiling to dissect the mechanisms of antibody diversification.

Taken together, our findings support a model in which each individual cell is governed by a unique combination of gene programs that regulate cell state, clonal gene expression, and isotype switching, maximizing the breadth of diversity in the immune response.

## Methods

**Table:** Reagents and tools table

Reagent/resource	Reference or source	Identifier or catalog number
**Experimental models**		
Naive, memory, total B cells *(H. sapiens)*	Samples were sourced in accordance with the terms of the informed consents under an approved protocol (ID 3441, study number 6619).	
**Recombinant DNA**		
**Antibodies**		
Mouse anti-CD19-BV786	BD Biosciences	Cat# 563325
Human recombinant anti-CD27-VioBright B515	Miltenyi	Cat# 130-120-028
Human recombinant anti-IgD-VioBrightR720	Miltenyi	Cat# 130-128-838
Human recombinant anti-CD20-APC	Miltenyi	Cat# 130-111-339
Mouse anti-IgG-PE	Miltenyi	Cat# 130-119-878
Human recombinant anti-IgA-PerCPVio700	Miltenyi	Cat# 130-116-885
Mouse anti-IgM-BUV395	BD Biosciences	Cat# G20-127
anti-CD20-Viobright B515	Miltenyi	Cat# 130-111-343
anti-IRF4-eFluor 450	Thermo Fisher Scientific	Cat# 48-9858-80
anti-PU.1-PE	BioLegend	Cat# 658010
**Oligonucleotides and other sequence-based reagents**		
SgRNA HuIRF4: CACGCGGGGCAUGAACCUGG-GCGCGGUGAGCUGCGGCAAC-AGAGCAUCUUCCGCAUCCCC	EditCo Bio	
SgRNA HuPRDM1: GUUGGCAGGGAUGGGCUUAA- GAAGUGGUGAAGCUCCCCUC- CUCUCCCCGGGAGCAAAACC	EditCo Bio	
SgRNA PPP1R12C: CUCCAGGUUCUCAUCAAUGC- GUGGCUACCUAGAUAUCGCC- GUUGUCUGCCUGGUUCACAG	EditCo Bio	
PCR primers huIRF4: TCGTGGTCACTGGCGCA- ACGCCACCTGATGCCTC	EditCo Bio	
PCR primers huPRDM1: CGCCCTGATTTCTGCTGATTC- CATGTTATTAGTTCAAAGGGGCAG	EditCo Bio	
PCR primers PPP1R12C: CTTCAGCAGCCCCTCCATG- CCAGGCGTATCTTAAACAGCC	EditCo Bio	
Sequencing primer PRDM1: TCTGGCTAAACTGATTGGATTCT	This study	
Sequencing primer IRF4: GTGCCCGGAGTGCGGTGC	This study	
Sequencing primer PPP1R12C	EditCo Bio	
**Chemicals, Enzymes and other reagents**		
ZombieNir (849/876 nm) live-dead	BioLegend	Cat# 423105
Live/Dead Fixable Near IR (876 nm) Viability Dye	Thermo Fisher Scientific	Cat# L34981
Fixable Viability Dye eFluor 780 nm	Thermo Fisher Scientific	Cat# 65-0865-18
Brilliant Stain Buffer	BD Biosciences	Cat# 563794
IC Fixation Buffer (Thermo Fisher Scientific)	Ebioscience	Cat# 00-8222-49
CellTraceViolet Cell Proliferation Kit	Thermo Fisher Scientific	Cat# C34557
DRAQ5 Fluorescent Probe Solution	Thermo Fisher Scientific	Cat# 62254
Transcription Factor Buffer Set	BD Bioscience	Cat# 562574
True-Nuclear Transcription Factor Buffer Set	BioLegend	Cat# 424401
HEPES 1 M	Thermo Fisher Scientific	Cat# 15630080
STEMPRO-34 Sfm	Thermo Fisher Scientific	Cat# 10639011
RPMI 1640 HEPES	Thermo Fisher Scientific	Cat# 22400089
megaCD40L	Enzo Life Science	Cat# EN522110C010
AffiniPure F(ab’)₂ Fragment Goat Anti-Human IgM, Fc5μ fragment specific	Jackson ImmunoResearch	Cat# JI109006129
IL-21	PeproTech	Cat# 200-21-10uG
IL-2	PeproTech	Cat# 200-02-10uG
penicillin/streptomycin	Thermo Fisher Scientific	Cat# 15140122
EDTA	pluriSelect	Cat# 60-00030-11
NaN3	Merck	Cat# S2002-25G
**Software**		
Python and R environments		https://github.com/SoskicLab/bcelltimecourse
FlowJo™ v Software v10	BD Life Sciences	
IDEAS 6.3 Image Analysis Software	Cytek Amnis	
**Other**		
EasySep™ Human B Cell Isolation Kit	StemCell Technologies	Cat# 17954
EasySep™ Human Naive B Cell Isolation Kit	StemCell Technologies	Cat# 17254
Gene Knockout Kit v2 (Multi-guide) Genome: Homo Sapiens: PRDM1	EditCo. Bio	Cat# GKO_HS2_00001
Gene Knockout Kit v2 (Multi-guide) Genome: Homo Sapiens: IRF4	EditCo. Bio	Cat# GKO_HS2_00001
Gene Knockout Kit v2 (Multi-guide) Genome: Homo Sapiens: PPP1R12C	EditCo. Bio	Cat# GKO_HS2_00001
Cas9 Nuclease 2NLS	EditCo. Bio	Cat# CAS9 2NLS
QIAamp UCP DNA Micro Kit	Qiagen	Cat# 74004
QIAquick PCR purification kit	Qiagen	Cat# 28104
P3 Primary Cell 4D-Nucleofector X Kit	Lonza	Cat# LOV4XP3032
Platinum™ SuperFi II PCR Master Mix	Thermo Fisher Scientific	Cat# 12368010
10X Genomics Chromium Next GEM Single Cell 5’ v2 Kit	10x Genomics	
Cytek Amnis ImageStream MkII Flow Cytometer	Cytek Biosciences	
CytoFLEX LX	Beckman Coulter	
MoFlo Astrios cell sorter	Beckman Coulter	
NovaSeq 6000	Illumina	

### Naive and memory B cell isolation

Human biological samples were sourced ethically, and their research use was in accordance with the terms of the informed consents under an approved protocol (ID 3441, study number 6619). The experiments were conducted following the principles set out in the WMA Declaration of Helsinki and the Department of Health and Human Services Belmont Report. Peripheral blood mononuclear cells (PBMCs) were isolated from healthy blood donors using Ficoll-Hypaque density gradient centrifugation. Prior to B cell isolation, PBMCs were incubated overnight (12 h) at 5 million cells/mL in RPMI 1640, HEPES and glutamine supplemented with 10% fetal bovine serum and penicillin/streptomycin (Thermo Fisher Scientific). Total B cells were then isolated by negative selection using the EasySep™ Human B Cell Isolation Kit (Stemcell Technologies). B cells were stained in DPBS with eeFluor780-Live/Dead dye (Thermo Fisher Scientific) for 10 min at 4 °C according to the manufacturer’s instruction followed by washing with FACS buffer (1 x DPBS, 3% FBS, 1 mM EDTA (Merck)) and staining with surface fluorochrome-labeled primary antibodies for 20 min at 4 °C. The following antibodies were used: anti-human CD19-BV786 (BD Bioscience), anti-human CD27-VioBright B515, CD20-APC, IgG-PE, and IgA-PerCP-Vio700 (Miltenyi). Cells were washed with HBSS, 2% FBS, 10 mM Hepes, resuspended in the same buffer and sorted using a MoFlo Astrios EQ cell sorter (Beckman Coulter, USA).

### In vitro B cell activation

Sorted naive and memory B cells were cultured in complete StemPro-34 SFM medium supplemented with 2 mM glutamine and 100 U of Pen/Strep. 25,000 cells were seeded in 600 μl of media in 48-well plate wells. Sorted B cells were activated in complete StemPro-34 medium with 200 ng/mL megaCD40L (Enzo Life Science), 5 µg/mL AffiniPure F(ab’)2 Fragment Goat Anti-Human IgM, Fc5 Fragment Specific (Jackson ImmunoResearch), 100 ng/mL IL-21, and 20 ng/mL IL-2 (PeproTech, Thermo Fisher Scientific) at 37 °C. At the designated time points, live cells were sorted in for the scRNA-seq. For scRNAseq, naive and memory B cells from four healthy donors were independently activated. No blinding was performed. The sample size was estimated to be appropriate for generating a detailed reference map of B cell activation and is in line with sample sizes used in comparable single-cell studies.

### Clonal culture

Naive B cells were isolated and sorted as described above. About 2000 naive B cells (live, CD19⁺ CD20⁺ CD27⁻ IgG⁻ IgA⁻) from two donors were sorted directly into 96 round-bottom well plates. Cells were cultured in 200 μl in complete StemPro-34 medium, and B cells were stimulated as described above. At day 6, cell count and viability were determined with the Trypan blue staining.

### CRISPR-CAS9 gene knock-outs

Synthego’s IRF4, PRDM1, and AAVS1 (within PPP1R12C gene) Gene Knockout Kits v2, along with Cas9 2NLS, were supplied by EditCo Bio. Briefly, naive and memory B cells were sorted from pre-enriched total B cells of two healthy volunteers, as previously described, using a MoFlo Astrios cell sorter (Beckman Coulter). Sorted B cells were activated in complete StemPro-34 medium with 200 ng/mL megaCD40L (Enzo Life Science), 5 µg/mL AffiniPure F(ab’)2 Fragment Goat Anti-Human IgM, Fc5 Fragment Specific (Jackson ImmunoResearch), 100 ng/mL IL-21, and 20 ng/mL IL-2 (PeproTech, Thermo Fisher Scientific) at 37 °C for 20 h. Cells were collected and electroporated at 1 × 10^5^ cells for each target gene with the sgRNA-Cas9 mixture at a 9:1 ratio (180 pmol:20 pmol) using the P3 Primary Cell 4D-Nucleofector X Kit (Lonza) following the manufacturer’s instructions and rested in complete StemPro-34 medium at 37 °C for 30 min. Naive and memory B cells were activated as described above and cultured for a further 5 days. On day 6, cells were collected and processed for scRNA and paired with scVDJ using the 10X Genomics Chromium Next GEM Single Cell 5’ v2 Kit. The efficiency of CRISPR was assessed by flow cytometry, qualitative PCR and Sanger Sequencing. sgRNA multi-guide sequences, provided by Editco. Bio, are: huIRF4: CACGCGGGGCAUGAACCUGG, GCGCGGUGAGCUGCGGCAAC, AGAGCAUCUUCCGCAUCCCC; huPRDM1: GUUGGCAGGGAUGGGCUUAA, GAAGUGGUGAAGCUCCCCUC, CUCUCCCCGGGAGCAAAACC; PPP1R12C: CUCCAGGUUCUCAUCAAUGC, GUGGCUACCUAGAUAUCGCC, GUUGUCUGCCUGGUUCACAG.

### PCR analysis of gene knockout

DNA was isolated from human B cell pellets using QIAamp UCP DNA mini spin columns (Qiagen), following the manufacturer’s instructions. EditCo.Bio-supplied PCR primer sequences were utilized for the PCR: huIRF4: TCGTGGTCACTGGCGCA, ACGCCACCTGATGCCTC, amplicon size 500 bp; huPRDM1: CGCCCTGATTTCTGCTGATTC, CATGTTATTAGTTCAAAGGGGCAG, amplicon size 500 bp; PPP1R12C: CTTCAGCAGCCCCTCCATG, CCAGGCGTATCTTAAACAGCC, amplicon size 475 bp. Briefly, 100 ng of isolated DNA was amplified using the Platinum SuperFi PCR-ready-to-use master mix (Thermo Fisher Scientific) according to the manufacturer’s instructions. Samples were processed using the VerityPro 96-well thermocycler from Applied Biosystems, using the following cycling conditions: 98 °C for 30 s, 30 cycles of 98 °C for 10 s, 60 °C for 10 s, 72 °C for 15 s, and 72 °C for 5 min. Amplicons were visualized on a 2% agarose gel. PCR products of the targeted gene regions from CRISPR-edited cells were purified using a QIAquick PCR Purification Kit (Qiagen) and quantified using the Thermo Scientific NanoDrop One Spectrophotometer. Purified amplicons from CRISPR-edited cells were verified by Sanger sequencing (Eurofins Genomics).

### Flow cytometry

For surface staining, cells were washed with PBS and incubated with Live/Dead Fixable Near IR (876) Viability dye (Thermo Fisher Scientific), diluted 1:2000 in PBS, for 15 min on ice. Cells were then washed with a staining buffer (PBS, 3% FBS, and 0.05% Sodium Azide) before being incubated for 30 min at 4 °C with an antibody mix targeting cell surface markers. Cells were then fixed with IC Fixation Buffer (Thermo Fisher Scientific) for 1 h at 4 °C. For intracellular markers, cells were permeabilized using either the Transcription Factor Buffer Set (BD Bioscience) or the True-Nuclear Transcription Factor Buffer Set, in accordance with the manufacturer’s instructions (BioLegend). Cell Trace Violet was prepared according to the manufacturer’s instructions (Thermo Fisher Scientific), and the solutions were warmed to 37 °C. An equal volume of CTV in PBS was added to the cell suspension to achieve a final concentration of 2.5 µM dye (1:2000). The incubation was performed at 37 °C and 5% CO_2_ for 20 min, protected from light. The dye was quenched by adding three times the original volume of RPMI 1640 HEPES-Glutamine medium, which contained 10% FBS, 100 U of penicillin/streptomycin, followed by a 5-min incubation at RT. Samples were run on a CytoFLEX LX (Beckman Coulter). Data analysis were performed using the FlowJo data analysis software package (TreeStar, USA). Immunophenotyping included: ZombieNir (849/876 nm) live-dead, CD19-BV786, CD27 Vio Bright B515, IgD Vio Bright R720, CD20-APC, IgG-PE, IgA-PerCPVio700, IgM-BUV395. Data were analyzed with FlowJo and were imported in R (v4.3.1) for statistical analysis.

### Intracellular staining and ImageStream analysis

Naive B cells were isolated by negative selection using magnetic beads (StemCell Technologies) from PBMC after overnight resting in complete RPMI 1640, HEPES medium (Thermo Fisher Scientific) supplemented with 10% FBS and 100 µg/mL of penicillin and streptomycin. 1 × 10^5^ cells per well in a 24-well plate were activated with the previously described B cell stimulation cocktail for 6 days in complete STEMPRO-34 medium. On day 6, 5 × 10^5^ cells were collected, washed in PBS, and stained with Fixable Viability Dye eFluor 780 (Thermo Fisher Scientific) for 10 min at 4 °C. The cells were washed with staining buffer and surface-stained for 30 min with anti-human CD20 Viobright B515 (Miltenyi), followed by intracellular staining with anti-human/mouse IRF4 eFluor 450 (Thermo Fisher Scientific) and anti-human PU.1 (SPI1) PE (BioLegend) monoclonal antibodies using the True-Nuclear Transcription Factor Buffer set (BioLegend) as per the manufacturer’s instructions. Briefly, the cells were fixed in True-Nuclear 1x-Fix Buffer at room temperature for 45 min, washed twice with True-Nuclear 1x-Perm Buffer and then resuspended in 100 µl of 1x Perm Buffer. After blocking for 5 min with Human TrueStain FcX (Biolegend), the conjugated antibodies were added, and the sample was incubated for 30 min at room temperature. At the end of the incubation, 0.25 µl of DRAQ5 nuclear staining (Thermo Fisher Scientific) in 500 µL of 1x Perm was added to the sample for 5 min. The sample was washed with 1X Perm and resuspended in 25 µL of Staining Buffer. Sample acquisition was performed using the Cytek Amnis ImageStream MkII Flow Cytometer; data analysis was conducted with the IDEAS Image Analysis Software.

### scRNA-seq library preparation

At each time point, naive and memory B cells were collected and were stained with eFluor 780-Live/Dead. Live cells were sorted with the MoFlo Astrios FACS sorter. Naive B cells and memory B cells deriving from two different donors and from the same time point, were pooled together in one single cell 10x reaction. This single-cell experiment configuration was replicated for each time point of the analysis. Single cells were encapsulated into Gel Emulsion droplets using the Chromium Controller system (10x Genomics), targeting 20,000 cells for each sample and 5’ Gene Expression and BCR libraries were generated in accordance with the protocol ChromiumNextGEMSingleCell5-v2 CG000331 (10x Genomics). Libraries were pooled at a ratio dependent on the 10x Genomics indication and loaded on the Novaseq6000 sequencer to achieve a minimum of 50,000 reads/cell for each Gene Expression library and 5000 reads/cell per BCR library.

### scRNA-seq preprocessing and clustering of the time course experiment

Sequencing reads were aligned to the refdata-gex-GRCh38-2020-A reference provided by 10x Genomics and quantified with CellRanger (v7.0.1). Individual samples were demultiplexed by first genotyping each cell with cellSNP-lite using the SNP reference file provided by the cellSNP-lite authors, genome1K.phase3.SNP_AF5e2.chr1toX.hg38.vcf.gz and keeping SNPs with MAF >0.1 with at least 20 UMIs (Huang and Huang 2021). Genotype deconvolution was performed with VireoSNP with default parameters (Huang et al, 2019).

Background RNA was removed from the count matrices with cellbender (v0.2.0)(Fleming et al, 2023).

Quality control, doublet removal, filtering, clustering, dimensionality reduction, data inspection was performed in Python (v3.12) using the Scanpy package (v1.10.1). Quality control was performed per sample by filtering out cells with low number of counts, low number of genes, and high fraction of mitochondrial genes using adaptive thresholds. Thresholds were identified with the median (*n* = 3, 4, 5) absolute deviation method and further refined by manually inspecting each threshold on each sample and donor (‘Orchestrating Single-Cell Analysis with Bioconductor’, n.d.; Heumos et al, 2023). Doublets were identified and removed with scrublet (‘Scrublet: Computational Identification of Cell Doublets in Single-Cell Transcriptomic Data’ 2019). Scrublet was run with default parameters for each sample and the threshold separating doublets from singlets was manually selected by comparing the distribution of simulated doublets with the doublets identified with VireoSNP (Dataset EV10). Droplets assigned to multiple donors or unassigned were removed.

Before dimensionality reduction, for each cell, counts were first normalized over the total counts over all genes, then multiplied by 10,000, and lastly the data was log transformed. About 3000 highly variable genes were identified with the implementation of the dispersion-based ‘seurat’ method in scanpy (flavor = “seurat”). Before dimensionality reduction, we removed from the variable features all immunoglobulin variable genes as well as the heavy constant and light constant chains. Dimensionality reduction was performed with principal component analyses performed using the top 3000 highly variable genes and cells were clustered with Leiden clustering based on the first 30 principal components. Quality control was repeated after the first clustering iteration, as clusters of low-quality cells were identified. These clusters consisted of cells with a low fraction of ribosomal genes and high mitochondrial gene fractions, thus they were removed. Donor-dependent effects were integrated using Harmony on the first 30 principal components (Korsunsky et al, 2019). We performed clustering with the Leiden algorithm on the first 30 Harmony dimensions. Several clustering resolutions were inspected, and we selected the lowest clustering resolution (resolution = 1.20) that resolved naive and memory B cell types at each time point. Finally, after quality control, our time course dataset consisted of 118.891 cells from four healthy human donors.

### scRNA-seq preprocessing and clustering of the CRISPR-Cas9 gene knock-out experiments

Demultiplexing, quality control and doublet removal was performed as described above (Dataset EV10). After the first round of clustering, clusters with low fractions of ribosomal genes and high mitochondrial fractions were identified and removed. The final dataset consisted of 56,571 naive and memory cells from two healthy human donors.

### scRNA-seq preprocessing and clustering of the clonal experiments

Preprocessing and quality control was performed as before (Dataset EV10). Doublets were detected with scrublet by setting a manual threshold on the simulated doublets dataset (Dataset EV10). Finally, after quality control, our clonal dataset consisted of 23,484 naive B cells from two different human healthy donors.

### sciCSR

Germline transcripts were quantified using sciCSR (v0.3.1) with default parameters using the hg38 germline transcripts definitions provided by the authors (Ng et al, 2023).

### Differential gene expression pathway enrichment and annotation

Differential gene expression was performed using the edgeR package (v4.10.16). Anndata objects were converted to Seurat (5.0.1) objects in R (4.3.1). UMI counts were pseudo-bulked per donor and condition, and genes were tested if their aggregated sum was at least 30 UMIs. Differential expression was tested with the glmTreat method by testing if the genes were differentially expressed by at least 20% between conditions, accounting for the donor effect. To identify relevant pathways enriched in the different conditions tested, we performed overrepresentation analysis using the goprofiler2 (v0.2.3) R package, and tested for enrichment in pathways from the GO, KEGG and Reactome databases. Competitive gene set tests were performed with the camera method in the edgeR package (Wu and Smyth 2012) using GO, KEGG and Reactome databases. All datasets were manually annotated by computing differential gene expression between all clusters and inspecting the top 400 upregulated and downregulated differentially expressed genes. CRISPR-Cas9 knock-out manual annotations were compared to a semi-supervised annotation with single-cell ANnotation using Variational Inference (scANVI). We performed scANVI (Xu et al, 2021) annotation as described here (https://docs.scarches.org/en/latest/scanvi_surgery_pipeline.html) with scarches (v0.6.1) using our timecourse manually annotated cells as reference.

### scVDJ-seq preprocessing

scVDJ libraries were aligned with CellRanger (v7.0.1) to the refdata-cellranger-vdj-GRCh38-alts-ensembl-5.0.0 reference. The Immcantation and (Gupta et al, 2015) and the Dandelion (v0.5.2) (Suo et al, 2023) frameworks were used for the identification of the alleles, isotypes, and to assign cells to clonal families. We started from the unfiltered contigs generated from cellranger and we used the dandelion to run IgBlast to genotype the VDJ alleles in each cell. When cells with multiple VDJ contigs were found, for heavy and light chains, we retained the contig with the highest expression if its expression was three times higher than the summed expression of the other contigs in the same cells. Otherwise, the cell was marked as “no contig” and was not considered for further VDJ and clonal analysis. Additionally, chains with less than three UMIs were filtered out. Cells were grouped into clonal families using the Immcantation pipeline. We measured the Hamming distance between the nucleotide sequences of the VDJ-junctions and calculated a threshold for grouping VDJ-sequences into the same clonal family. Thresholds were calculated per donor, and the mean of the threshold were used as the two donors yielded highly similar thresholds (0.12, 0.08). Cells were then grouped into clonal families if they shared the same VDJ alleles. If two cells had the same heavy chain but different light chain, the cells were further separated into two different clonal families.

### Clonal correlation of gene expression and classification

For the gene expression analysis of the clonal dataset preprocessing and quality control was performed as before. Finally, after quality control, our clonal dataset consisted of 23,484 naive B cells from two different donors. For intraclonal and interclonal correlation, only clones with at least four cells were considered and at least one cell had to be IGHM+. Interclonal and intraclonal correlation were calculated separately per donor. Only genes expressed in at least 300 cells were used. V(D)J genes were excluded. 10.000 random pairs of cells were randomly sampled from clones and the Pearson correlation coefficient was calculated for each interclonal and intraclonal pairs. To calculate clonal and interclonal correlations and to classify cells into clonal families, counts were normalized over the total counts, then multiplied by 10,000 and log transformed. We trained three different machine learning algorithms (neural network, multiclass logistic regression and a random forest) to assign cells to their clonal families using only gene expression values. As input features, we first selected the genes whose interclonal correlation was *r* > 0.25 and expressed in at least 40% of cells for each donor. All immunoglobulin genes were removed from the input features. The union of the top correlated genes of the donors was then used, resulting in a final set of 369 genes. Input gene expression values were normalized as described above and scaled to a unit of variance and a mean of 0. To train the classifiers, we took clones with at least 25 cells. Model performances were assessed with a fivefold cross-validation method by randomly partitioning the dataset into five equal subsets. For each iteration (fold), four subsets were used as training and classification accuracy was estimated on the held-out fold. The metrics reported are the average of the five runs (K = 5). Additionally, we used several control sets of input features and clonal labels: (1) Random sets of the same number of genes sampled from genes with clonal correlation lower than 0.25 and we reported the model performance metrics as the average of 100 random samplings; (2) we used normalized genes expression values where cell state (XBP1, IRF4 and SPI1 regulon scores) and cell cycle gene (S phase and G2M phase scores) expression values were regressed out; 3) Clonal labels were randomly shuffled per donor 1000 times and the random distribution of accuracy scores was compared with the measured ones.

The multinomial logistic regression model was implemented in scikit-learn (v1.5.2) using the lbfgs solver, balanced class weighting and with 1000 maximum iterations. The feed-forward fully connected neural network was implemented in PyTorch Lightning (v2.5.0). The network consisted of a total of four layers: an input layer with as many nodes as the number of input genes, two hidden layers with 1028 nodes each, and a latent layer with 256 nodes, followed by an output layer whose size matched the number of clonal families. The rectifier linear unit was used as the activation function and a dropout of 0.4 was used to avoid overfitting and improve generalization. Cross-entropy was used as a function to calculate the loss. The model was trained across 50 epochs using the Adam optimizer with a learning rate of 0.005. The random forest classifier was implemented in scikit-learn (v1.5.2). Hyperparameters were identified using grid-search with fivefold cross-validation using accuracy as the scoring metric. The hyperparameter grid included: n_estimators (100, 300, 500, and 1000), max_depth (None, 10, 20, 30, and 90), min_samples_split (2, 5, 10, and 40), min_samples_leaf (1, 2, 4, and 10), max_features (auto, sqrt). We selected the combination of hyperparameters yielding the highest accuracy.

### Clonal analysis of Agrafiotis et al, 2023

We downloaded a publicly available 5’ scRNA-seq and scVDJ dataset of bone marrow B cells from 11 different mice immunized with human TNFR2 mixed with 20 μg of the adjuvant monophosphoryl lipid A (MPLA) (Agrafiotis et al, 2023) (ArrayExpress accession number E-MTAB-12610). Sequencing reads were aligned to the refdata-gex-mm10-2020-A reference provided by 10x Genomics with CellRanger (v7.0.1). Quality control of gene expression data was performed as described above (Dataset EV10). scVDJ libraries were aligned to the reference file refdata-cellranger-vdj-GRCm38-alts-ensembl-7.0.0 provided by 10x Genomics with CellRanger (v7.0.1). Clonal assignment was performed as described above with the Immcantation and (Gupta et al, 2015) and the Dandelion (v0.5.2) (Suo et al, 2023) frameworks. Clonal correlation of gene expression was quantified for each mice separately, including genes whose expression was detectable in at least 10% of the cells of that sample. We trained two different machine learning algorithms (a neural network and multiclass logistic regression) to assign cells to their clonal families using only gene expression values. Input genes were selected per mice. Gene expression clonal correlations were generally lower in mice data than in human samples, thus we included genes with an interclonal Pearson correlation above 0.10 and expressed in at least 40% of cells for each mice. All immunoglobulin genes were removed from the input features. The classifier's architecture was described above.

### Isoform quantification with salmon alevin

For transcript-level quantification, we used Salmon/Alevin (v1.10.2) (Srivastava et al, 2019) using a per-isoform evidence-based annotation of the human genome (GRCh38), version 32 (Ensembl 98) from GENCODE. And for differential transcript usage, we used DTUrtle (v1.0.2) (Tekath and Dugas 2021).

### Gene regulatory network inference

GRNs were inferred with pySCENIC (v0.12.1) (Aibar et al, 2017; Van de Sande et al, 2020). To reduce the computational burden and reduce the dropout due to the sparsity of scRNA-seq data, we performed GRN inference on metacells. Metacells were generated per each sample independently with SEAcells v(0.3.3) (Persad et al, 2023) using the post quality control dataset comprising naive and memory B cells at all time points. Briefly, a SEAcell run per cell type—time point—donor was performed; for each combination, counts were normalized over the total counts over all genes, then multiplied by 10,000, and lastly, the data were log transformed. The top 1500 highly variable genes were identified as described previously, and principal component analysis was performed. Metacells were composed of ~75 cells. Metacells inference was performed with the following parameters n_waypoint_eigs = 10 and convergence_epsilon = 1e-5. The number of iterations for reaching model convergence was constrained between 20 and 100. For each SEAcell run, we set the random seed to 42. Lastly, for each metacell, the raw expression counts of all cells were summed to obtain aggregated counts per gene. We obtained 1585 metacells across all samples. Before performing GRN inference, we filtered out genes that were detected in less than three metacells, resulting in 28,691 genes included in the final dataset.

We used the metacells, raw counts as input for GRN inference. As the GRN inference with pySCENIC has a stochastic component, we performed 30 runs of the GRN inference with the grnboost2 algorithm, and we retained the regulons that were detected in 80% of the runs. For the 30 grnboost2 runs, we used random seeds from 1 to 30. To derive the Transcription Factor to gene connection, we used the 10 kbp and 500 bp hg38/refseq_r80/mc_v10 motif definitions made available by the pySCENIC authors. Next, for each regulon, we retained the genes that were assigned to that regulon in 70% of the runs. Regulons with less than ten genes were discarded. Regulon scores were quantified for each cell with AUCell, which normalizes the output of the regulon scores (Aibar et al, 2017). GRN inference was performed with the docker container (v0.12.1) provided by the pySCENIC authors using the arboreto implementation (Van de Sande et al, 2020). We refer to regulons as sets of genes and the transcription factor that controls their expression. Regulon scores are obtained for each cell with AUCell using an AUC threshold of 0.05. To allow comparison of different regulons, we computed Regulon Specificity Scores (RSS) on the raw AUCell scores with the pySCENIC implementation of the Jensen-Shannon distance (Suo et al, 2018). Regulons were ranked by the value of the RSS per cell state, with higher values indicating higher specificity.

### Published dataset regulon scoring

We downloaded publicly available regulon definitions identified on 211 human B cell samples and made available on the bcellViper Bioconductor package (Basso et al, 2005; bcellViper-Bioconductor). We retained regulons with at least 10 genes. Regulon activity was scored with the VIPER method (Alvarez et al, 2016) from the decoupler-py python package (v2.1.1). Before computing regulon specificity scores (RSS), VIPER scores were scaled to have values between 0 and 1. Regulon specificity scores were computed as described above. Regulons were ranked by the value of the RSS per cell state, with higher values indicating higher specificity.

### Transition probability estimation with Moscot

We used optimal transport with Moscot (v0.3.5) (Klein et al, 2025) to estimate lineage relationships across time points as described (https://moscot.readthedocs.io/en/latest/notebooks/tutorials/200_temporal_problem.html#). We preprocessed naive and memory B cells as described above. PCA and harmony embeddings were calculated using the top 3000 highly variable features. We prepared the TemporalProblem by firstly computing the cost matrix based on squared Euclidean distances between cells on the Harmony embeddings. Next, we solved the TemporaProblem with hyperparameters tau_a = 0.90 and tau_b = 0.99. To identify the ancestors and descendants, we aggregated the transport matrix by cell state.

### Pseudotime estimation with Palantir

We used Palantir (v1.4.1) (Setty et al, 2019) to model the trajectories of differentiating cells as described (https://palantir.readthedocs.io/en/latest/notebooks/Palantir_sample_notebook.html). Briefly, naive and memory B cell datasets were preprocessed separately as described above. PCA and harmony embeddings were calculated independently for naive and memory B cells using the top 3000 highly variable features. The diffusion matrices were estimated separately for each cell type using 30 latent dimensions. Early and late cells were detected automatically with the early.cell utility from Palantir, specifying early and late cell states. About 1200 waypoints were used to compute the Palantir pseudotime.

### In silico knock-out with CellOracle

We used CellOracle v(0.20.0) (Kamimoto et al, 2023) to simulate the effect of the transcription factor knock-outs as described (https://morris-lab.github.io/CellOracle.documentation/index.html). GRN models were constructed using only the top 3000 highly variable genes on day 6, naive and memory B cells separately. Briefly, we constructed the gene regulatory networks model using the CellOracle built-in human transcription factor to target gene definitions. TF-gene links were pruned using the following parameters: *p* = 0.001, weight = “coef_abs”, threshold_number = 5000. Transcription factor perturbation was evaluated by setting their expression to 0 and propagation to 5. Simulated perturbation effects were compared by computing the dot product of the vector fields with the vector field of the differentiation flow estimated with Palantir pseudotime on day 6 cells only.

## Supplementary information

Peer Review File

Appendix

Dataset EV1

Dataset EV2

Dataset EV3

Dataset EV4

Dataset EV5

Dataset EV6

Dataset EV7

Dataset EV8

Dataset EV9

Dataset EV10

Source data Fig. 1

Source data Fig. 2

Source data Fig. 3

Appendix Figure S8 Source Data

## Data Availability

The datasets and computer code produced in this study are available in the following databases: Raw scRNA-seq data: European Genome-phenome Archive (EGA) EGAD50000002113 (https://ega-archive.org/datasets/EGAD50000002113), EGAD50000002114 (https://ega-archive.org/datasets/EGAD50000002114) and EGAD50000002115 (https://ega-archive.org/datasets/EGAD50000002115). Raw human genomic data are subject to controlled access in accordance with GDPR and applicable EU regulations to protect the privacy of research participants. These datasets are available through the EGA and can be accessed for ethically approved research and to verify the findings of this study. Detailed terms and conditions governing access to the managed datasets are provided in the “Data Access Agreement” associated with each dataset. Access requests must be submitted through the EGA portal by selecting “Request Access” for the relevant dataset. Requests are reviewed by the Data Access Committee at Human Technopole to ensure compliance with ethical and legal requirements and will receive a response within 2–4 weeks. For any additional information, please contact Blagoje Soskic (blagoje.soskic@fht.org). Processed scRNA-seq data (h5ad): Zenodo (10.5281/zenodo.17984776). Computer scripts: GitHub (https://github.com/SoskicLab/bcelltimecourse). The source data of this paper are collected in the following database record: biostudies:S-SCDT-10_1038-S44320-026-00207-8.
