# Supplementary material for: Competing gene regulatory networks drive naive and memory B cell differentiation
Source: Mol Syst Biol. 2026 Apr 16;22(7):1097–117. doi: 10.1038/s44320-026-00207-8 (PMC13328623; doi:10.1038/s44320-026-00207-8)
Supplement: Supplementary file 2 — Appendix [file 44320_2026_207_MOESM2_ESM.pdf]

Appendix for “Competing gene regulatory networks drive naive and memory B cell differentiation”

Table of Contents

Appendix Figure S1....2
Appendix Figure S2....3
Appendix Figure S3....4
Appendix Figure S4....5
Appendix Figure S5....6
Appendix Figure S6....7
Appendix Figure S7....8
Appendix Figure S8....9
Appendix Figure S9....10
Appendix Figure S10....11
Appendix Figure S11....12
Appendix Figure S12....13
Appendix Figure S13....14
Appendix Figure S14....15
Appendix Figure S15....16
Appendix Figure S16....17
Appendix Figure S17....18
Appendix Figure S18....19

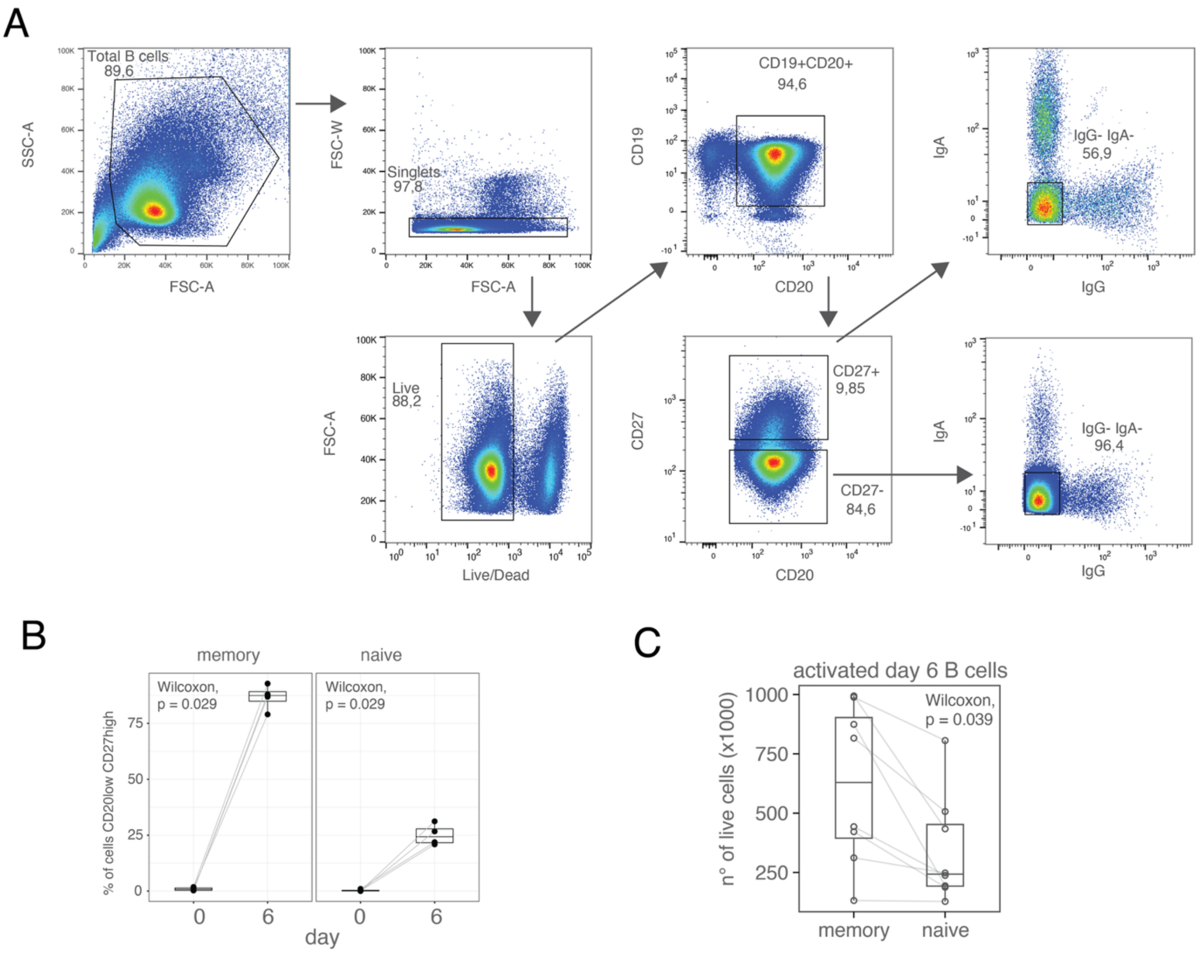

Appendix Figure S1 A) gating strategy for sorting naive (live, CD19+ CD20+ $CD27_{+}$ IgG− IgA−) and memory B cells (live, CD19+ CD20+ $CD27_{+}$ IgG− IgA−) from total B cells. **B**) Fraction $CD27_{high}$ $CD20_{low}$ cells; n=4 biologically independent replicates. P-values were calculated with the Wilcoxon signed-ranked test. In the boxplots, the center line represents the median; boxes indicate the interquartile range (25th–75th percentiles); whiskers extend to the most extreme values within 1.5× the interquartile range. **C**) Total number of live cells at day 6; n=8 biologically independent replicates.

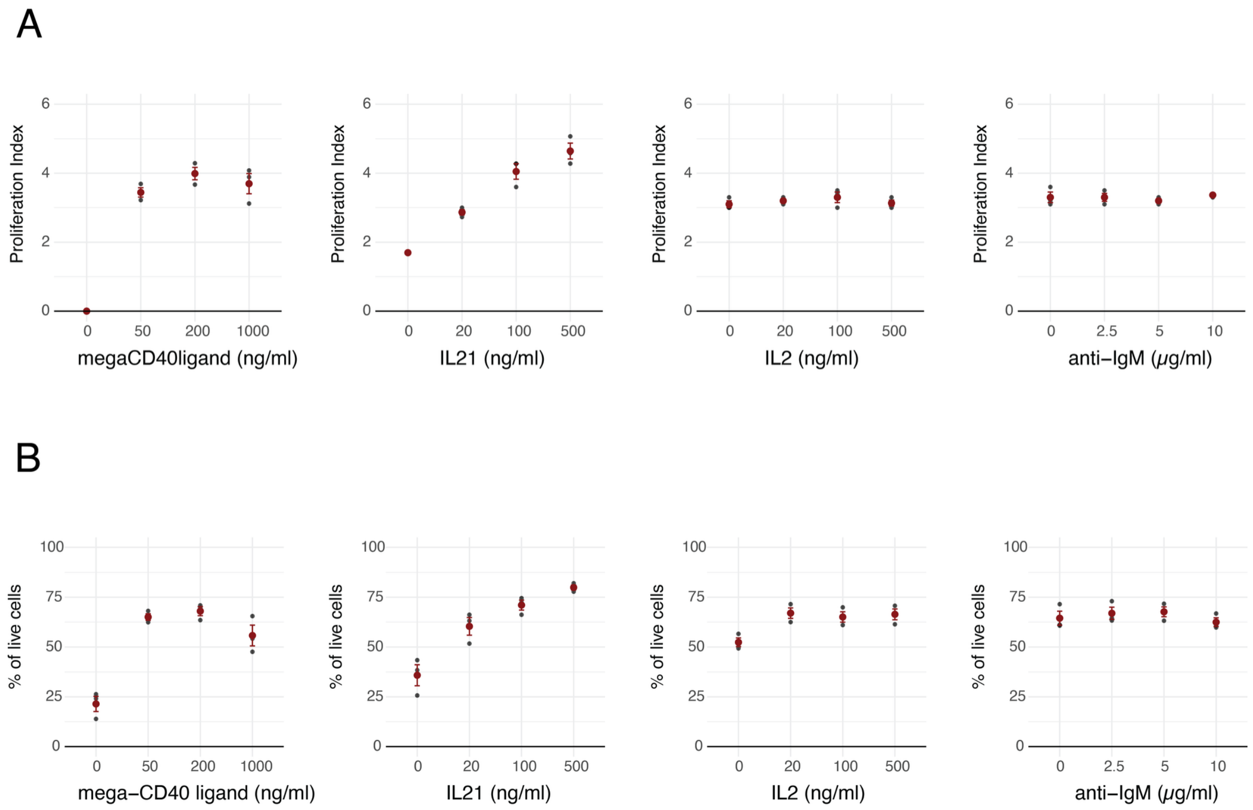

Appendix Figure S2 A-B) Plots illustrating the results of the titration of IL21, IL2, megaCD40ligand and anti-IgM. For each reagent, four different concentrations were tested while keeping the others fixed (200 ng/mL megaCD40L, 5μg/mL anti-IgM, 100 ng/mL IL-21, and 20 ng/mL IL-2). Proliferation index **A**) and percentage of live cells **B**) were quantified. Mean and standard error are shown for 3 different biological replicates.

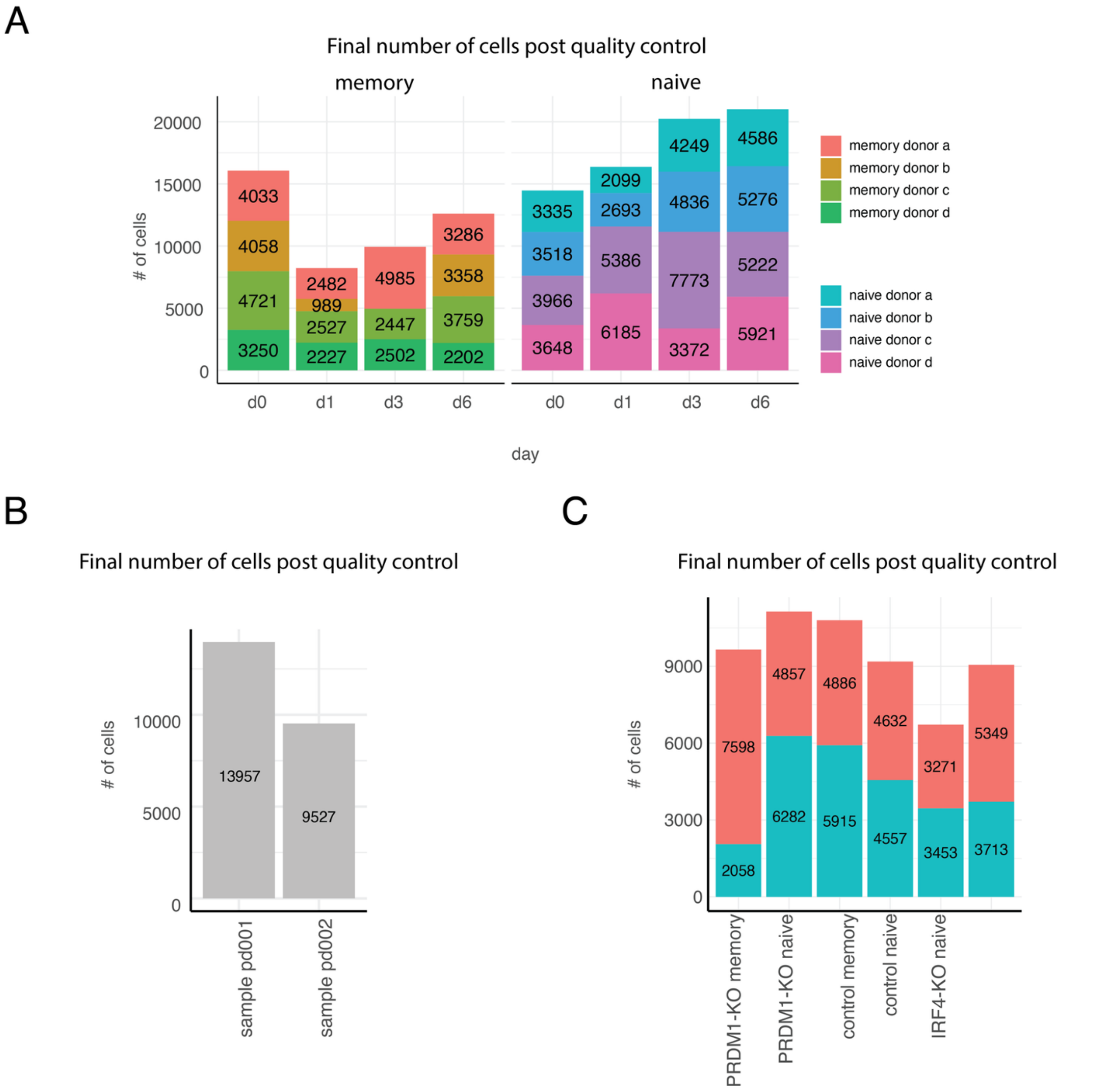

Appendix Figure S3 A) Stacked bar plot showing the total number of cells retained for downstream analysis after applying quality control (QC) filters for each of the four donors in the time course scRNA-seq data. Color represents the donor cell type combination. **B**) Stacked bar plot showing the total number of cells retained for downstream analysis after applying quality control (QC) filters for each of the two donors in the clonal scRNA-seq data. **C**) Stacked bar plot showing the total number of cells retained for downstream analysis after applying quality control (QC) filters for each of the two donors in the CRISPR-Cas9 KO scRNA-seq data

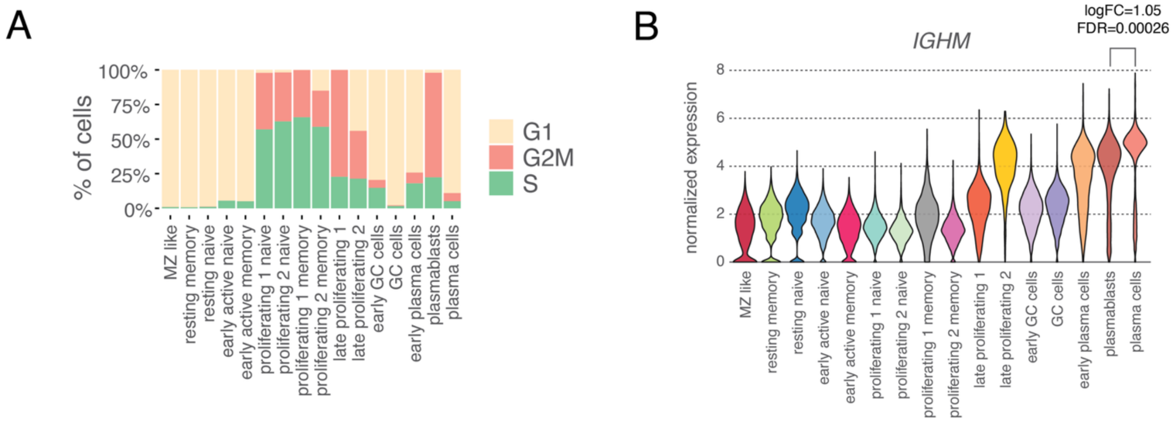

Appendix Figure S4 A) Proportion of cells in different cell cycle phases stratified by cell type and time point. **B**) Normalized expression level of IGHM. Colors represent cell state. Single cell data from n=4 biological replicates.

A

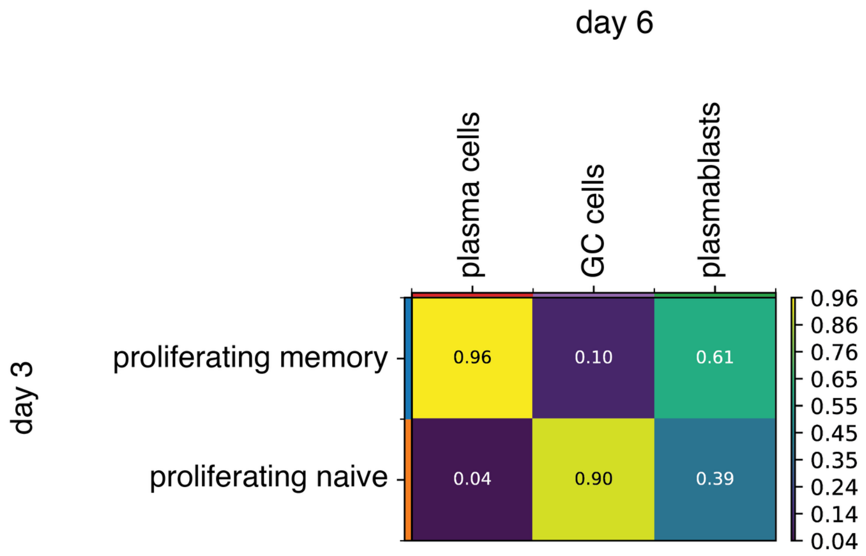

Appendix Figure S5 A) Moscot-calculated transition matrix showing the ancestry of cells at day 6 cells. Ancestor cell states at day 3 are on the rows and descendant cell states at day 6 are on the columns.

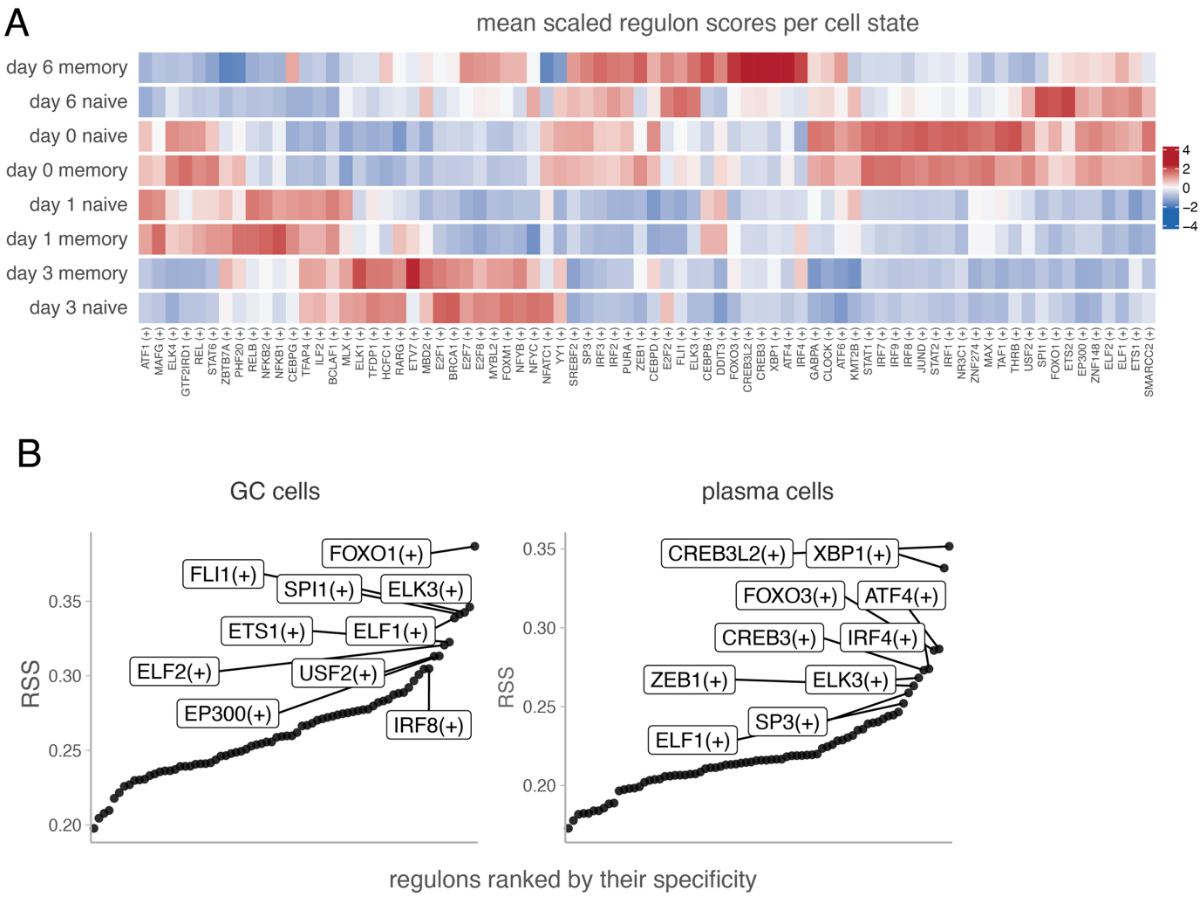

Appendix Figure S6 A) Heatmap of regulon activity per time point and cell type. Columns represent transcription factors. For each condition, mean regulon scores were calculated across all cells and scaled. Blue and red represent score values from lowest to highest. **B**) Regulon specificity scores (RSS) for GC cells and plasma cells. Regulons were ranked by their RSS value in increasing order. For the top 10 regulons, the transcription factor is shown.

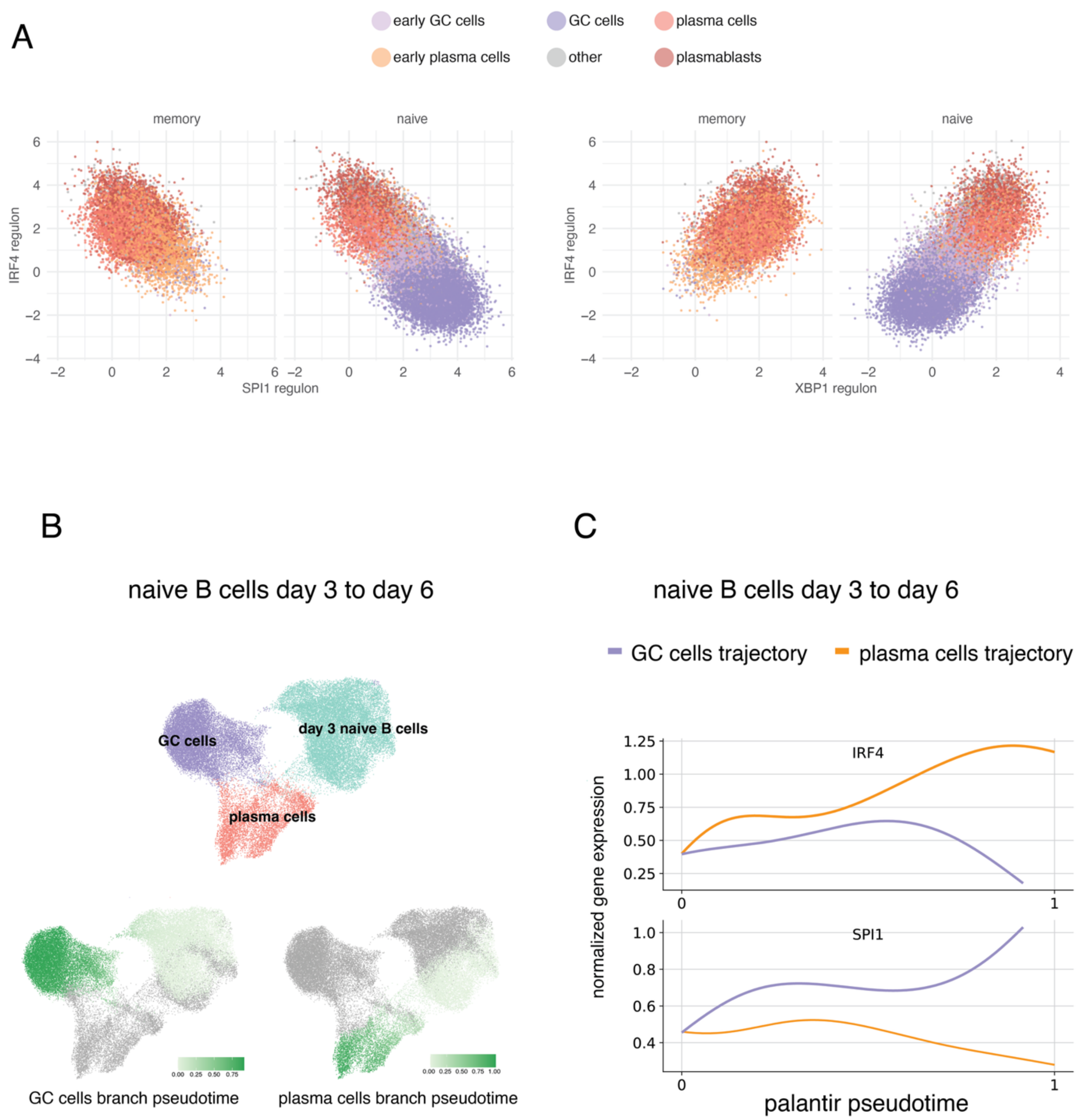

Appendix Figure S7 A) Dot plots showing the SPI1, IRF4 and XBP1 regulon scores in day six naive and memory B cells. Regulon annotations from Basso et al 2005, were scored on our cell model with the VIPER method from the decoupler python package. **B**) UMAP embeddings of naive B cells at day 3 and 6. Top UMAP shows the annotation. Bottom right and left UMAP represent the Palantir pseudotime across the two naive B cell pseudotime branches. Pseudotime is represented by a light green to dark green scale, with darker shades indicating higher pseudotime values **C**) Gene trends along the pseudotime for each naive B cell lineage.

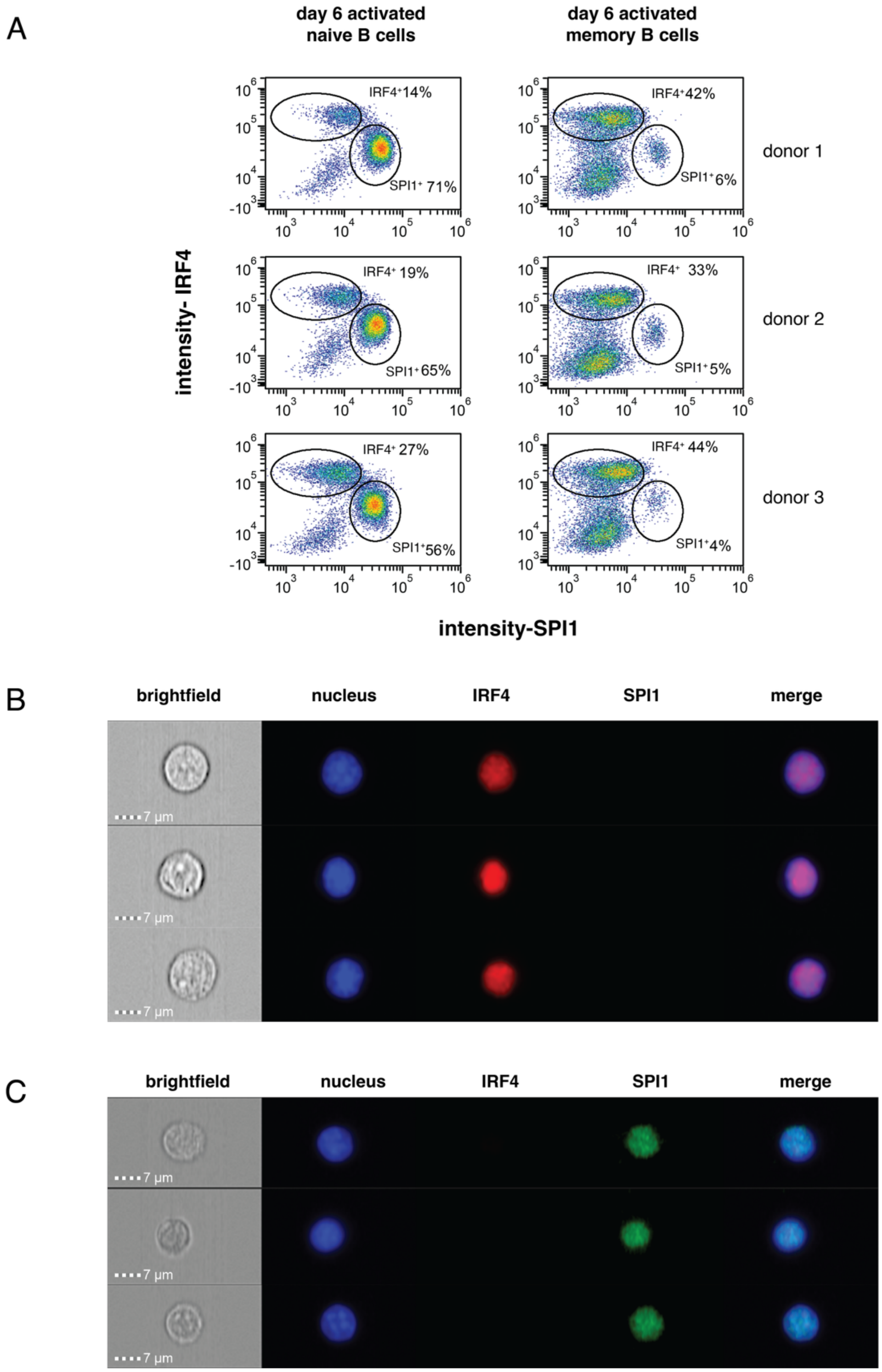

Appendix Figure S8 A) ImageStream intracellular staining of IRF4 and XBP1 in naive and memory B cells at day six after activation, n=3. **B-C**) Representative ImageStream images of activated naive and memory B cells at day six stained for nucleus (Draq5), IRF4 and SPI1. Images were acquired at 60× magnification.

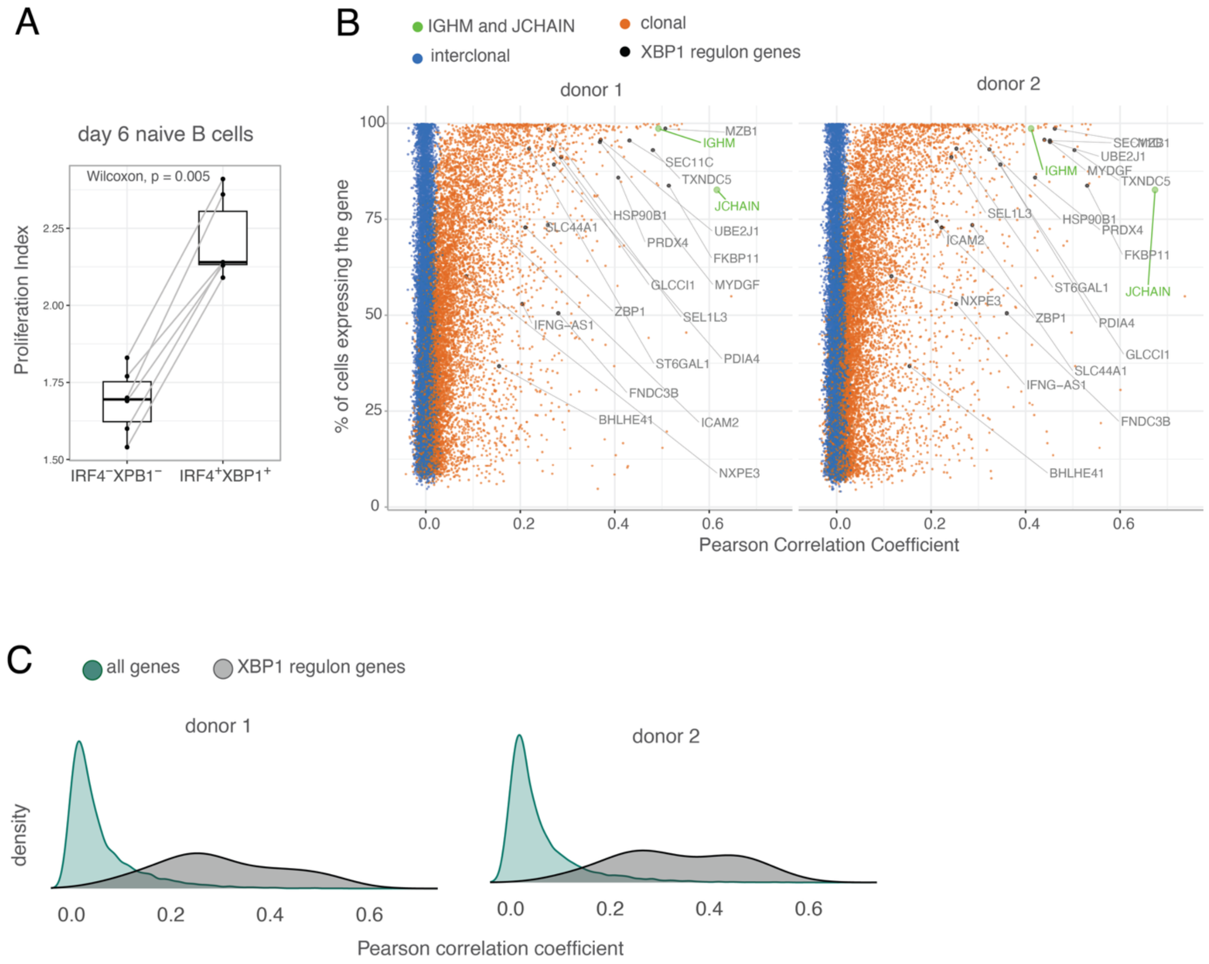

Appendix Figure S9 A) Proliferation index of IRF4^{-}XPB1^{-} IRF4^{+}XBP1^{+} naive B cells at day 6 post activation, n=6 biologically independent replicates. P-values were calculated with the Wilcoxon signed-ranked test. In the boxplots, the center line represents the median; boxes indicate the interquartile range (25th–75th percentiles); whiskers extend to the most extreme values within 1.5× the interquartile range. **B**) Pearson Correlation Coefficient of gene expression against percentage of cells expressing the gene. Each dot represents a gene. Orange and blue represent clonal and interclonal correlations. Genes belonging to the XBP1 regulon are highlighted in black. IGHM and JCHAIN are highlighted in green. n=2 biologically independent replicates. **C**) Density plot of clonal Pearson correlation coefficient for XBP1 regulon genes compared to all other genes. n=2.

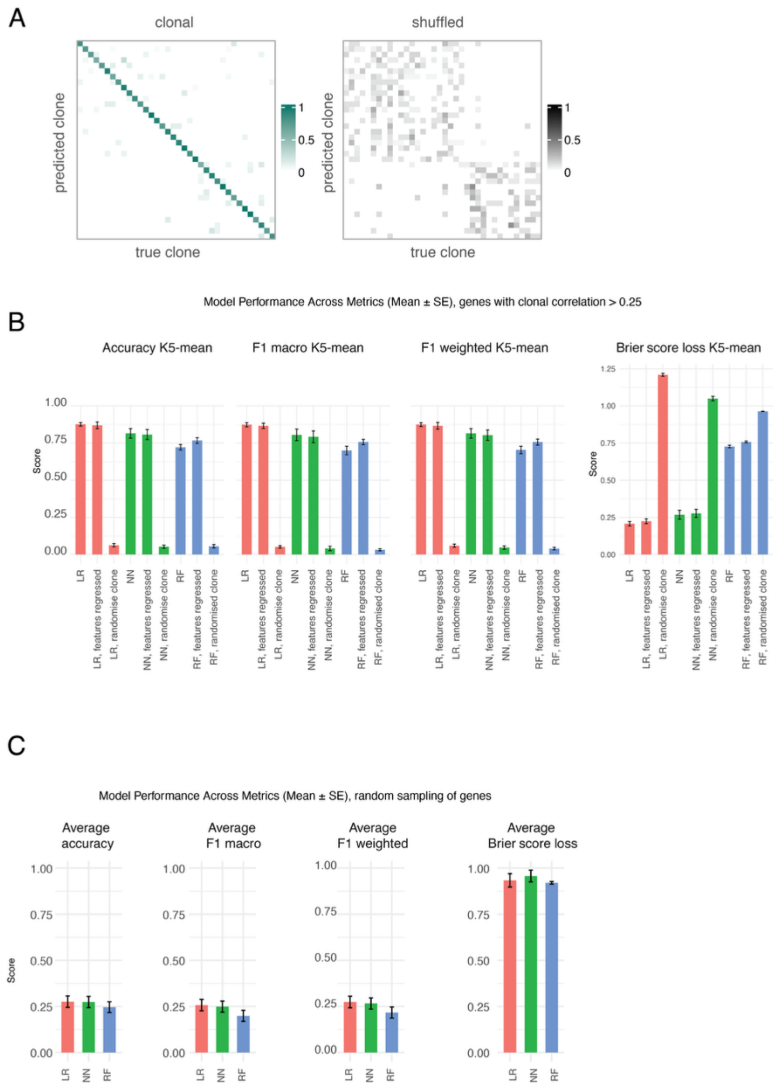

Appendix Figure S10 A) Confusion matrix illustrating the performance of the classifier. Rows represent the predicted clones and columns represent the true clonal assignment. The diagonal shows the proportion of correctly classified clones, while the off-diagonals indicate misclassification rate. Color intensity indicates the frequencies, with darker shades representing higher values. Green bars represent classification using real clonal assignments; grey bars represent a control where clonal groups were shuffled prior to training and validation. **B**) Bar plot showing the performance (accuracy, F1-score, Brier score loss) of machine learning models used to predict clonal identity based on gene expression profiles. Each bar represents the mean and standard error of the performance metric across cross-validation folds (K=5). Models compared include Logistic regression (LR), Neural Network (NN) and a Random Forest (RF) . All of the models used as input the same gene expression profiles from two day 6 post stimulation naive clonal datasets generated by us from two human healthy blood donors. Genes with a clonal correlation higher that Pearson correlation coefficient 0.25 were used as input (369 genes). “Feature regressed” indicates cell cycle and cell state specific effects were regressed out from gene expression scaled values “randomised clone” indicates that the clonal assignments were shuffled per donor before training. **C**) Model performance of different machine learning classifiers as in a, however the input features were 369 genes randomly sampled from genes with clonal correlation lower than 0.25 (”random genes”). Average and standard deviation of 100 different sampling runs are shown. a,b) Red, green and blue represent LR, NN, RF respectively.

A

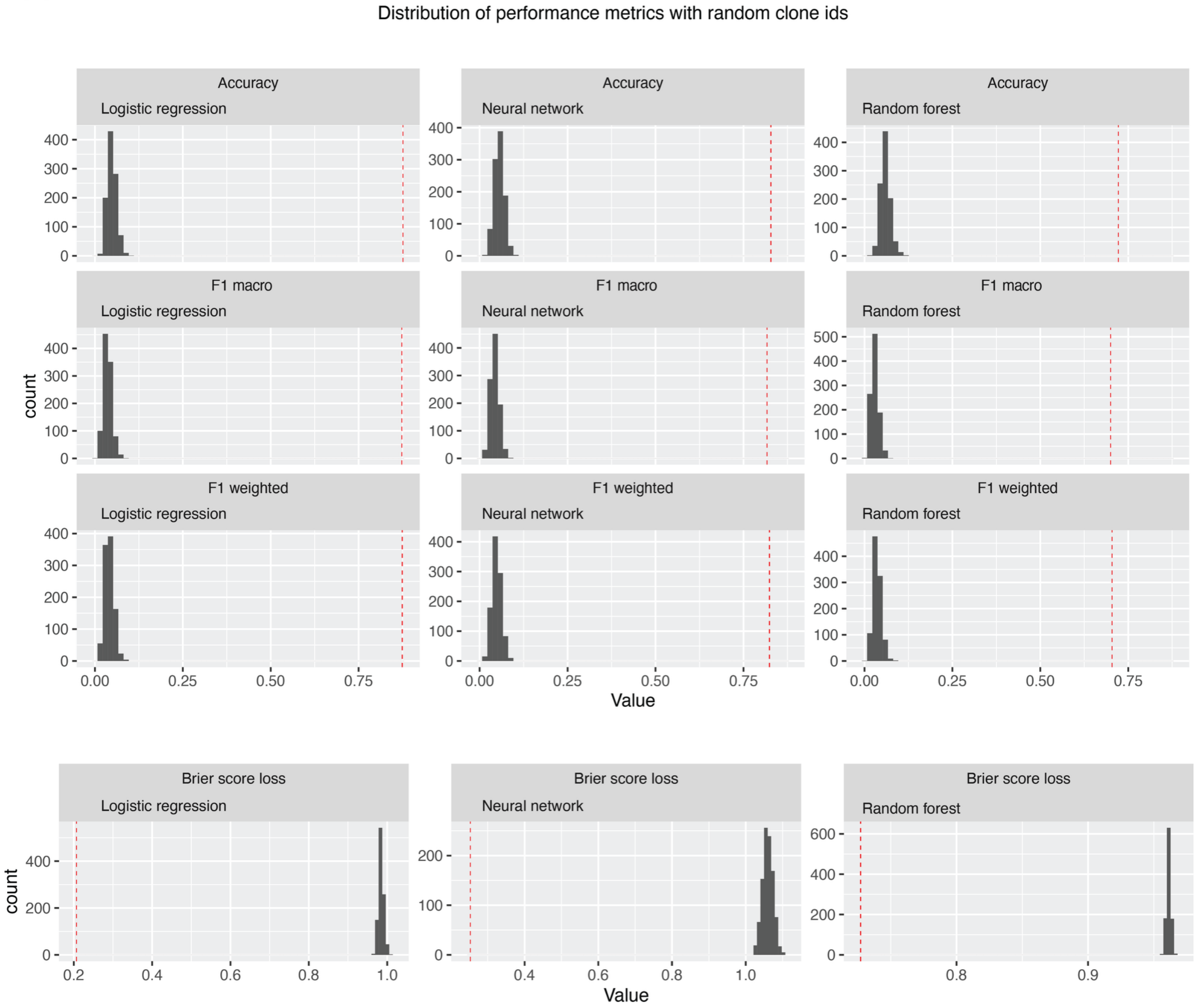

Appendix Figure S11 A) Distribution of accuracy metrics (accuracy, F1-score, Brier score loss) of three machine learning models used to predict clonal identity based on gene expression profiles, estimated with 1000 randomised clone ids. Random distribution was estimated by shuffling the clone ids per donor and running the classification 1000 times. Red lines represent the accuracy metric obtained on the non-randomised data. Models compared include logistic regression, random forest and neural network. All of the models used as input the same gene expression profiles from two day 6 post stimulation naive clonal datasets generated for this paper from two human healthy blood donors. Genes with a clonal correlation higher that Pearson correlation coefficient 0.25 were used as input (369 genes).

A

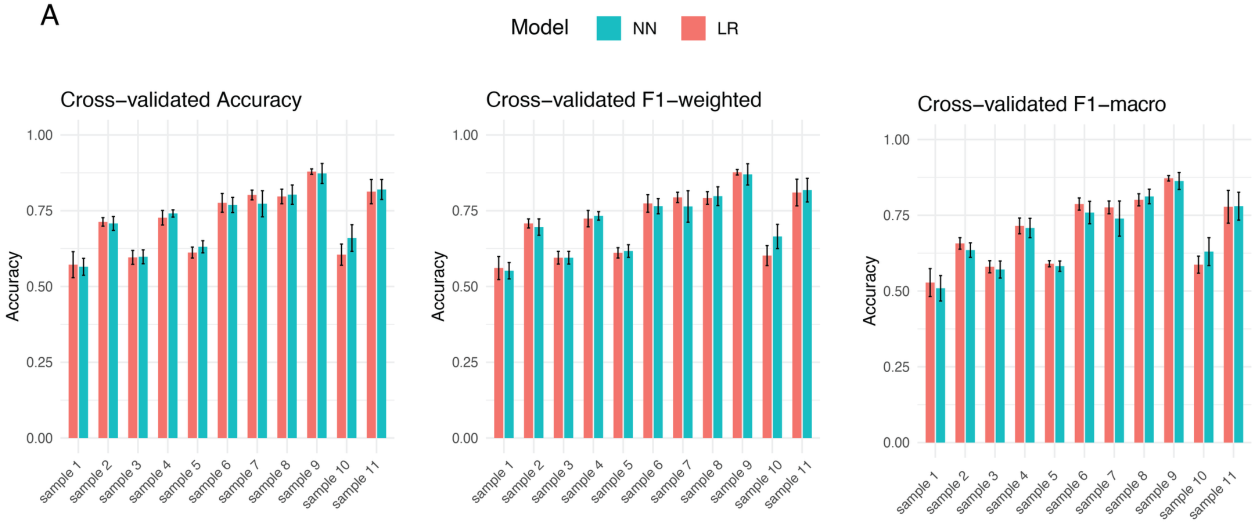

Appendix Figure S12 A) Bar plot showing the performance (accuracy, F1-score,) of various models used to predict clonal identity based on gene expression profiles on 11 mice B cell samples from (Agrafiotis et al. 2023). Each bar represents the mean and standard error of the performance metric across cross-validation folds (K=5). Models compared include logistic regression (LR) and a neural network (NN).

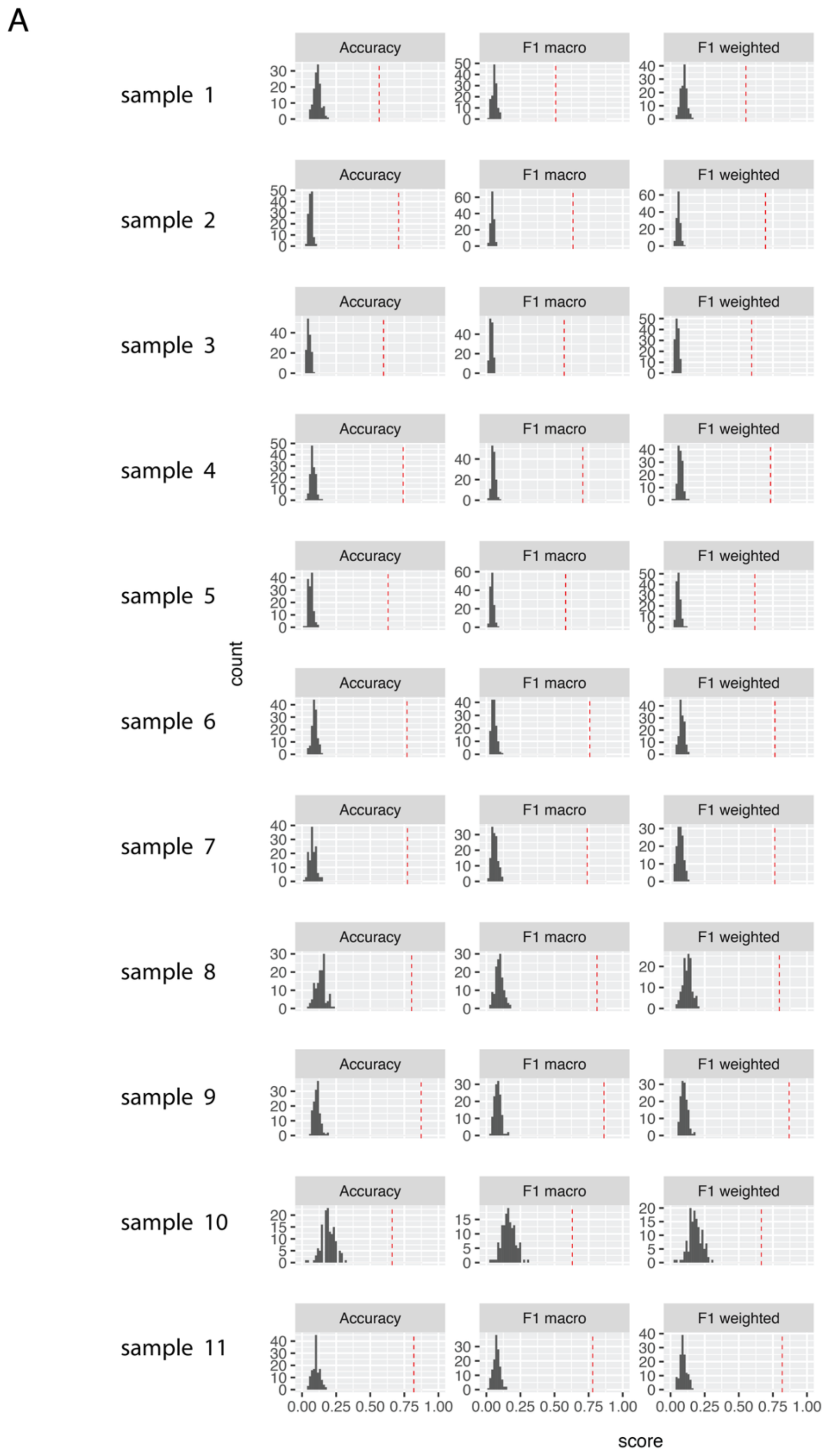

Appendix Figure S13 A) Distribution of accuracy metrics (accuracy, F1-score) of the neural network used to predict clonal identity based on gene expression profiles, estimated 100 times with randomised clone-ids within each mice B cell clonal dataset. Random distribution was estimated by shuffling the clone-ids within each mouse and running the classification 100 times. Red lines represent the accuracy metric obtained on the non-randomised data.

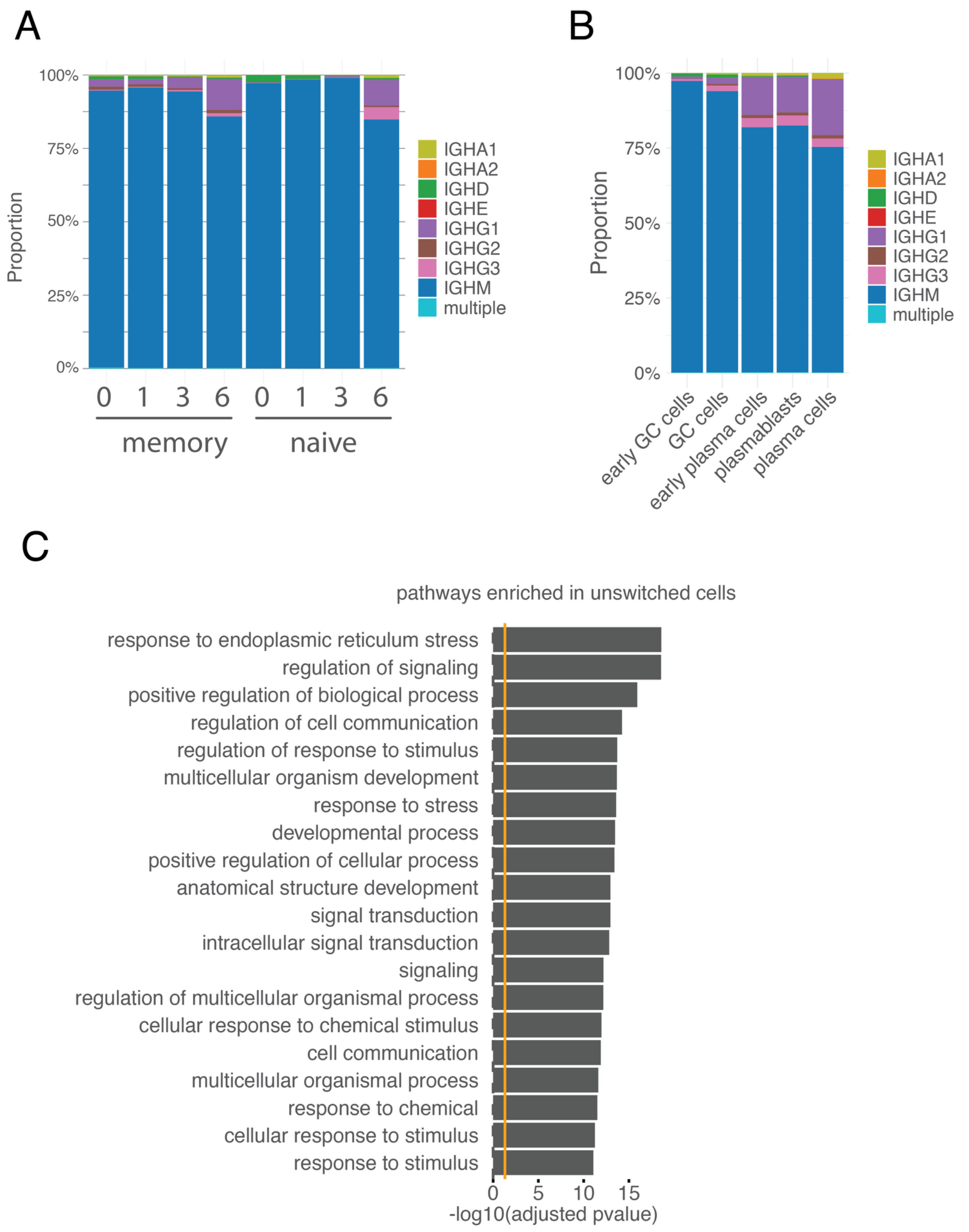

Appendix Figure S14 A) Proportion of antibody isotypes within each time point and cell type group. **B**) Proportion of antibody isotypes within each cell state. **C**) Top enriched reactome pathways in unswitched day 6 clonal naive B cells. Enrichment significance was assessed using a hypergeometric test.

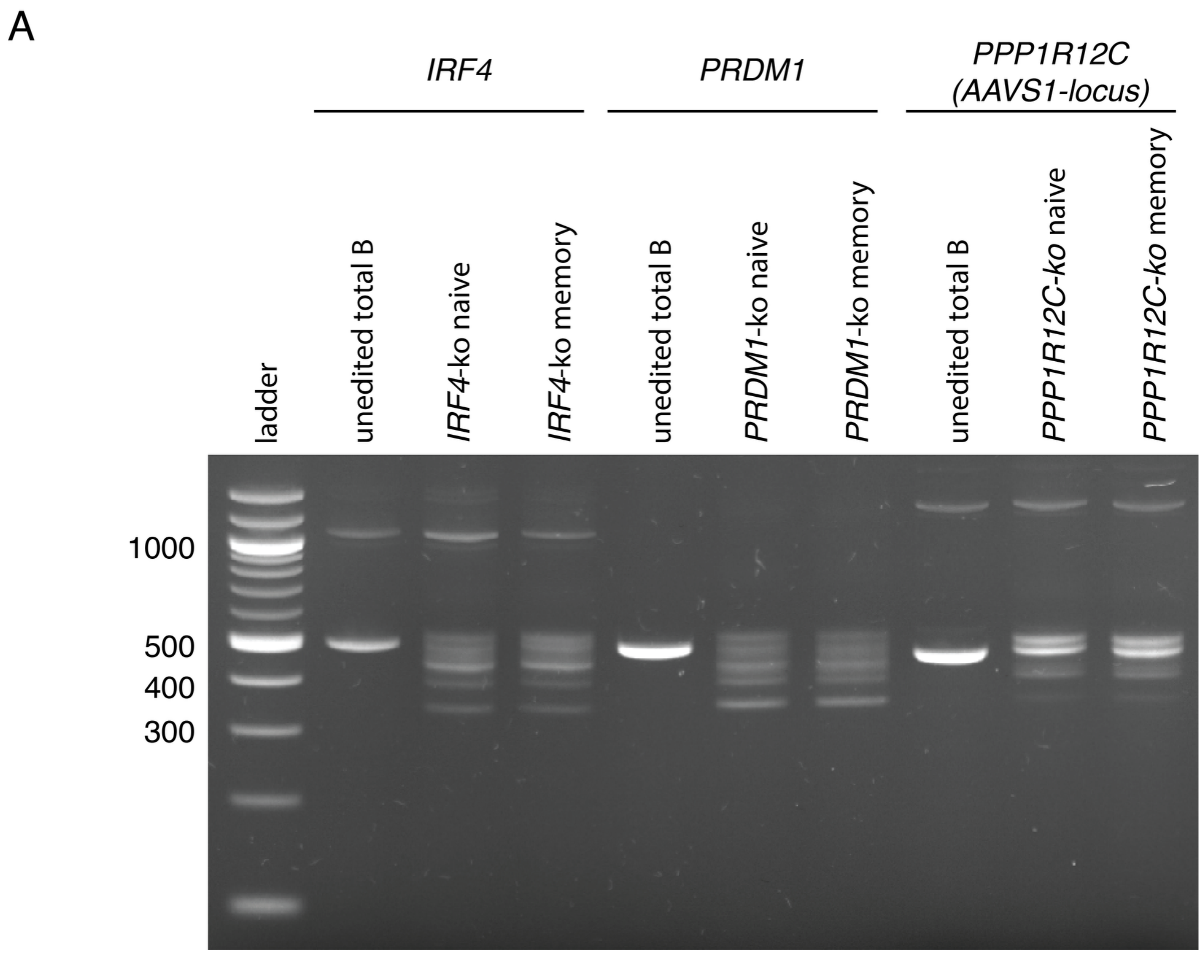

Appendix Figure S15 A) CRISPR-Cas9 genetic editing of IRF4, PRDM1 and negative control PPP1R12C. A 2% agarose gel image showing the PCR products of representative samples of unedited total B cells and gene-edited naïve and memory B cells.

A

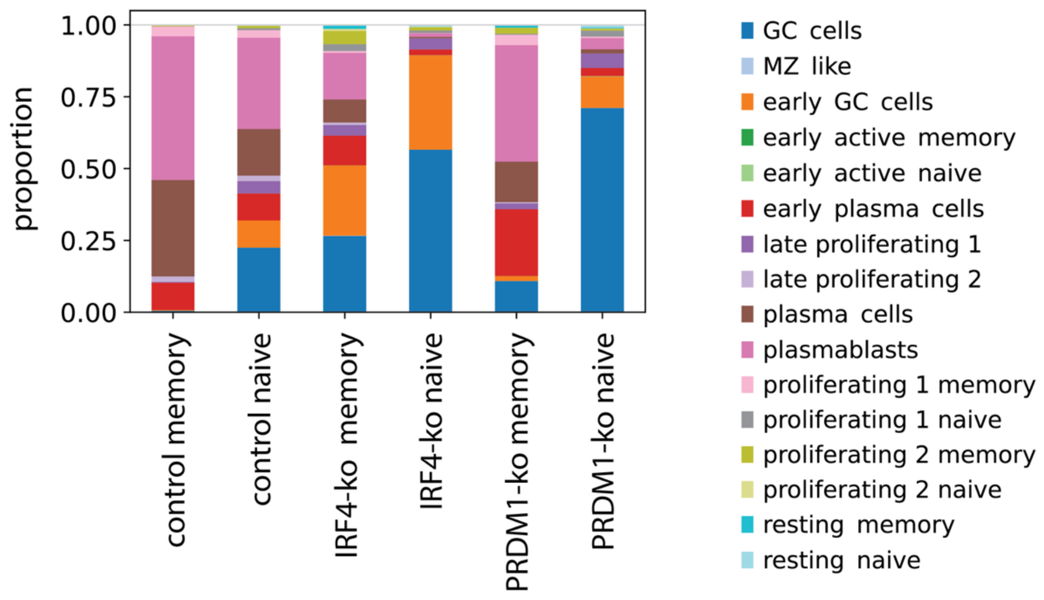

B

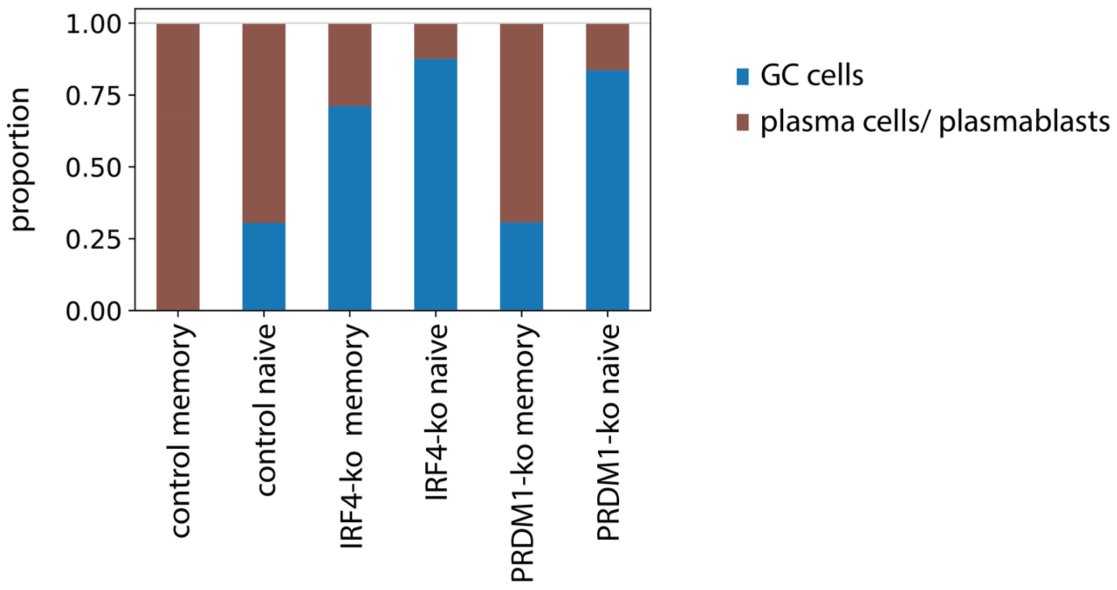

Appendix Figure S16 A) Barplot showing the fractions of cells from each sample as mapped by scANVI onto the reference dataset. Each bar represents the proportional composition of annotated B cell states within a given KO condition. **B**) Proportion of cells in different manually annotated cell states for naive and memory cells in day 6 KO conditions.

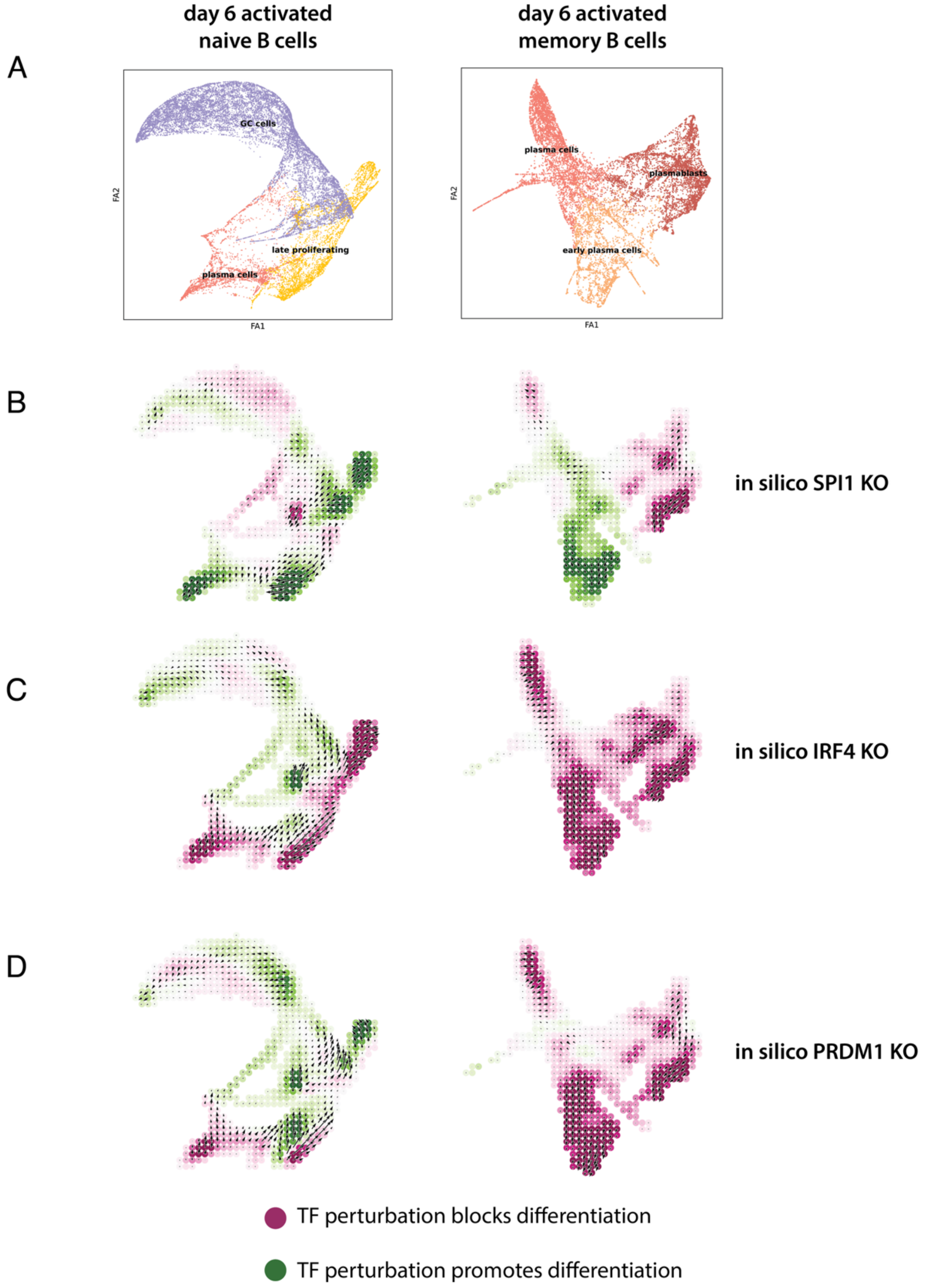

Appendix Figure S17 A) Force-directed graph embedding of naive and memory B cells at day six after activation. Color represents cell states. **B-D**) CellOracle KO simulation vector field with perturbation scores. Positive perturbation scores are represented in green and indicate that the transcription factor perturbation promotes differentiation. Negative perturbation scores are represented in purple and indicate that the transcription factor perturbation blocks differentiation. Arrows represent simulated cell state transitions following perturbation.

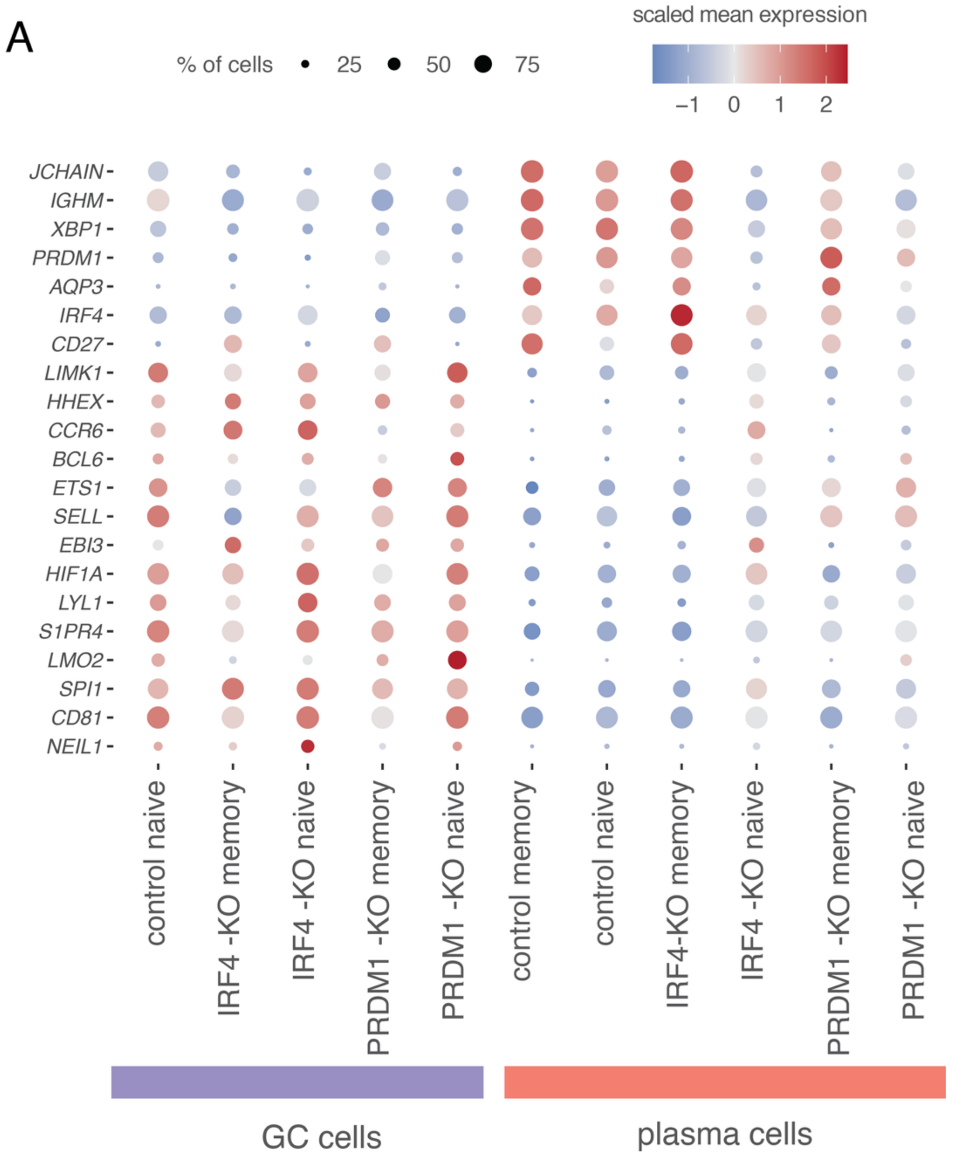

Appendix Figure S18 A) Dot plot of cell state specific genes. Scaled gene expression is represented on a blue-to-red gradient: blue indicates below-average expression, red indicates above-average expression. Dot size reflects the proportion of cells expressing each gene.
