# Supplementary figures and images for: Competing gene regulatory networks drive naive and memory B cell differentiation
Source: Mol Syst Biol. 2026 Apr 16;22(7):1097–117. doi: 10.1038/s44320-026-00207-8 (PMC13328623; doi:10.1038/s44320-026-00207-8)

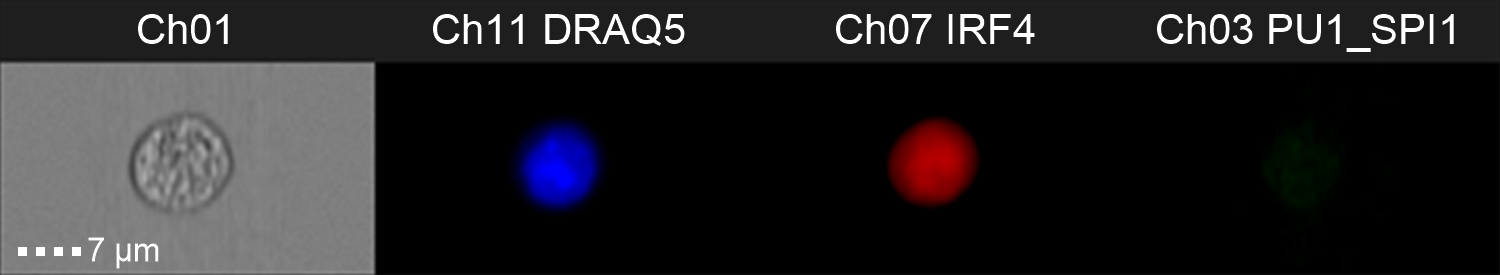

Supplement: Supplementary file 14 — Source data Fig. 2 [file 44320_2026_207_MOESM14_ESM.zip › SD figure 2/F/IRF4+SPI1-c.tif]

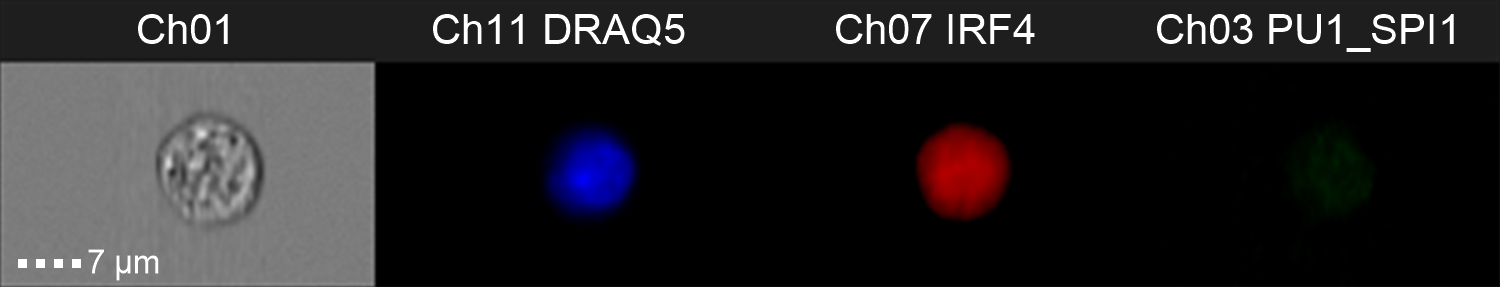

Supplement: Supplementary file 14 — Source data Fig. 2 [file 44320_2026_207_MOESM14_ESM.zip › SD figure 2/F/IRF4+SPI1-b.tif]

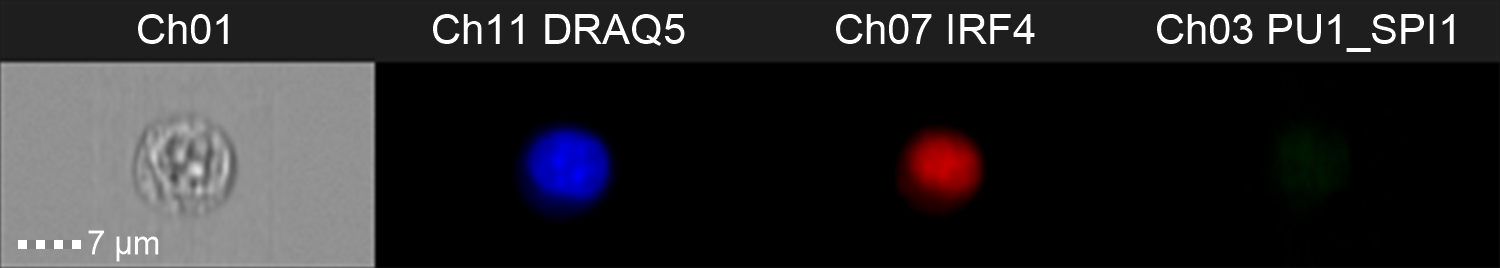

Supplement: Supplementary file 14 — Source data Fig. 2 [file 44320_2026_207_MOESM14_ESM.zip › SD figure 2/F/IRF4+SPI1-e.tif]

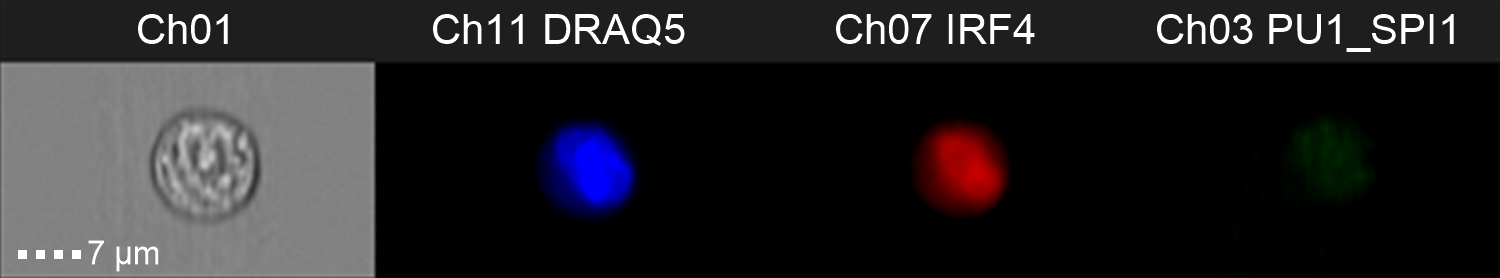

Supplement: Supplementary file 14 — Source data Fig. 2 [file 44320_2026_207_MOESM14_ESM.zip › SD figure 2/F/IRF4+SPI1-d.tif]

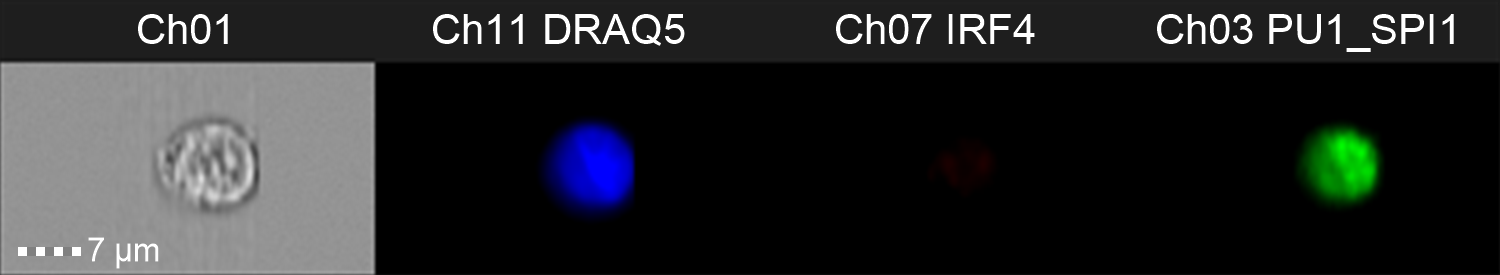

Supplement: Supplementary file 14 — Source data Fig. 2 [file 44320_2026_207_MOESM14_ESM.zip › SD figure 2/F/IRF4-SPI1+d.tif]

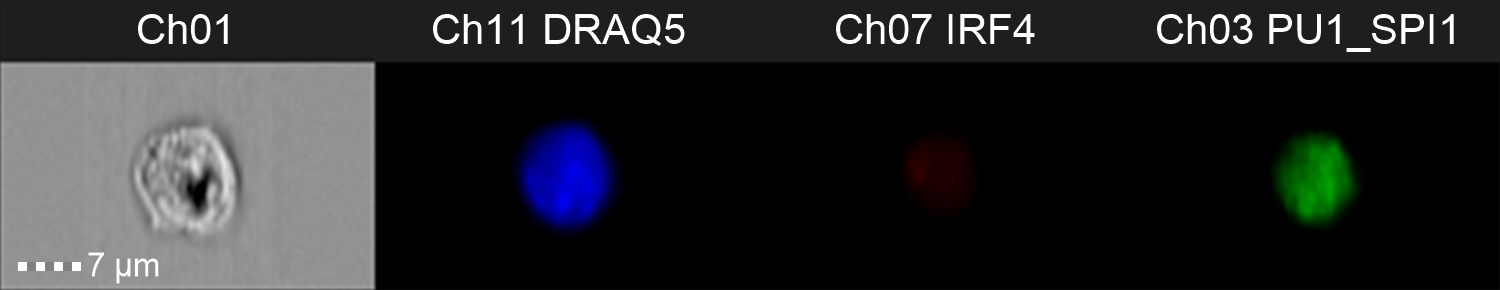

Supplement: Supplementary file 14 — Source data Fig. 2 [file 44320_2026_207_MOESM14_ESM.zip › SD figure 2/F/IRF4-SPI1+e.tif]

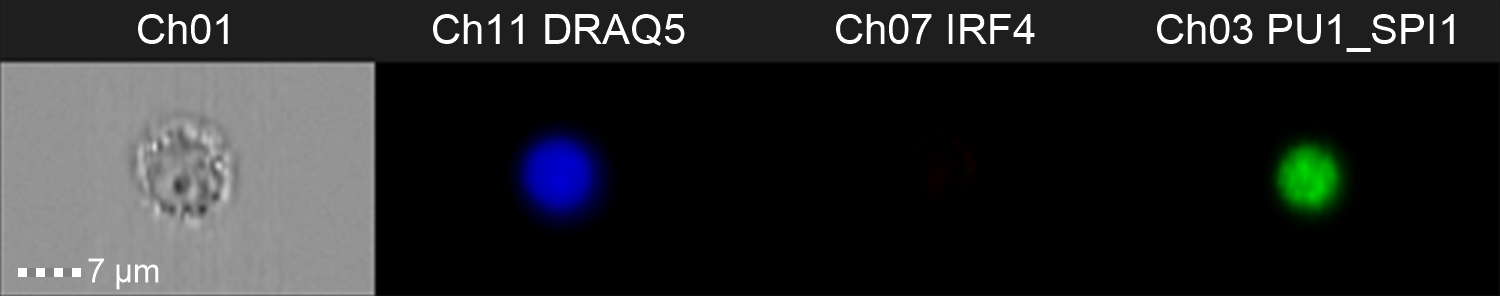

Supplement: Supplementary file 14 — Source data Fig. 2 [file 44320_2026_207_MOESM14_ESM.zip › SD figure 2/F/IRF4-SPI1+b.tif]

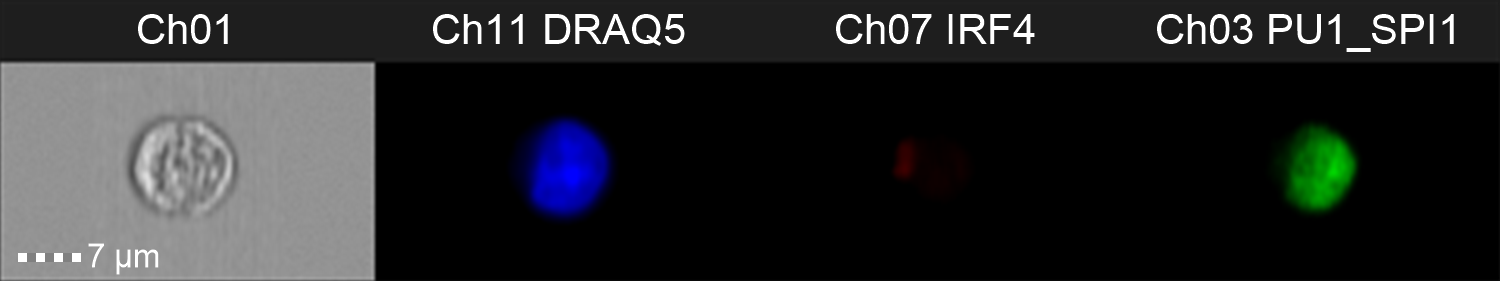

Supplement: Supplementary file 14 — Source data Fig. 2 [file 44320_2026_207_MOESM14_ESM.zip › SD figure 2/F/IRF4-SPI1+c.tif]

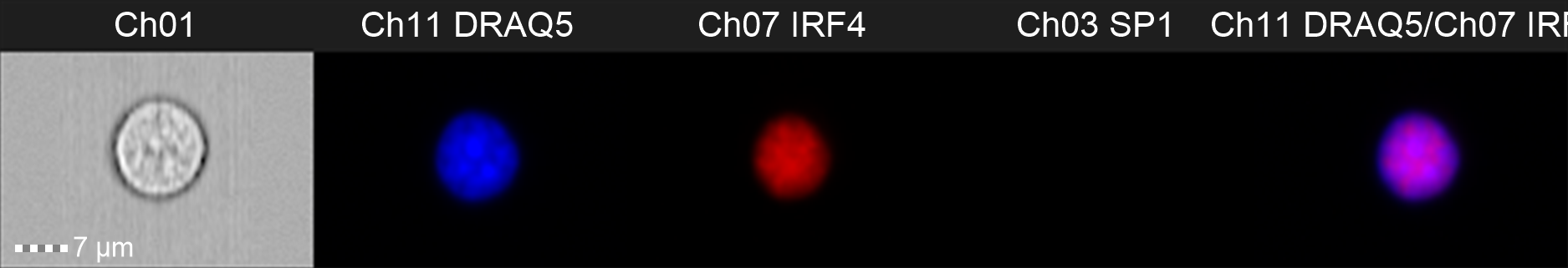

Supplement: Supplementary file 16 — Appendix Figure S8 Source Data [file 44320_2026_207_MOESM16_ESM.zip › SD Appendix S8/R478_memory_image2.tif]

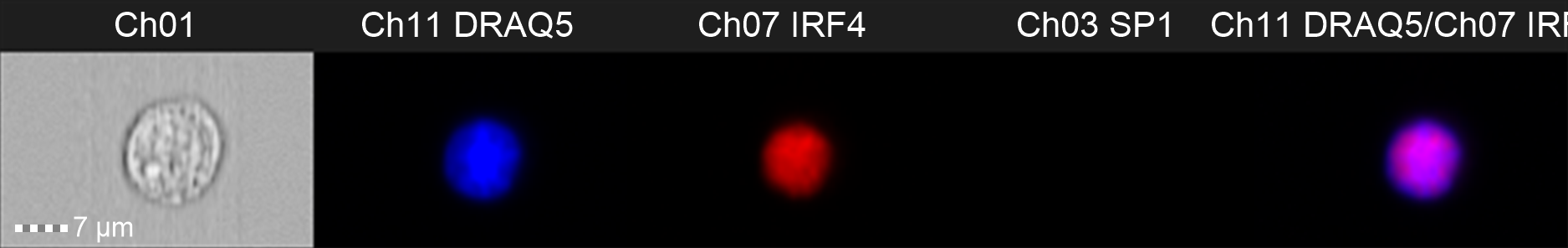

Supplement: Supplementary file 16 — Appendix Figure S8 Source Data [file 44320_2026_207_MOESM16_ESM.zip › SD Appendix S8/R478_memory_image3.tif]

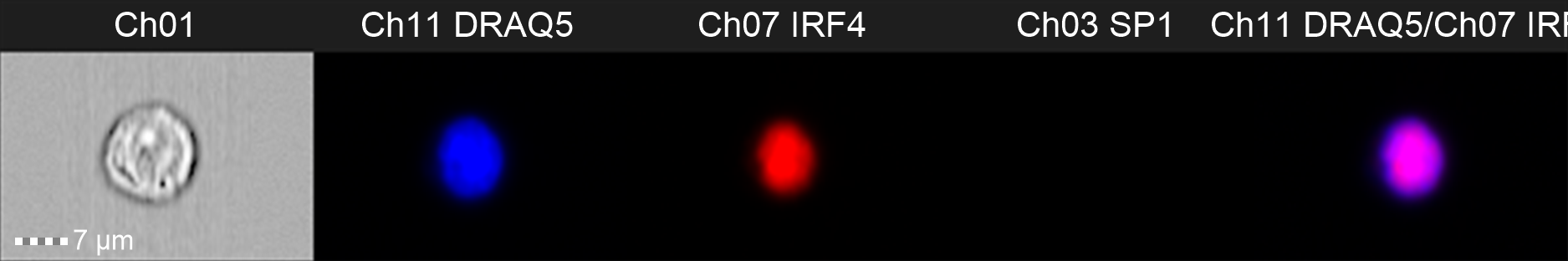

Supplement: Supplementary file 16 — Appendix Figure S8 Source Data [file 44320_2026_207_MOESM16_ESM.zip › SD Appendix S8/R478_memory_image4.tif]

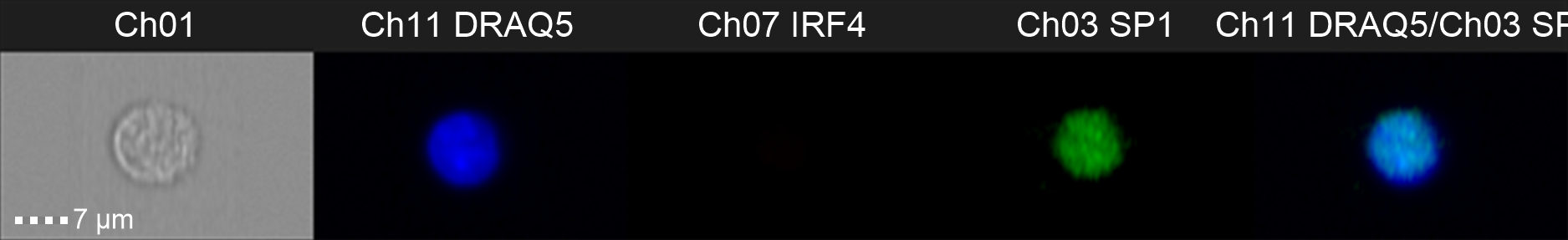

Supplement: Supplementary file 16 — Appendix Figure S8 Source Data [file 44320_2026_207_MOESM16_ESM.zip › SD Appendix S8/R478_naive_image1.tif]

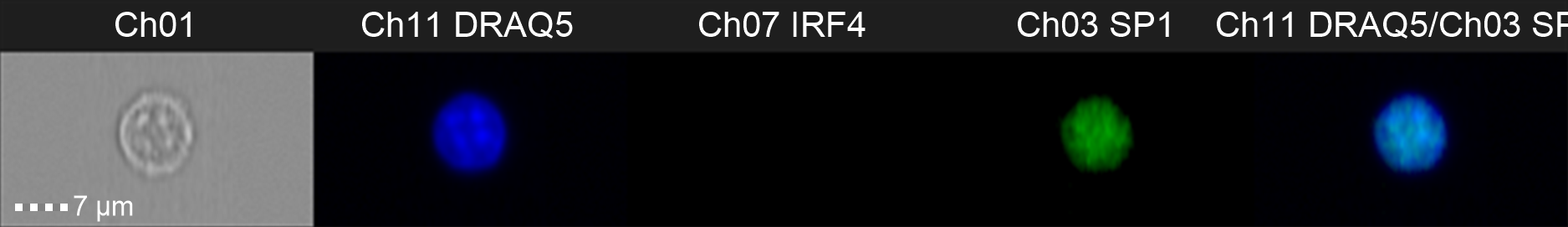

Supplement: Supplementary file 16 — Appendix Figure S8 Source Data [file 44320_2026_207_MOESM16_ESM.zip › SD Appendix S8/R478_naive_image2.tif]

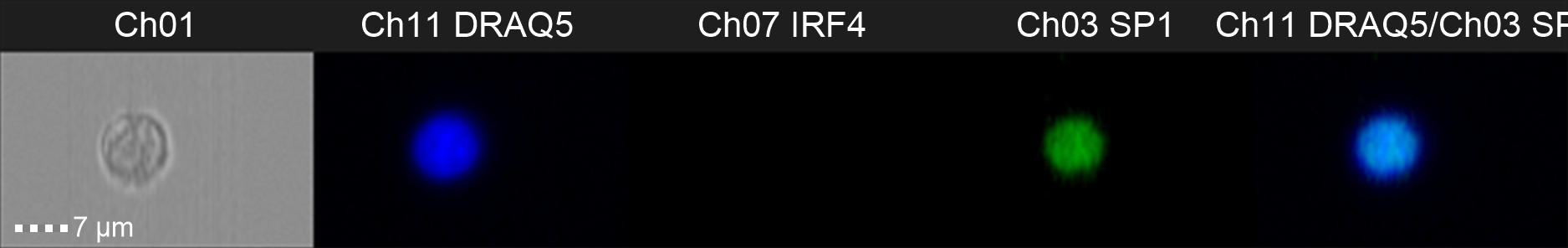

Supplement: Supplementary file 16 — Appendix Figure S8 Source Data [file 44320_2026_207_MOESM16_ESM.zip › SD Appendix S8/R478_naive_image3.tif]
