# Supplementary material for: Competing gene regulatory networks drive naive and memory B cell differentiation
Source: Mol Syst Biol. 2026 Apr 16;22(7):1097–117. doi: 10.1038/s44320-026-00207-8 (PMC13328623; doi:10.1038/s44320-026-00207-8)
Supplement: Supplementary file 1 — Peer Review File [file 44320_2026_207_MOESM1_ESM.pdf]

molecular systems biology

Competing gene regulatory networks drive naive and memory B cell differentiation

Blagoje Soskic, Pietro Demela, Laura Esposito, Pietro Marchesan, Davide Bolognini, Edoardo Giacopuzzi, Eugenia Ricciardelli, Paolo Ferrari, Silvia Bombelli, Clelia Peano, Daniele Prati, Luca Valenti, and Leonardo Nossa

Corresponding author(s): Blagoje Soskic (blagoje.soskic@fht.org)

Review Timeline:

Submission Date:	31st Jul 25
Editorial Decision:	12th Sep 25
Revision Received:	19th Dec 25
Editorial Decision:	28th Jan 26
Revision Received:	26th Feb 26
Accepted:	23rd Mar 26

Editor: Poonam Bheda

Transaction Report:

(Note: With the exception of the correction of typographical or spelling errors that could be a source of ambiguity, letters and reports are not edited. Depending on transfer agreements, referee reports obtained elsewhere may or may not be included in this compilation. Referee reports are anonymous unless the Referee chooses to sign their reports.)

12th Sep 2025

Manuscript Number: MSB-2025-13265-T
Title: Competing gene regulatory networks drive naive and memory B cell differentiation

Dear Dr. Soskic,

Thank you for the submission of your manuscript to Molecular Systems Biology. We have now received feedback from the three reviewers who agreed to evaluate your manuscript. As you will see from the reports below, the referees acknowledge the interest of the study and are overall supportive of your work; however they also comment on multiple aspects of the manuscript that should be strengthened in a revision.

I think that the recommendations of the reviewers are rather clear and I therefore do not see the need to repeat the comments listed below. All issues raised would need to be satisfactorily addressed. Please let me know in case you would like to discuss in further detail any of the any of the reviewer comments or your proposed revisions, I would be happy to schedule a call.

When submitting your revised manuscript, please carefully review the instructions that follow below. We perform an initial quality control of all revised manuscripts before re-review; failure to include requested items will delay the evaluation of your revision.

We require:

1) A .docx formatted version of the manuscript text (including legends for main figures, EV figures and tables). Please make sure that the changes are highlighted to be clearly visible. Alternatively you may choose to submit your manuscript as a LaTeX file.

2) Individual production quality figure files as .eps, .tif, .jpg (one file per figure). For guidance, download the 'Figure Guide PDF' (https://www.embopress.org/page/journal/17574684/authorguide#figureformat).

3) At EMBO Press we ask authors to provide source data for the main figures. Our source data coordinator will contact you to discuss which figure panels we would need source data for and will also provide you with helpful tips on how to upload and organize the files.

4) A .docx formatted letter INCLUDING the reviewers' reports and your detailed point-by-point responses to their comments. As part of the EMBO Press transparent editorial process, the point-by-point response is part of the Peer Review File (PRF), which will be published alongside your paper.

5) A complete author checklist, which you can download from our author guidelines (https://www.embopress.org/page/journal/17574684/authorguide#submissionofrevisions). Please insert information in the checklist that is also reflected in the manuscript. The completed author checklist will also be part of the PRF.

6) Please note that all corresponding authors are required to supply an ORCID ID for their name upon submission of a revised manuscript.

7) It is mandatory to include a 'Data Availability' section after the Materials and Methods. Before submitting your revision, primary datasets produced in this study need to be deposited in an appropriate public database, and the accession numbers and database listed under 'Data Availability'. Please remember to provide a reviewer password if the datasets are not yet public (see https://www.embopress.org/page/journal/17574684/authorguide#dataavailability).

In case you have no data that requires deposition in a public database, please state so in this section as follows: "This study includes no data deposited in external repositories". Note that the Data Availability Section is restricted to new primary data that are part of this study.

8) All Materials and Methods need to be described in the main text using our 'Structured Methods' format, which is required for all research articles. According to this format, the Methods section includes a Reagents and Tools Table (listing key reagents, experimental models, software and relevant equipment and including their sources and relevant identifiers) followed by a Methods and Protocols section describing the methods using a step-by-step protocol format. The aim is to facilitate adoption of the methodologies across labs. Please upload the Reagents and Tools table as a separate document when submitting your revised manuscript. More information on how to adhere to this format as well as a downloadable template (.docx) for the Reagents and Tools Table can be found in our author guidelines:
https://www.embopress.org/page/journal/17444292/authorguide#structuredmethods

An example of a Method paper with Structured Methods can be found here:

https://www.embopress.org/doi/10.15252/msb.20178071.

9) For data quantification: please specify the name of the statistical test used to generate error bars and p-values, the number (n) of independent experiments (specify technical or biological replicates) underlying each data point and the test used to calculate p-values in each figure legend. The figure legends should contain a basic description of n, p-values and the test applied. Graphs must include a description of the bars and the error bars (s.d., s.e.m.). Please provide exact p-values (in either the figure or figure legend).

10) Our journal encourages inclusion of *data citations in the reference list* to directly cite datasets that were re-used and obtained from public databases. Data citations in the article text are distinct from normal bibliographical citations and should directly link to the database records from which the data can be accessed. In the main text, data citations are formatted as follows: "Data ref: Smith et al, 2001" or "Data ref: NCBI Sequence Read Archive PRJNA342805, 2017". In the Reference list, data citations must be labeled with "[DATASET]". A data reference must provide the database name, accession number/identifiers and a resolvable link to the landing page from which the data can be accessed at the end of the reference. Further instructions are available at .

11) We replaced Supplementary Information with Expanded View (EV) Figures and Tables that are collapsible/expandable online. EV Figures should be cited as 'Figure EV1, Figure EV2" etc... in the text and their respective legends should be included in the main text after the legends of regular figures.
- For the figures that you do NOT wish to display as Expanded View figures, they should be bundled together with their legends in a single PDF file called *Appendix*, which should start with a short Table of Content. Appendix figures should be referred to in the main text as: "Appendix Figure S1, Appendix Figure S2" etc.
- Additional Tables/Datasets should be labeled and referred to as Table EV1, Dataset EV1, etc. Legends should be provided in a separate tab in case of .xls files. Alternatively, the legend can be supplied as a separate text file (README) and zipped together with the Table/Dataset file.
See detailed instructions here:
https://www.embopress.org/page/journal/17574684/authorguide#expandedview

12) Author contributions: CRediT has replaced the traditional author contributions section because it offers a systematic machine-readable author contributions format that allows for more effective research assessment. Please remove the Authors Contributions from the manuscript and use the free text boxes beneath each contributing author's name in our system to add specific details on the author's contribution. More information is available in our guide to authors.

13) Disclosure statement and competing interests: We updated our journal's competing interests policy in January 2022 and request authors to consider both actual and perceived competing interests. Please review the policy https://www.embopress.org/competing-interests and update your competing interests if necessary.

14) Every published paper now includes a 'Synopsis' to further enhance discoverability. Synopses are displayed on the journal webpage and are freely accessible to all readers. They include a short stand first (maximum of 300 characters, including space) as well as 2-5 one-sentences bullet points that summarizes the paper. Please write the bullet points to summarize the key NEW findings. They should be designed to be complementary to the abstract - i.e. not repeat the same text. We encourage inclusion of key acronyms and quantitative information (maximum of 30 words / bullet point). Please use the passive voice. Please attach these in a separate file or send them by email, we will incorporate them accordingly.

Please also suggest a striking image or visual abstract to illustrate your article as a PNG file 550 px wide x 300-600 px high.

Please note that these would be the final versions and changes during proofing are usually not allowed.

15) As part of the EMBO Publications transparent editorial process initiative (see our policy here: https://www.embopress.org/transparent-process#Review_Process), Molecular Systems Biology will publish online a Peer Review File (PRF) to accompany accepted manuscripts.
In the event of acceptance, this file will be published in conjunction with your paper and will include the anonymous referee reports, your point-by-point response and all pertinent correspondence relating to the manuscript. Let us know whether you agree with the publication of the PRF and as here, if you want to remove or not any figures from it prior to publication.
Please note that the Author checklist will be published at the end of the PRF.

Molecular Systems Biology has a "scooping protection" policy, whereby similar findings that are published by others during review or revision are not a criterion for rejection. Should you decide to submit a revised version, I do ask that you get in touch after three months if you have not completed it, to update us on the status.

Yours sincerely,

Poonam Bheda, PhD

Scientific Editor
Molecular Systems Biology

Reviewer #1:

This study aims to systematically define the transcriptional network governing the cell fate choice between MBCs and naïve B cells in ex vivo cultures. The authors performed integrative analysis of transcriptional profiles using scRNA-seq and scBCR-seq. The key finding is that distinct CGNs regulate the differentiation fate of MBCs and naïve B cells. While these findings align with existing literature, the study provides novel evidence to strengthen conclusions in this field. Overall, the work is compelling, and the manuscript is well-written. However, I have the following concerns:
1. Fig.5a shows that IRF4 KO increased the frequencies of GC B cells. Optimal IRF4 has been reported to be important for BCL6 induction and GC B cell generation (Ochiai et al immunity 2013). It is suggested to carefully examine the proportion of each subset among total B cells upon IRF4 KO.
2. Bach2 is an import TF that restrains MBC and naive B to differentiate into PCs (Hu et al. cell reports 2022; Ochiai et al EMBO 2024). It is interesting to provide the detailed analysis of Bach2 (Fig2a and Fig5)

Reviewer #2:

In this paper, the authors presented an investigation of gene regulatory networks controlling naive and memory B cell differentiation using integrated single-cell RNA-sequencing and BCR-sequencing approaches. This work provides valuable insights into the distinct differentiation trajectories of these cell populations and includes functional validation through CRISPR perturbations. However, several aspects require clarification and improvement before publication. They are listed below:
1. CRISPR efficiency through PCR and flow cytometry in Methods section, quantitative data on knockout efficiency are not presented in the results. Including specific knockout percentages and representative validation data (e.g., gel images, protein expression levels) in the supplementary materials would strengthen the reliability of the perturbation experiments and enhance reproducibility.
2. The stimulation cocktail used for B cell activation (including CD40L, IL-2, IL-21, etc.) is described, but it would be helpful to include more justification for the chosen concentrations and their relevance to physiological conditions. A reference to similar studies or a comparison to different concentrations could strengthen this aspect.
3. Although some quality control steps for the scRNA-seq data are mentioned, such as cell filtering and doublet removal, the specific quality control criteria and methods are not detailed. It is recommended to provide a more thorough description of the quality control process in Results section or supplementary materials, including the specific criteria for cell selection (such as gene count, mitochondrial gene proportion, etc.), thresholds for doublet removal, and the methods used for data normalization. Additionally, quality control plots or relevant numerical data should be included to enhance the reproducibility and transparency of the data.
4. The neural network model predicting sister cell relationships based on transcriptomic data is interesting. However, the manuscript would benefit from additional validation of this prediction. Can the authors provide an independent dataset or validation experiment that confirms the prediction model's accuracy in predicting sister cells?
5. The authors have performed several important validation experiments (e.g., ImageStream analysis of IRF4/SPI1 localization) that support their main conclusions. However, the presentation of these validation data could be optimized, potentially by promoting some supplementary validation results to main figures to better support the central claims.
6. Some conclusions appear to interpret correlative findings as causal relationships. The manuscript would benefit from more cautious language when describing regulatory relationships, particularly distinguishing between observations of gene expression correlations and direct evidence of regulatory control.

Despite these concerns, the integration of transcriptomic and clonal tracking data, combined with functional perturbations, provides valuable mechanistic insights.

Reviewer #3:

Summary
Establishing a rigorous map of the gene-regulatory programs that govern human B-cell fate is of broad interest for immunology and systems biology. The manuscript "Competing gene regulatory networks drive naive and memory B-cell differentiation" profiles naive and memory B cells upon activation (10x 5′ scRNA-seq coupled with scBCR-seq), infers TF activities and regulons, and combines this with CRISPR perturbations of IRF4 and PRDM1. The authors report a naive bifurcation into germinal center-like (GC-like) and plasma cell (PC) states versus a memory bias toward PCs, anticorrelated IRF4-SPI1 activity,

germline transcript (GLT) enrichment in GC-like cells, and clonal transcriptomic heritability supported by a neural-network classifier.

The study is potentially important. However, there are concerns regarding the depth and rigor of key analyses and validations. Detailed comments follow.

Major concerns:

1. Absence of trajectory/fate modeling
The bifurcation claim would be much stronger with dynamical evidence. Please compute RNA velocity for naïve and memory B-cell populations separately (days 0/1/3/6) and use CellRank to estimate absorption probabilities toward PC vs GC-like endpoints. Map IRF4 and SPI1 dynamics along pseudotime and quantify early (day 1/3) fate biases to further support the manuscript's claim of anticorrelated IRF4-SPI1 activity.

2. Lack of independent GRN confirmation
SCENIC on SEAcells (30 runs; 80% regulon retention) is solid, but TF-activity claims should be corroborated with an independent framework. Please add DoRothEA + VIPER and/or Pando and provide concordance metrics (rank correlations, top-k overlaps, permutation p-values), highlighting agreements/disagreements for IRF4/SPI1/XBP1.

3. Limited KO validation and causal modeling
The KO data are interesting, but the link to network perturbation is not established. Please perform in silico perturbations with CellOracle to complement the KOs: single-gene KOs for IRF4, PRDM1, SPI1, and a double KO (IRF4+PRDM1) to test whether PRDM1 loss adds beyond IRF4 loss. Compare predicted state probabilities with your observed KO data. This will directly support the network-causality narrative.

4. Insufficient justification and benchmarking of the sister-cell neural network
You use a 4-layer FFNN with dropout 0.4 and features selected by interclonal correlation ($r>0.25$), but there is no justification of this architecture nor reporting of full performance metrics. Please benchmark against baselines (kNN on PCs, logistic regression, random forest), report accuracy/AUROC/F1 and calibration, and include an ablation regressing out cell-cycle and PC/GC markers to show the model is not simply recapitulating cell state.

Minor concern

1. Missing methodological specifics to ensure reproducibility
a. SCENIC - Add SEAcells parameters (number cells per metacell, k, seed), motif database versions, GRN stability thresholds (80% regulon retention; 70% gene retention) and rationale; exact AUCell settings and RSS calculation.
b. sciCSR - It is cited in Results but absent in Methods. Please add version, reference, alignment/remapping parameters, GLT definitions, minimum read thresholds, and chemistry awareness (10x 5′ v2).
c. QC transparency - Report numeric QC cutoffs (minimum genes/UMIs, mitochondrial %, doublet score thresholds) per time point and donor; give post-QC cell counts per group.

Response to Reviewers

We thank all reviewers for their valuable comments and constructive feedback. Their efforts are greatly appreciated, and we believe that our revisions have strengthened the paper. We address each comment point-by-point below.

Reviewer #1:

This study aims to systematically define the transcriptional network governing the cell fate choice between MBCs and naïve B cells in ex vivo cultures. The authors performed integrative analysis of transcriptional profiles using scRNA-seq and scBCR-seq. The key finding is that distinct CGNs regulate the differentiation fate of MBCs and naïve B cells. While these findings align with existing literature, the study provides novel evidence to strengthen conclusions in this field. Overall, the work is compelling, and the manuscript is well-written. However, I have the following concerns:

We thank the reviewer for their positive assessment of the manuscript and their recognition of its clarity and overall quality.

1. Fig.5a shows that IRF4 KO increased the frequencies of GC B cells. Optimal IRF4 has been reported to be important for BCL6 induction and GC B cell generation (Ochiai et al immunity 2013). It is suggested to carefully examine the proportion of each subset among total B cells upon IRF4 KO.

We thank the reviewer for this comment. To further characterise the B cells in the KO, we applied scANVI (Xu et al. 2021), a semi-supervised model that leverages labeled data from the time course to learn a cell-type classifier in the latent space and subsequently predict cell types in the KO dataset. This confirmed that these cells correspond to GC and early GC B cells (**Fig. R1** and **Fig EV16**). For example, following activation of naive B cells 30% of cells in the control and 90% of cells in the IRF4 KO were annotated as GC B cells by scANVI. Finally, we fully agree with the reviewer that IRF4 is required for proper GC B cell differentiation and that although these cells map to WT GCs, the observed cells represent an impaired GCs. We have discussed this point in the Results section, where we now describe this as follows:

"Next, we investigated whether the emergence of SPI1-regulon high cells in activated memory B cells following IRF4 and PRDM1 KOs resembled the GC cells arising from natural naive B cell differentiation. To investigate this, we compared GC cells from control naive cells to those arising in memory cells following IRF4 and PRDM1 KOs. Strikingly, GC cells from memory B cells lacking PRDM1 were transcriptionally indistinguishable from those arising from naive cells in control condition (**Fig. 5F**). This demonstrated that PRDM1 is only essential for plasma cell generation but dispensable for GC cell development. In contrast, GC cells emerging in memory cells following IRF4 KO displayed over 2637 differentially expressed genes compared to naturally arising GC cells following activation of naive B cells (**Appendix Table 9**). Despite

expressing canonical GC markers (**Fig. EV 18A**), these cells were transcriptionally highly distinct, indicating that IRF4 plays a role in both plasma cell and GC differentiation (**Fig. 5F**). Similarly, following IRF4 KO in naive cells, we observed a reduction in the proportion of plasma cells and an increase in aberrant GC cells. This suggests that while the plasma cell differentiation was blocked, GC differentiation was impaired, resulting in an aberrant GC state."

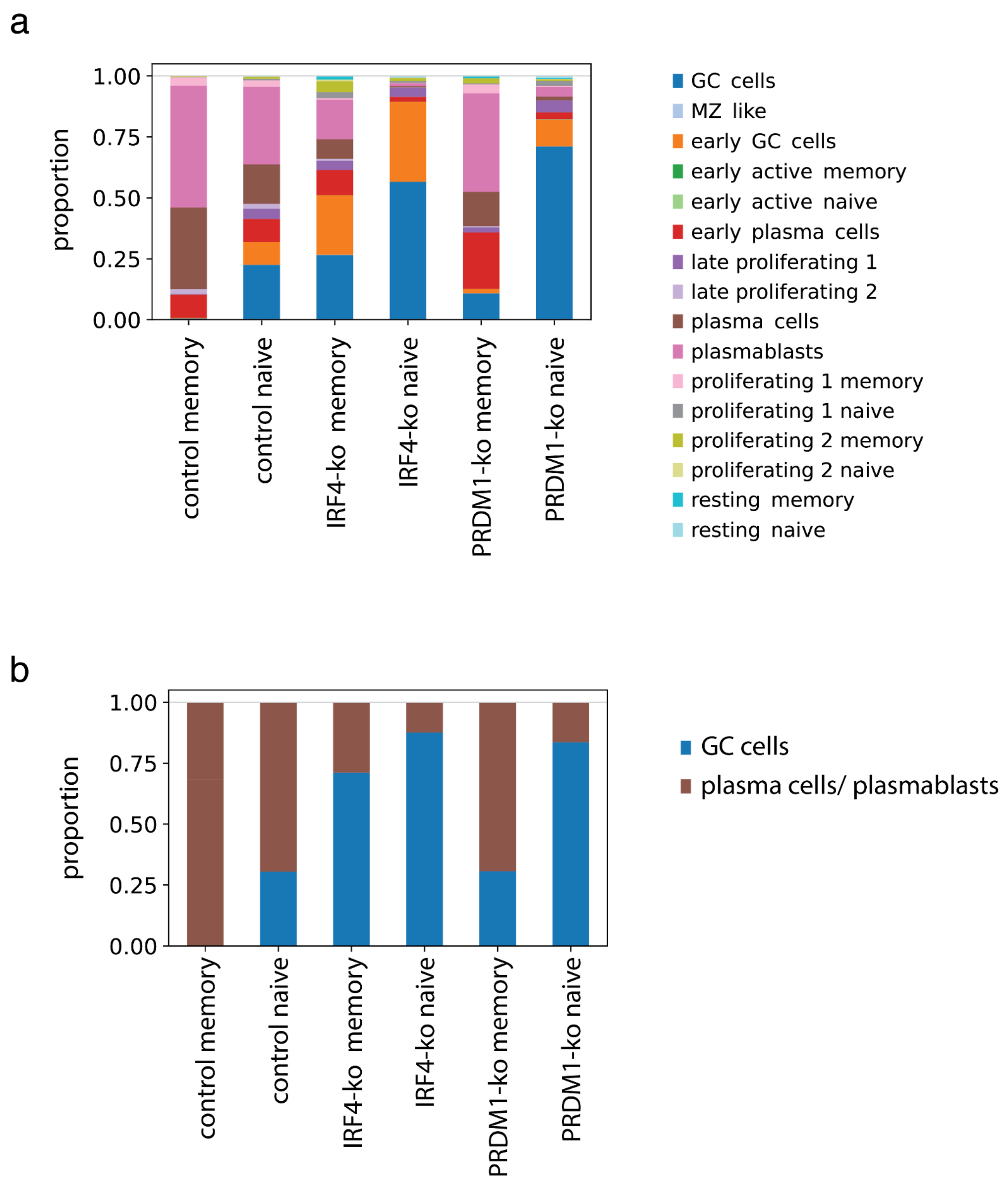

Figure R1. **a**) Barplot showing the fractions of cells from each sample as mapped by scANVI onto the reference dataset. Each bar represents the proportional composition of annotated B cell states within a given KO condition. **b**) Proportion of cells in different manually annotated cell states for naive and memory cells in day 6 KO conditions.

2. Bach2 is an import TF that restrains MBC and naive B to differentiate into PCs (Hu et al. cell reports 2022; Ochiai et al EMBO 2024). It is interesting to provide the detailed analysis of Bach2 (Fig2a and Fig5)

We thank the reviewer for highlighting the important role of BACH2 in B cell fate decisions (Ochiai et al., 2024; Hu et al., 2022). In our analysis, BACH2 was not identified as a regulon by pySCENIC, and we focused our work on the specific regulons that were robustly detected in our dataset, including those associated with IRF4 and PRDM1, which define late differentiation states. Moreover, BACH2 expression is highest in resting B cells and decreases upon activation (**Fig. R2A**). As our knockout system targets cells at day 1 post-activation when BACH2 expression is still near its peak (which is at resting state) interpreting its impact in this setting would be challenging. For these reasons, we did not further experimentally investigate BACH2 in our study.

However, we performed an *in silico* perturbation analysis of BACH2 to evaluate its predicted impact on B cell fate decisions. Specifically, we aimed to assess whether we would recapitulate the reported role of BACH2 in restraining plasma cell differentiation following activation of naive and memory B cells. To model the effect of BACH2 depletion, we applied CellOracle (Kamimoto et al. 2023), a machine learning framework that predicts global gene expression shifts resulting from transcription factor perturbations. Computational knock-out of *BACH2* in day 6 naive B cells predicted a transcriptional shift toward plasma cell fate acquisition, suggesting that BACH2 has a role in inhibiting plasma cell fate (**Fig. R2B and Fig. R2C**). In memory cells, the interpretation is more challenging, as differentiated states form a continuum of plasma cell states (“early plasma cells”, “plasmablasts” and “plasma cells”). Here, *in silico* BACH2 knockout was predicted to promote accumulation of “early plasma cells**”** (**Fig. R2D and Fig. R2E**). However in both cases BACH2 KO implies the role of BACH2 in restraining plasma cell differentiation. Taken together, we recapitulated proposed roles of BACH2.

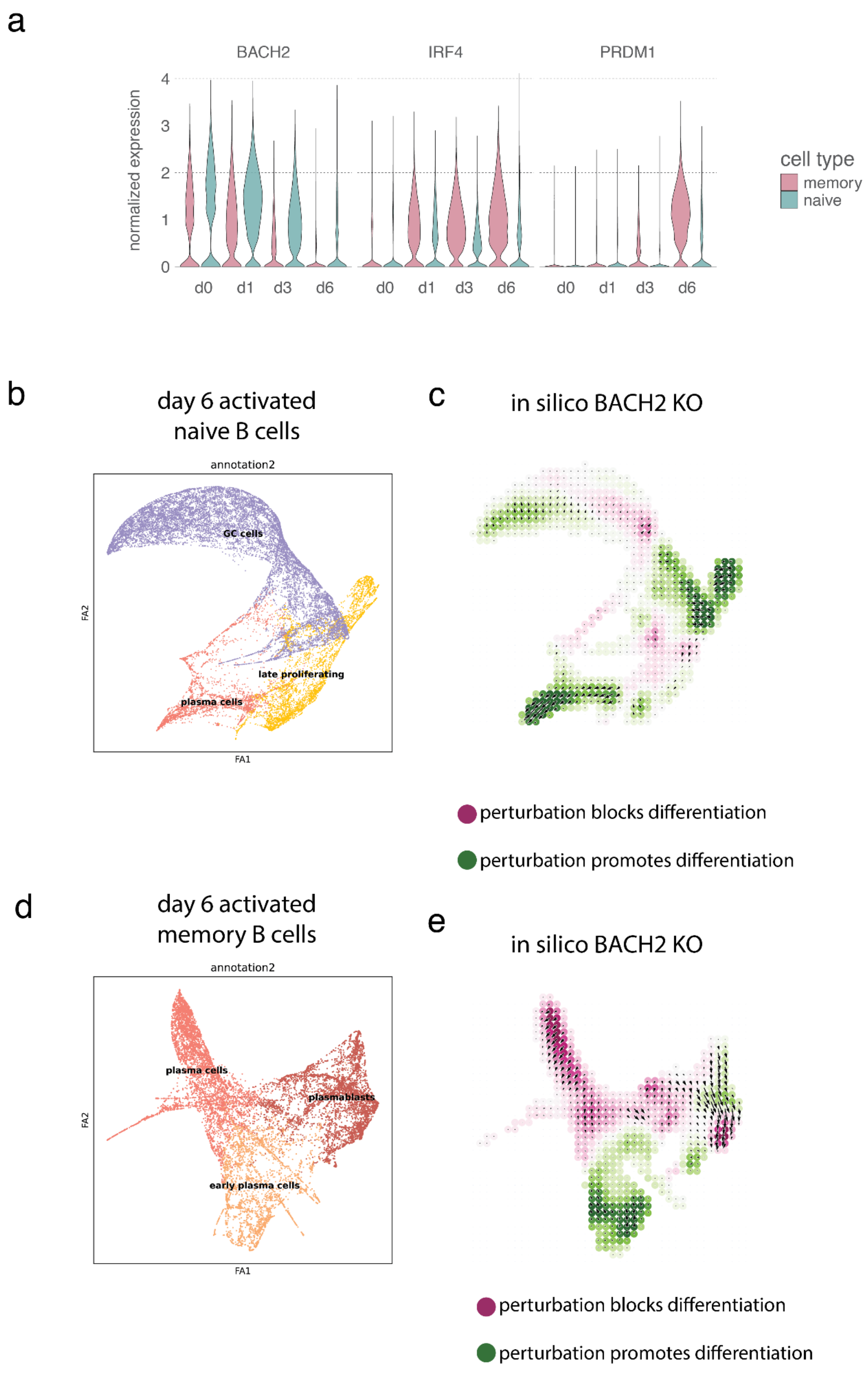

Figure R2. a) Normalized expression level of BACH2, IRF4, PRDM1, SPI1 **b,d**) Force-directed graph embedding of naive B cells at day six after activation. Color represents cell states. **c,e**) CellOracle KO simulation vector field with perturbation scores. Positive perturbation scores are represented in green and indicate that the transcription factor perturbation promotes differentiation. Negative perturbation scores are represented in purple and indicate that the transcription factor perturbation blocks differentiation. Arrows represent simulated cell state transitions following perturbation.

Reviewer #2:

In this paper, the authors presented an investigation of gene regulatory networks controlling naive and memory B cell differentiation using integrated single-cell RNA-sequencing and BCR-sequencing approaches. This work provides valuable insights into the distinct differentiation trajectories of these cell populations and includes functional validation through CRISPR perturbations.

We thank the reviewer for their summary of our study and their recognition of the value of our integrated single-cell and CRISPR perturbation approach.

However, several aspects require clarification and improvement before publication. They are listed below:

1. CRISPR efficiency through PCR and flow cytometry in Methods section, quantitative data on knockout efficiency are not presented in the results. Including specific knockout percentages and representative validation data (e.g., gel images, protein expression levels) in the supplementary materials would strengthen the reliability of the perturbation experiments and enhance reproducibility.

We thank the reviewer for this helpful suggestion. We agree that providing quantitative validation of CRISPR knockout efficiency strengthens the robustness and reproducibility of the perturbation experiments. In response, we have now included explicit knockout efficiency measurements and representative validation data in the supplementary materials. To evaluate on-target gene editing efficiency, Sanger sequencing data from four (IRF4-KO, PPP12RC-KO) and three PRDM1-KO PCR samples, isolated from both naive and memory edited B cells, were analysed with the ICE tool (https://ice.editco.bio/#/). We confirmed the knock-out for both IRF4 and PRDM1 with an average efficiency of 98% for IRF4 and 90% for PRDM1 (Fig. EV 15A and Fig. R3). We have discussed this point in the Results section, where we now describe this as follows:

"By performing PCR and Sanger sequencing we confirmed the knock-out for both IRF4 and PRDM1 with an average efficiency of 98% for IRF4 and 90% for PRDM1 (**Fig. EV 15A**) (Methods). This resulted in a marked reduction in the frequency of plasma cells across both cell types, though the effect of IRF4 KO appeared to be stronger (**Fig. 5A**). "

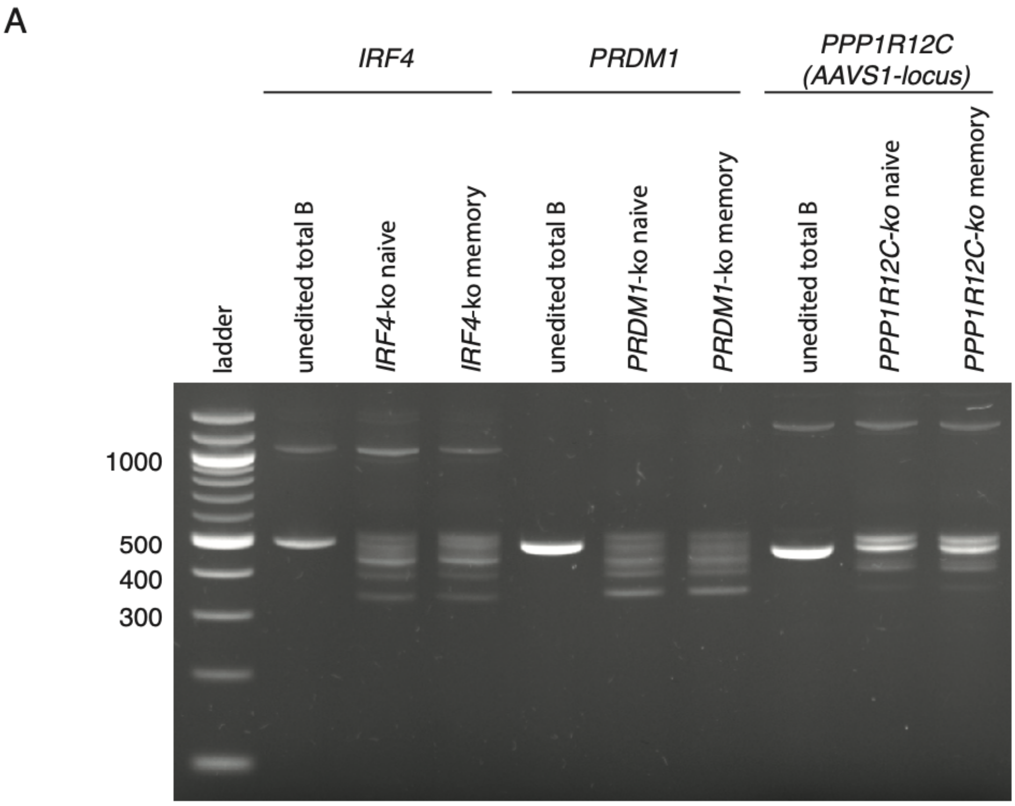

Figure R3. **A**) CRISPR-Cas9 genetic editing of *IRF4*, *PRDM1* and negative control *AAVS1* locus PPP1R12C. A 2% agarose gel image showing the PCR products of representative samples of unedited total B cells and gene-edited naïve and memory B cells.

2. The stimulation cocktail used for B cell activation (including CD40L, IL-2, IL-21, etc.) is described, but it would be helpful to include more justification for the chosen concentrations and their relevance to physiological conditions. A reference to similar studies or a comparison to different concentrations could strengthen this aspect.

We thank the reviewer for their comment and agree with the suggestion. We have revised the manuscript accordingly and added a description of the optimisation of B-cell differentiation culture conditions in (**Fig. EV2** and **Fig. R4**).

Various in vitro activation protocols have been described in the literature, broadly defined by the type of stimulus used. Human B cell stimulation typically relies on a T-cell-dependent signal (CD40L) alone or in combination with a T-cell-independent stimulus such as CpG. However, as noted by (Marsman et al. 2022), using both types of stimuli in the same assay can generate heterogeneous signals that complicate interpretation. We therefore based our system on T-cell-dependent stimulation and cytokines, as CD40L–CD40 costimulation together with IL-21 is critical for B-cell survival, class switching, and germinal-centre maturation (Wu et al. 1995; Ettinger et al. 2005; Ding et al. 2013). This strategy is also supported by several studies (Ettinger et al. 2005; Marsman et al. 2022; Kuchen et al. 2007). Additional cytokines can synergise with IL-21; for example, IL-2 enhances IL-21 activity by

inhibiting BACH2 and thereby promoting PRDM1 expression (Le Gallou et al. 2012; Hipp et al. 2017).

Building on these reports, we optimised a protocol suitable for both naive and memory B cells that drives clear, division-associated transcriptomic changes and enables us to identify discrete stages of differentiation. Our protocol uses a multimeric CD40L reagent (megaCD40L, Ezio Science) together with recombinant IL-21, IL-2, and a BCR stimulus (anti-IgM), as BCR engagement is an early and necessary event in differentiation. Anti-IgM, combined with costimulatory signals and cytokines, enhances differentiation (Ettinger et al. 2005) and provides protection from Fas-mediated apoptosis (Wang et al. 2000).

To ensure a uniform starting point and avoid asynchronous responses, we added the stimulation cocktail only once at culture initiation. This is consistent with observations that termination of CD40L signalling downregulates PAX5 and upregulates PRDM1, thereby promoting class switching (Marsman et al. 2022). As the costimulatory signal naturally decreases over time, a single initial stimulus supports controlled progression.

Fig. R4 (**Fig. EV2** in manuscript) shows proliferation and survival of naïve B cells (three donors) across serial dilutions of megaCD40L, IL-21, IL-2, and anti-IgM. As expected, both megaCD40L and IL-21 were essential for survival and division, and IL-2 enhanced survival at the chosen concentration. High megaCD40L levels (1 µg/mL) reduced survival, suggesting signal saturation. Anti-IgM did not affect division but modestly improved survival between 2–5 µg/mL, with higher doses again decreasing survival. Culture conditions were validated at seeding density of 50,000 cells.

We have discussed this in the results section as follows:

"Our data indicate that CD40L and IL-21 are fundamental for driving B cell proliferation, while IL-2 primarily enhances B cell survival (**Fig. EV 2 A-B)**. Since we isolated naive and unswitched memory B cells, we engaged the BCR with IgM cross-linker, although the strength of BCR stimulation played a lesser role in regulating B cell proliferation and survival (**Fig. EV 2 A-B**)."

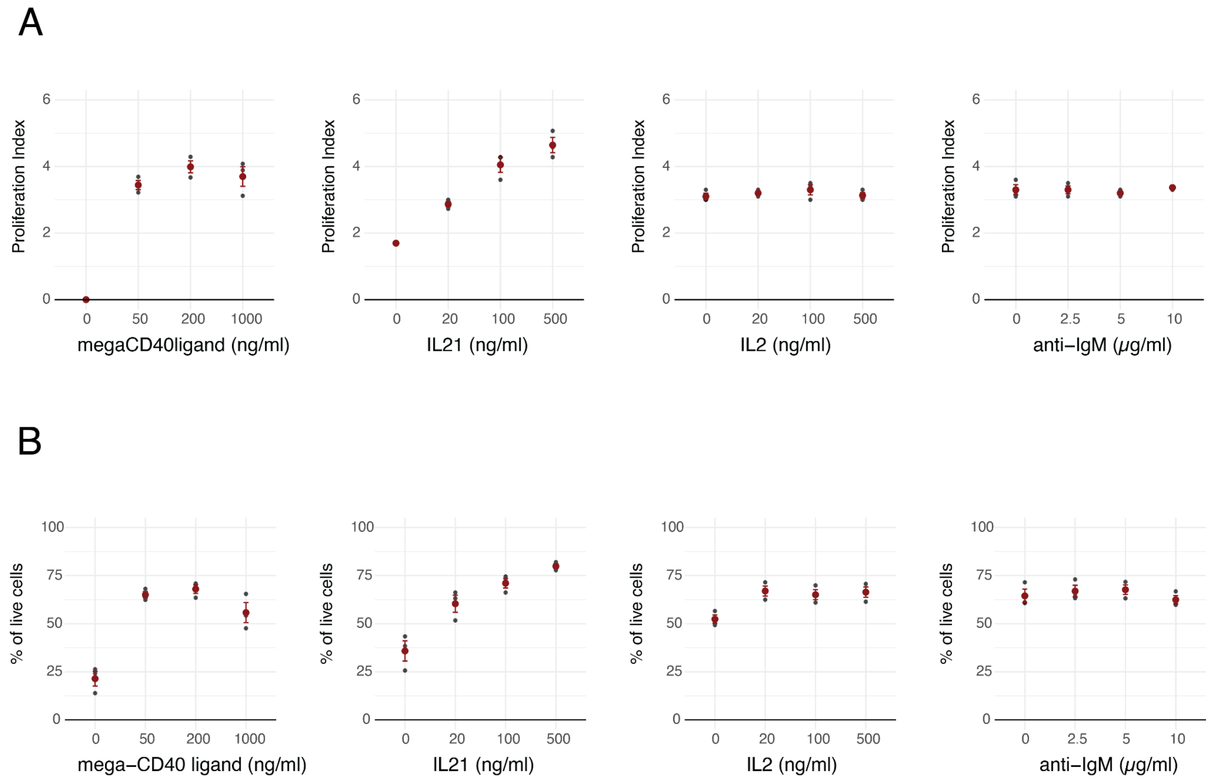

Figure R4. Plots illustrating the results of the titration of IL21, IL2, megaCD40ligand and anti-IgM. For each reagent, four different concentrations were tested while keeping the others fixed (200 ng/mL megaCD40L, 5µg/mL anti-IgM, 100 ng/mL IL-21, and 20 ng/mL IL-2). Proliferation index **A**) and percentage of live cells **B**) were quantified. Mean and standard error are shown for 3 different biological replicates.

3. Although some quality control steps for the scRNA-seq data are mentioned, such as cell filtering and doublet removal, the specific quality control criteria and methods are not detailed. It is recommended to provide a more thorough description of the quality control process in Results section or supplementary materials, including the specific criteria for cell selection (such as gene count, mitochondrial gene proportion, etc.), thresholds for doublet removal, and the methods used for data normalization. Additionally, quality control plots or relevant numerical data should be included to enhance the reproducibility and transparency of the data.

We thank the reviewer for this valuable suggestion. We agree that providing additional details on the scRNA-seq quality control steps will enhance reproducibility and transparency. In the revised manuscript, the Methods section has been substantially expanded to include specific criteria for cell selection (e.g., gene count, mitochondrial gene proportion), thresholds for doublet removal, and normalization procedures. Additionally, we have included in the manuscript **Fig. EV3A-C** and Appendix Table 10 providing detailed quality control metrics. To further support transparency, we released a GitHub repository containing all analysis codes along with per-sample quality control plots.

4. The neural network model predicting sister cell relationships based on transcriptomic data is interesting. However, the manuscript would benefit from additional validation of this prediction. Can the authors provide an independent dataset or validation experiment that confirms the prediction model's accuracy in predicting sister cells?

We thank the reviewer for this suggestion. To validate our neural network model predicting sister cell relationships, we analyzed an independent publicly available dataset of 5' scRNA-seq and scVDJ data from bone marrow B cells of 11 mice (Agrafiotis et al. 2023); ArrayExpress accession E-MTAB-12610). We applied both our neural network (NN) model and a logistic regression (LR) model (as suggested by Reviewer 3) to classify sister cell relationships across these 11 independent in vivo clonal datasets. In 7 out of 11 datasets, both models achieved classification accuracy above 70%, while all datasets showed accuracy above 50% (NN: 0.72 ± 0.098; LR: 0.71 ± 0.10; **Fig. R5** and **Fig. EV12**), demonstrating that our models generalize across independent biological samples.

To ensure that these results reflect genuine clonal signatures rather than random chance, we repeated the analysis using 100 random clonal assignments within each mouse. In this case, the classifiers failed to detect any meaningful patterns (**Fig. R6** and **Fig. EV13**), confirming that the models are learning biologically relevant clonal relationships. These results provide strong independent validation that transcriptomic signatures of sister cells can be robustly captured by our models, even in independent *in vivo* datasets.

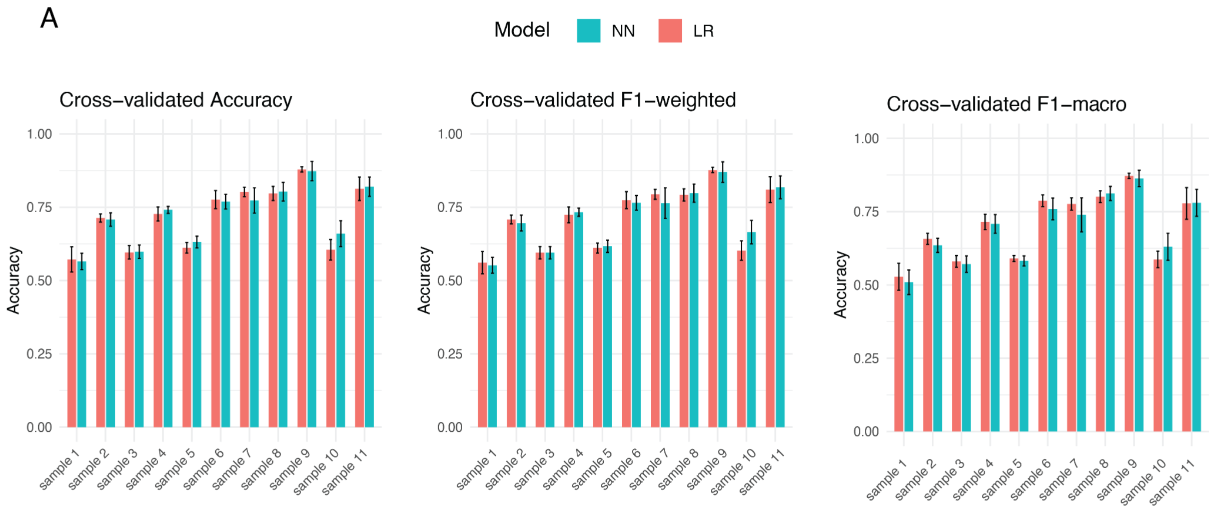

Figure R5. **A**) Bar plot showing the performance (accuracy, F1-score) of various models used to predict clonal identity based on gene expression profiles on 11 mice B cell samples from (Agrafiotis et al. 2023) . Each bar represents the mean and standard error of the performance metric across cross-validation folds (K=5). Models compared include logistic regression (LR) and a neural network (NN).

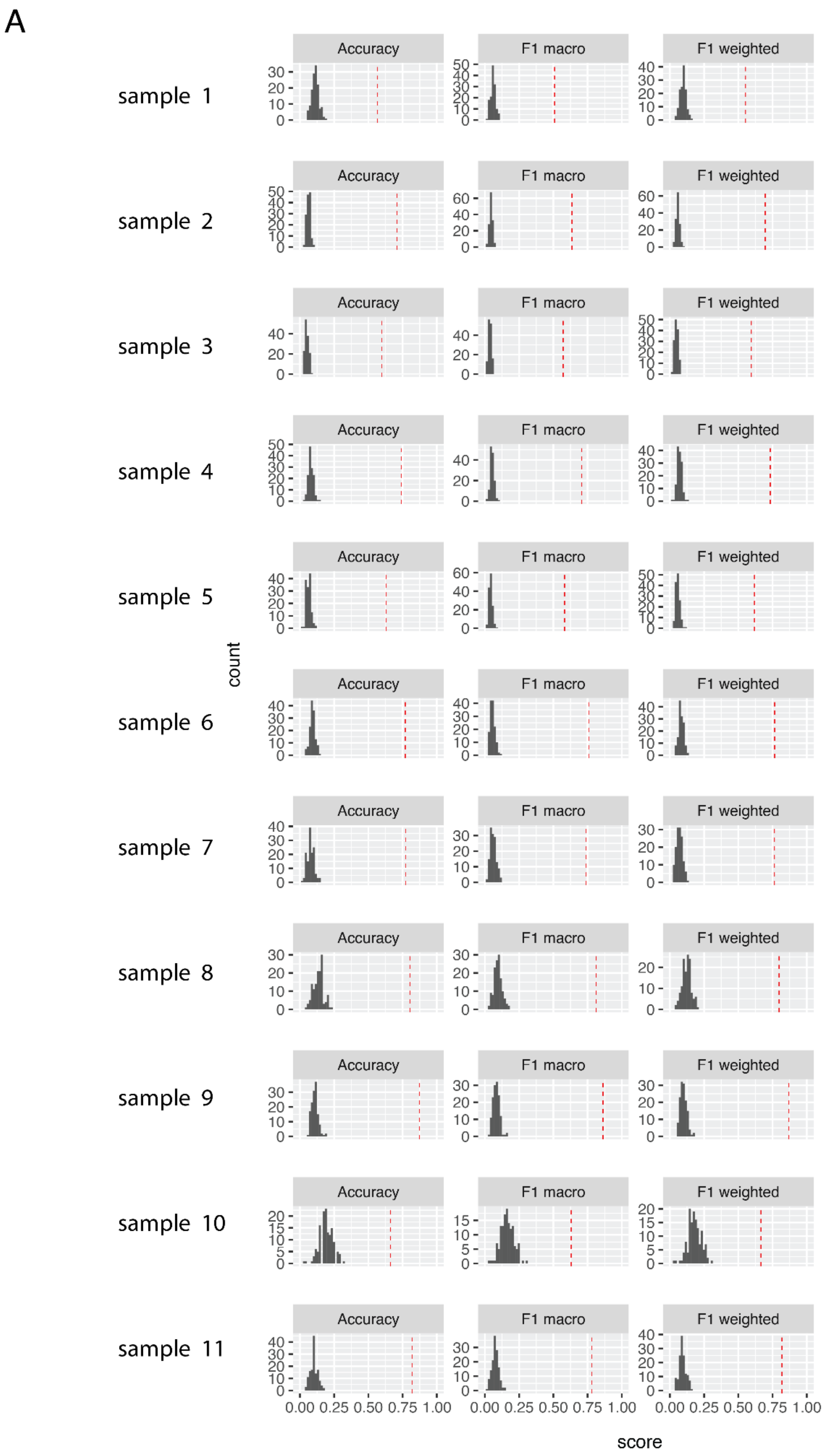

Figure R6. **A)** Distribution of accuracy metrics (accuracy, F1-score) of the neural network used to predict clonal identity based on gene expression profiles, estimated 100 times with randomised clone-ids within each mice B cell clonal dataset. Random distribution was estimated by shuffling the clone-ids within each mice and running the classification 100 times. Red lines represent the accuracy metric obtained on the non-randomised data.

5. The authors have performed several important validation experiments (e.g., ImageStream analysis of IRF4/SPI1 localization) that support their main conclusions. However, the presentation of these validation data could be optimized, potentially by promoting some supplementary validation results to main figures to better support the central claims.

We thank the reviewer for this suggestion. We have now included additional Imagestream analysis conducted on three different donors in naive and in memory B cells (**Fig. R7** and **Fig. EV8**). These results demonstrate that cells expressing high levels of IRF4 show low levels of SPI1, and vice versa. Across all three donors, naive B cells produced a higher fraction of $SPI1^{high}$ cells and a lower fraction of $IRF4^{high}$ cells, supporting our scRNA-seq annotations. In contrast, memory B cells gave rise to a higher fraction of $IRF4^{high}$ cells compared to naive B cells, along with a substantially lower fraction of $SPI1^{high}$ cells. Moreover, the ImageStream analyses confirmed the mutually exclusive nuclear localization of IRF4 and SPI1 (**Fig. R7** and **Fig. EV8**). Taken together the imagestream results support a model of differentiation where B cells can acquire two fates, GC cells and plasma cells, with memory B cells poised to differentiate towards plasma cells while naive B cells showing ability to give rise to both GC cells and plasma cells.

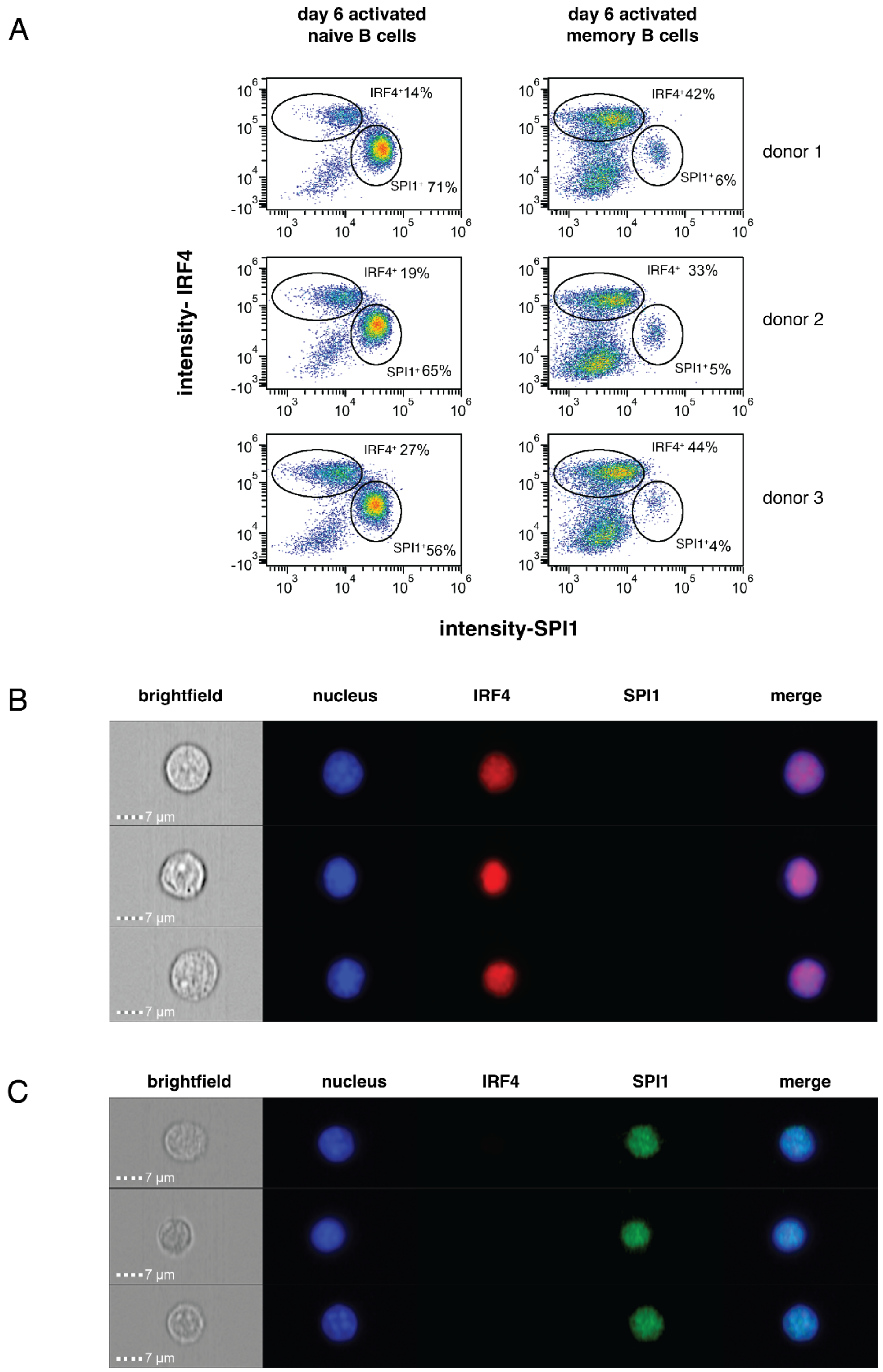

Figure R7. A) ImageStream intracellular staining of IRF4 and XBP1 in naive and memory B cells at day six after activation, n=3. **B-C**) Representative ImageStream images of activated memory and naive B cells at day six stained for nucleus (Draq5), IRF4 and SPI1. Images were acquired at 60× magnification.

6. Some conclusions appear to interpret correlative findings as causal relationships. The manuscript would benefit from more cautious language when describing regulatory relationships, particularly distinguishing between observations of gene expression correlations and direct evidence of regulatory control.

We agree that this is one of the limitations of our study. We have added the following paragraph to the Discussion to explicitly acknowledge this limitation:

"While our integrative single-cell transcriptomic and clonal-tracking approach provides a comprehensive view of the gene regulatory networks underlying B cell fate decisions, it should be acknowledged that GRN inference from scRNA-seq data is inherently correlative. Although our knockout experiments for IRF4 and PRDM1 support their involvement in shaping differentiation trajectories, these perturbations do not establish direct regulatory interactions between transcription factors and specific target genes within the predicted networks. Future work employing genome-wide TF binding assays (e.g., CUT&RUN, CUT&Tag, ChIP-seq), paired perturbation and reporter assays, or high-throughput CRISPR screens will be required to definitively map causal regulatory relationships."

Despite these concerns, the integration of transcriptomic and clonal tracking data, combined with functional perturbations, provides valuable mechanistic insights.

We thank the reviewer for emphasizing the value of our study.

Reviewer #3:

Summary

Establishing a rigorous map of the gene-regulatory programs that govern human B-cell fate is of broad interest for immunology and systems biology. The manuscript "Competing gene regulatory networks drive naive and memory B-cell differentiation" profiles naive and memory B cells upon activation (10x 5' scRNA-seq coupled with scBCR-seq), infers TF activities and regulons, and combines this with CRISPR perturbations of IRF4 and PRDM1. The authors report a naive bifurcation into germinal center-like (GC-like) and plasma cell (PC) states versus a memory bias toward PCs, anticorrelated IRF4-SPI1 activity, germline transcript (GLT) enrichment in GC-like cells, and clonal transcriptomic heritability supported by a neural-network classifier.

The study is potentially important. However, there are concerns regarding the depth and rigor of key analyses and validations. Detailed comments follow.

We thank the reviewer for this thoughtful summary of our work and for highlighting the value of our study.

Major concerns:

1. Absence of trajectory/fate modeling

The bifurcation claim would be much stronger with dynamical evidence. Please compute RNA velocity for naïve and memory B-cell populations separately (days 0/1/3/6) and use CellRank to estimate absorption probabilities toward PC vs GC-like endpoints. Map IRF4 and SPI1 dynamics along pseudotime and quantify early (day 1/3) fate biases to further support the manuscript's claim of anticorrelated IRF4-SPI1 activity.

We agree with the reviewer that trajectory modeling would provide an additional and valuable layer of evidence supporting the fate decisions undertaken by B cells upon activation. RNA velocity is indeed a powerful approach for modeling dynamic cell-state transitions. However, RNA velocity assumes a continuous transcriptional manifold between timepoints, which is not fulfilled in our dataset. Our experimental design consists of discrete and relatively distant timepoints (days 0, 1, 3, and 6), resulting in limited transcriptional overlap between them. This violates the continuity assumption required for reliable RNA velocity inference and would likely yield misleading velocity fields rather than biologically meaningful trajectories. From La Manno et al manuscript:

"Metabolic labelling showed that for most genes, changes in the spliced/unspliced ratio would be detectable after 10–100 min (Extended Data Fig. 2). The effective timescale of extrapolation, on the other hand, depends on the biological process that is analysed. On the

basis of pulse labelling of chromaffin progenitor cells with 5-ethynyl-2′-deoxyuridine (EdU) (Supplementary Note 2 Section 6), we estimate that we were able to extrapolate 2.5–3.8 h into the future (Fig. 2f, g), which is also consistent with the ability to resolve cell-cycle events. Given the linear nature of the extrapolation, however, this predictive timescale will depend on the shape of the gene expression trajectory (that is, the curvature of the expression manifold). Cell fates can be predicted over longer time scales by tracing a sequence of small extrapolation steps on the observed expression manifold (Supplementary Note 2 Section 7).” (La Manno et al. 2018).

Nevertheless, to address the reviewer’s suggestion and strengthen our conclusions, we implemented Moscot (Klein et al. 2025), a recently developed method for modeling cellular fate transitions based on Optimal Transport theory (Klein et al. 2025). Moscot explicitly incorporates timepoint information and computes transition probabilities between cells across timepoints by solving an optimal transport problem. The method first constructs cost matrices representing the “distance” (in transcriptomic space) between cells from consecutive timepoints, and then estimates transition matrices reflecting the most probable state transitions across time. Using Moscot, we quantified fate biases of naive and memory B cells from day 3 to day 6. The model predicted that plasma cell progenitors arise predominantly from day 3 memory B cells, whereas GC cells originate almost exclusively from day 3 naive B cells (**Fig. R8** and **Fig. EV5)**. Plasmablasts, in contrast, were predicted to derive from both day 3 naive and memory B cells. These results are consistent with our main conclusion that naive B cells can bifurcate toward either GC or plasma cell/plasmablast fates, while memory B cells are primarily biased toward plasma cell differentiation.

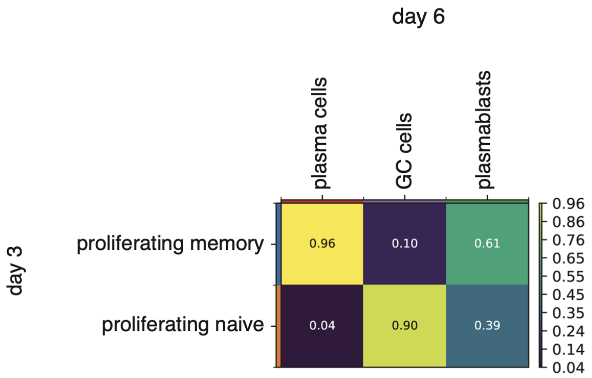

Figure R8. Moscot-calculated transition matrix showing the ancestry of cells at day 6 cells. Ancestor cell states at day 3 are on the rows and descendant cell states at day 6 are on the columns.

To further investigate early bifurcation within the naïve B-cell compartment, we computed pseudotime trajectories using Palantir (Setty et al. 2019). Palantir computes pseudotime as a

relative distance from an initial reference cell, independently of its specific lineage. This analysis revealed two diverging trajectories emerging from naive B cells: one leading toward plasma cell identity and the other toward GC fate (**Fig. R9** and **Fig. EV7B-C**). Mapping IRF4, PRDM1, and SPI1 expression dynamics along these two pseudotime branches highlighted distinct transcriptional programs: IRF4 and PRDM1 were upregulated along the plasma cell trajectory, while SPI1 was upregulated along the GC trajectory. These opposing dynamics support our conclusion of anticorrelated IRF4–SPI1 activity and reinforce the existence of two transcriptionally distinct differentiation pathways emerging from naive B cells.

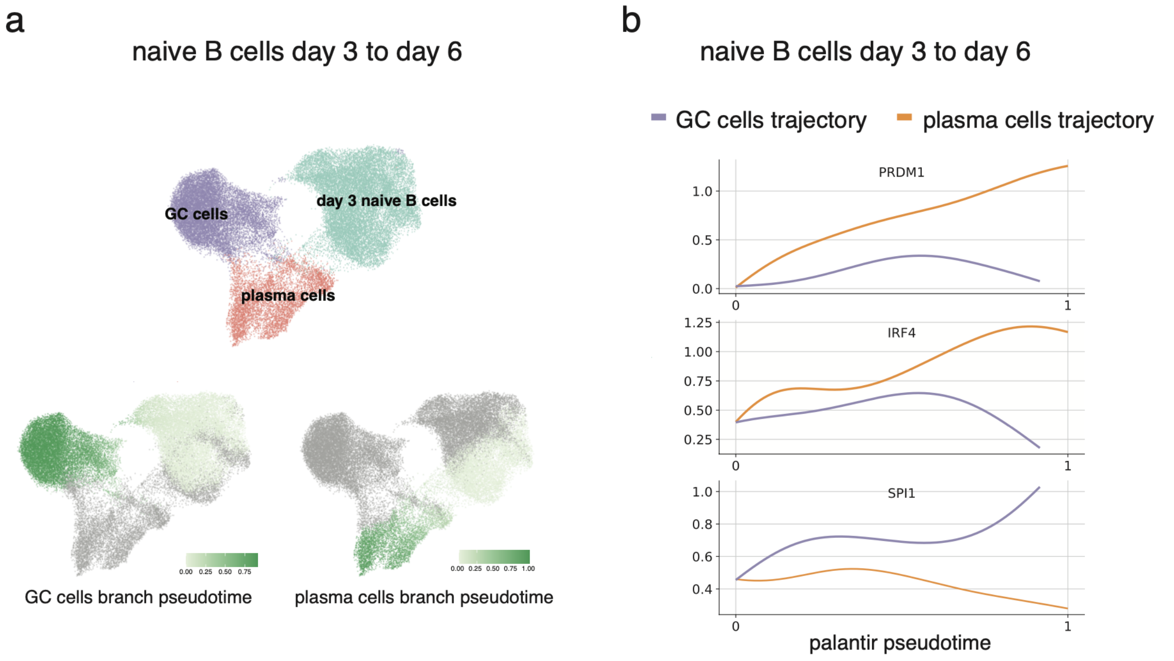

Figure R9. a) UMAP embeddings of naive B cells at day 3 and 6. Top UMAP shows the annotation. Bottom right and left UMAP represent the Palantir pseudotime across the two naive B cell pseudotime branches. Pseudotime is represented by a light green to dark green scale, with darker shades indicating higher pseudotime values **b**) Gene trends along the pseudotime for each naive B cell lineage

2. Lack of independent GRN confirmation

SCENIC on SEAcells (30 runs; 80% regulon retention) is solid, but TF-activity claims should be corroborated with an independent framework. Please add DoRothEA + VIPER and/or Pando and provide concordance metrics (rank correlations, top-k overlaps, permutation p-values), highlighting agreements/disagreements for IRF4/SPI1/XBP1.

We thank the reviewer for this constructive suggestion to validate our transcription factor (TF) activity inferences with an independent framework. Regulon annotations are highly context-

dependent, often varying across cell types and activation states (Pett et al. 2025). Therefore, to ensure that our analysis reflects B cell–specific regulatory programs, we incorporated a curated B cell–specific regulon annotation (Basso et al. 2005). We then estimated TF activity using VIPER based on this curated network. To assess concordance between SCENIC- and VIPER-derived results, we focused on the 59 TFs common to both annotations. We computed Pearson correlations between normalized, ranked regulon specificity scores across cell states. The average correlation coefficient was 0.42 (**Fig. R10**). Given the inherent methodological differences and non-overlapping gene sets, this correlation can be considered robust and indicative of consistent regulatory signal capture. When comparing the top 20% regulons across the two datasets (621 from Basso et al. vs. 72 from SCENIC), approximately half overlapped (**Fig. R11**), consistent with expected variation due to differences in regulon definitions and sizes.

Finally, to specifically assess the anticorrelation between IRF4 and SPI1 activities, we compared VIPER-inferred TF activity scores derived from the Basso et al. annotation. This analysis confirmed a significant negative correlation between IRF4 and SPI1 activities (**Fig. R12** and **Fig. EV7A**), consistent with our SCENIC results. Moreover, IRF4 and XBP1 VIPER scores were positively correlated (**Fig. R12** and **Fig. EV7A**). Together, these results demonstrate strong cross-method concordance and independently validate our key conclusions regarding the opposing activities of IRF4 and SPI1 and the cooperative role of IRF4 and XBP1 in plasma cell differentiation.

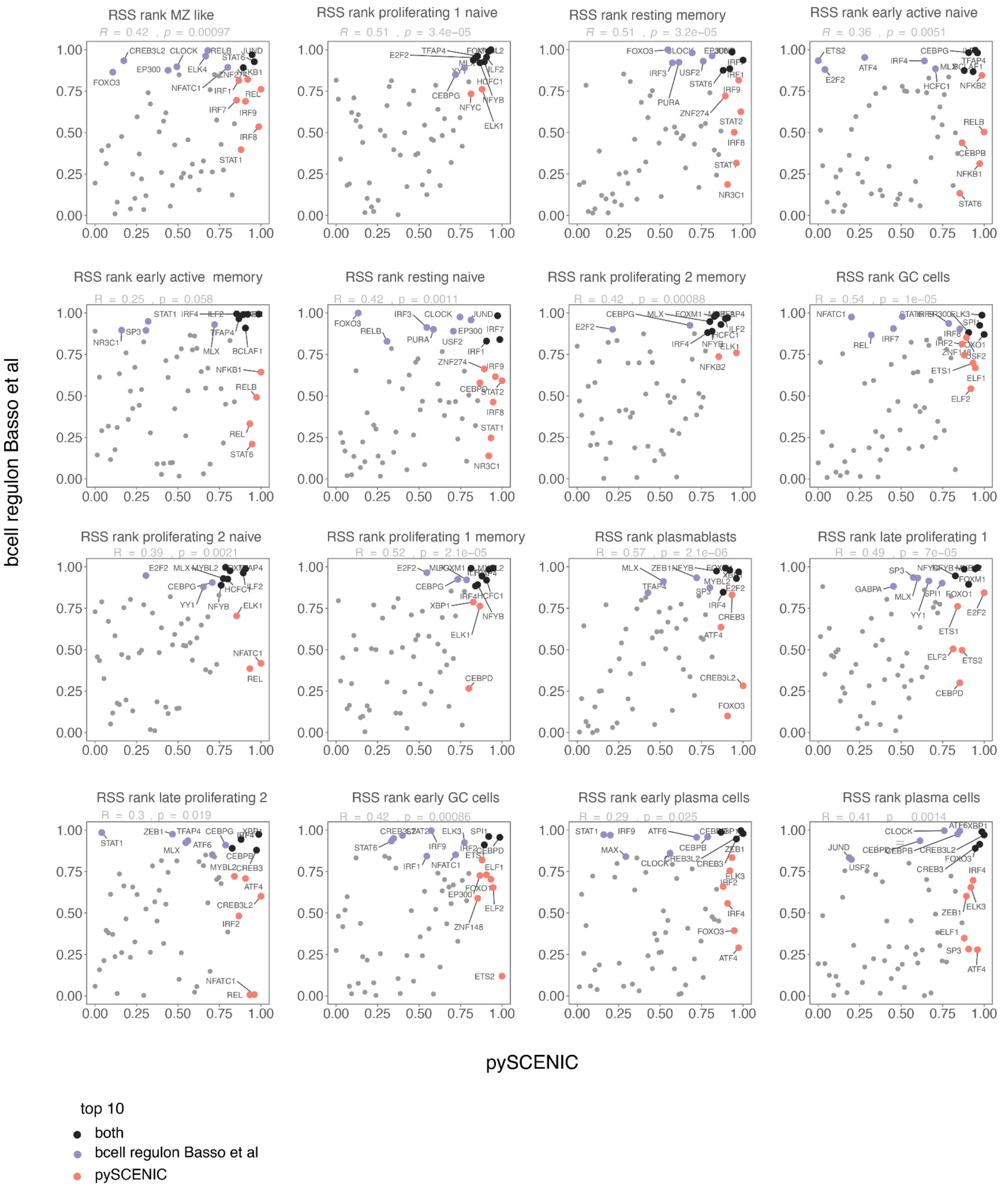

Figure R10. Comparison of regulon specificity scores using published regulon annotations (Basso et al 2005, Nature Genetics) and our cell model. The suffix -dc and -pc indicates Basso et al and our pySCENIC annotation respectively. Pearson correlation coefficients and corresponding p-values are shown. Top 10 regulons are highlighted for each annotation. Orange indicates top 10 pySCENIC derived regulons by rank; purple indicates top 10 Basso et all regulons by rank; black indicates that the regulon is in the top 10 in both annotations.

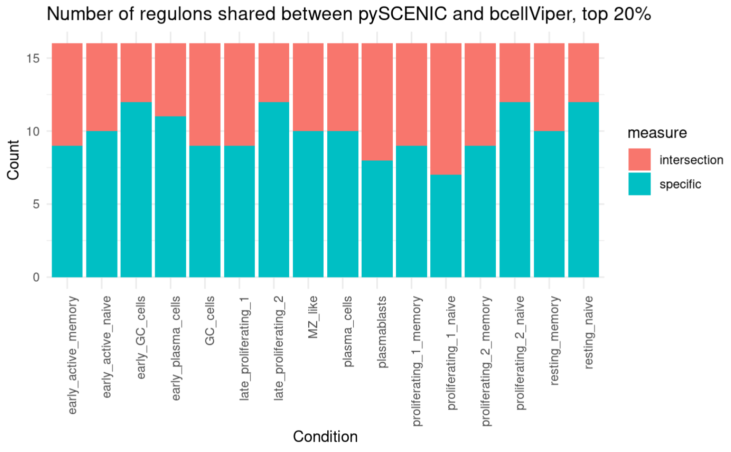

Figure R11. Barplot illustrates whether the top 20% regulons identified with pySCENIC were among the top 20% of Basso et al. Red and blue represent shared and not shared regulons respectively.

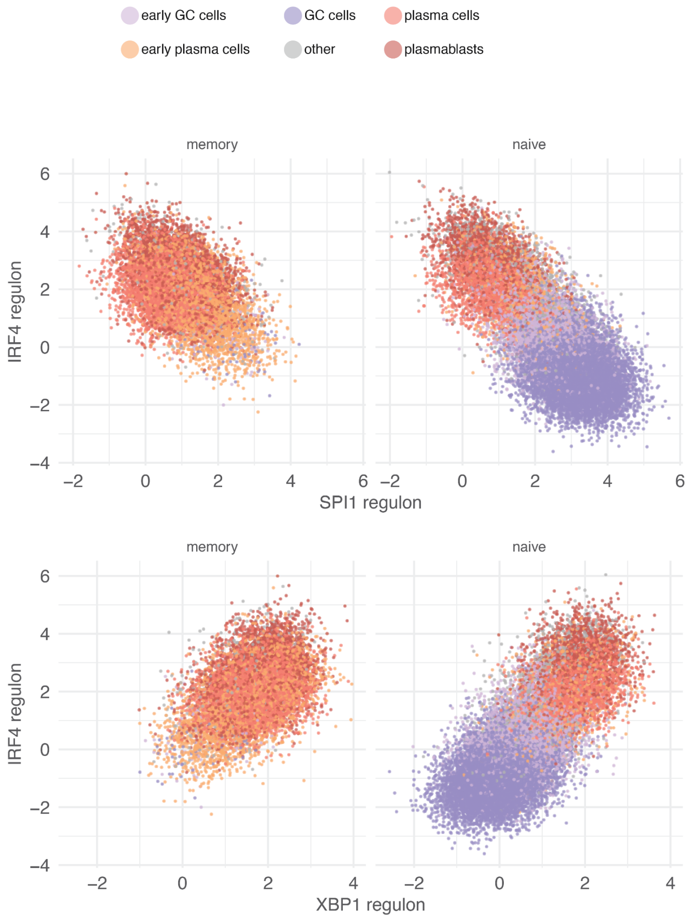

Figure R12. Dot plots showing the SPI1, IRF4 and XBP1 regulon scores in day six naive and memory B cells. Regulon annotations from Basso et al 2005, were scored on our cell model with the VIPER method from the decoupler python package.

3. Limited KO validation and causal modeling

The KO data are interesting, but the link to network perturbation is not established. Please perform in silico perturbations with CellOracle to complement the KOs: single-gene KOs for IRF4, PRDM1, SPI1, and a double KO (IRF4+PRDM1) to test whether PRDM1 loss adds beyond IRF4 loss. Compare predicted state probabilities with your observed KO data. This will directly support the network-causality narrative.

We thank the reviewer for this constructive suggestion. To establish a causal link between transcription factor perturbation and B cell fate decisions, we performed *in silico* perturbation analyses using CellOracle, a machine-learning framework that predicts genome-wide transcriptional shifts following virtual gene perturbations.
Our regulon analysis had previously identified IRF4 and SPI1 as opposing regulators of B cell differentiation, with IRF4 active in plasma cell states and SPI1 in GC cells. To assess whether perturbing these factors could change cell identity, we simulated *in silico* knockouts of IRF4 and SPI1 in day 6 naive and memory B cells, where their activities peak. As expected, simulation of IRF4 loss shifted the transcriptomes of both naive and memory B cells away from plasma cell states, predicting a strong inhibition of plasma cell differentiation (**Fig. R13C** and **Fig. EV17C**). Conversely, SPI1 knockout inhibited GC fate acquisition and shifted naive B cells toward plasma cell-like states (**Fig. R13B** and **Fig. EV17B**). In memory B cells, SPI1 loss promoted early plasma cell differentiation. We next simulated PRDM1 (*BLIMP1*) knockout, a transcription factor uniquely required for plasma cell differentiation. CellOracle predicted that PRDM1 loss would specifically inhibit plasma cell fate acquisition in both naive and memory B cells (**Fig. R13D** and **Fig. EV17D**).
Next, to validate the predicted transcriptional shifts from CellOracle, we compared the simulations with the results from CRISPR-Cas9 knockouts of IRF4 and PRDM1. We added this text to results section:

"Finally, to confirm that IRF4 and PRDM1 impairment induced a rewiring of B cells gene regulatory network, we performed an in silico knock out of IRF4 and PRDM1 to simulate cell state transitions with CellOracle (Kamimoto et al. 2023). These simulations predicted that IRF4 and PRDM1 loss would suppress plasma cell differentiation, that SPI1 loss would impair GC fate acquisition (**Fig. EV 17A-D**). Our experimental KO data are in line with these predictions, exhibiting the expected shifts in cell identity."

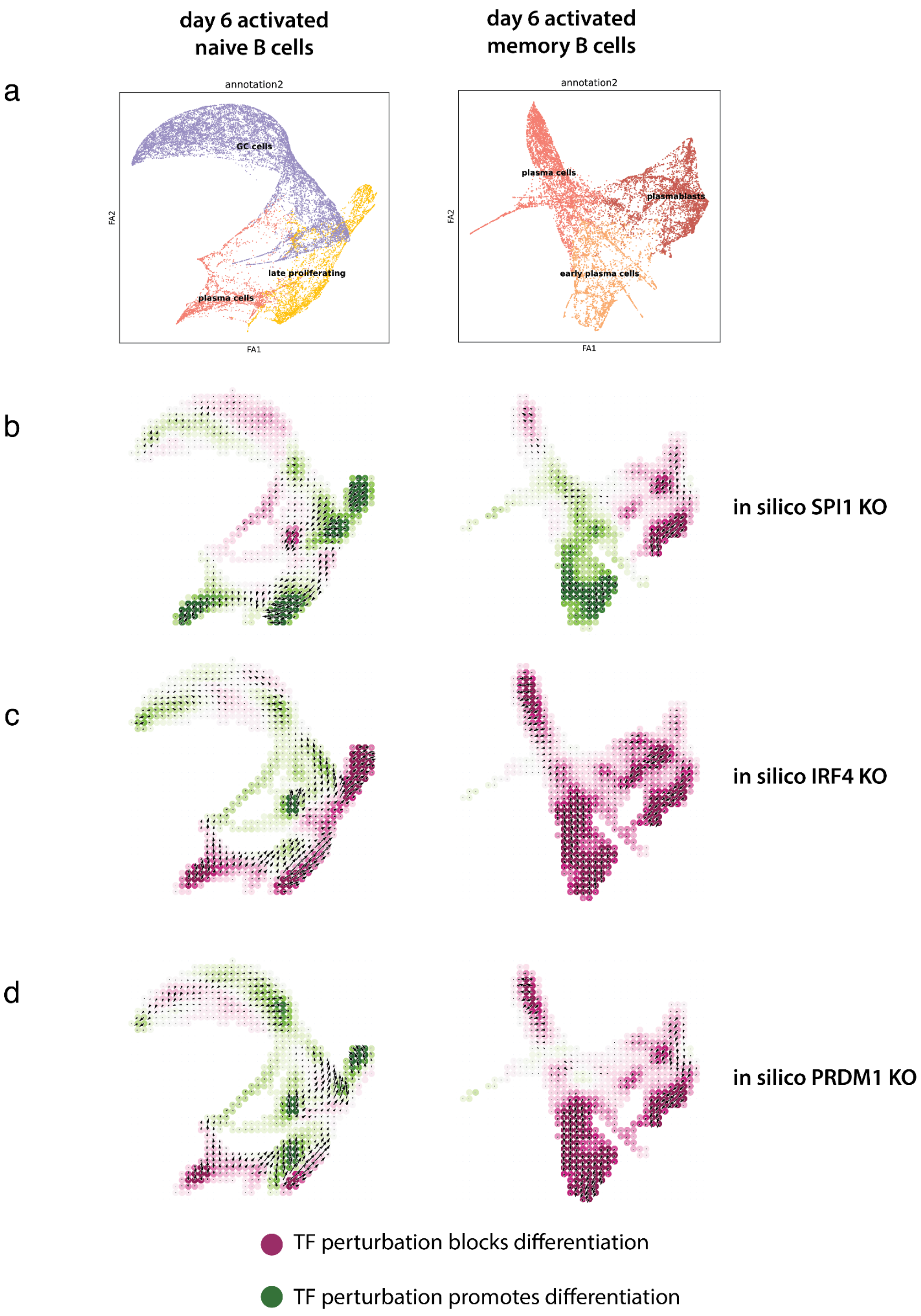

Figure R13. **a,b**) Force-directed graph embedding of naive and memory B cells at day six after activation. Color represent cell states. **b-d**) CellOracle KO simulation vector field with perturbation scores. Positive perturbation score are represented in green and indicate that the transcription factor perturbation promotes differentiation. Negative perturbation score are represented in purple and indicate that the transcription factor perturbation blocks differentiation. Arrows represent simulated cell state transitions following perturbation.

Interestingly, when comparing the in silico PRDM1 and IRF4 knock outs in day 6 naive B cells, we noticed that in silico PRDM1 knock-out would inhibit only plasma cell differentiation while in silico IRF4 knock out would affect also other late naive B cell states like late proliferating cells (**Fig. R13** and **Fig. EV17**), suggesting that IRF4 might play a broader role in B cell differentiation than PRDM1 in naive B cells. Thus, we leveraged our in vitro knock-out experiment to compare the transcriptomes of the GC cells that arose upon IRF4 and PRDM1 knock-out in both naive and memory B cells. Finally, we performed a double *in silico* knockout of IRF4 and PRDM1. The combined perturbation did not produce additive effects beyond PRDM1 single KO in either naive or memory B cells (**Fig. R14**), suggesting that PRDM1 acts downstream of IRF4 in enforcing plasma cell identity.

Thus, the in silico predictions fully matched the observed phenotypes at the experimental level and provided complementary validation of the gene regulatory network control of B cell fate. However we could not disentangle a cumulative IRF4 and PRDM1 effect as the double in silico knock-out of PRDM1+IRF4 was comparable to the PRDM1 single in silico knock-out in both naive and memory B cells (**Fig. R13-R14**).

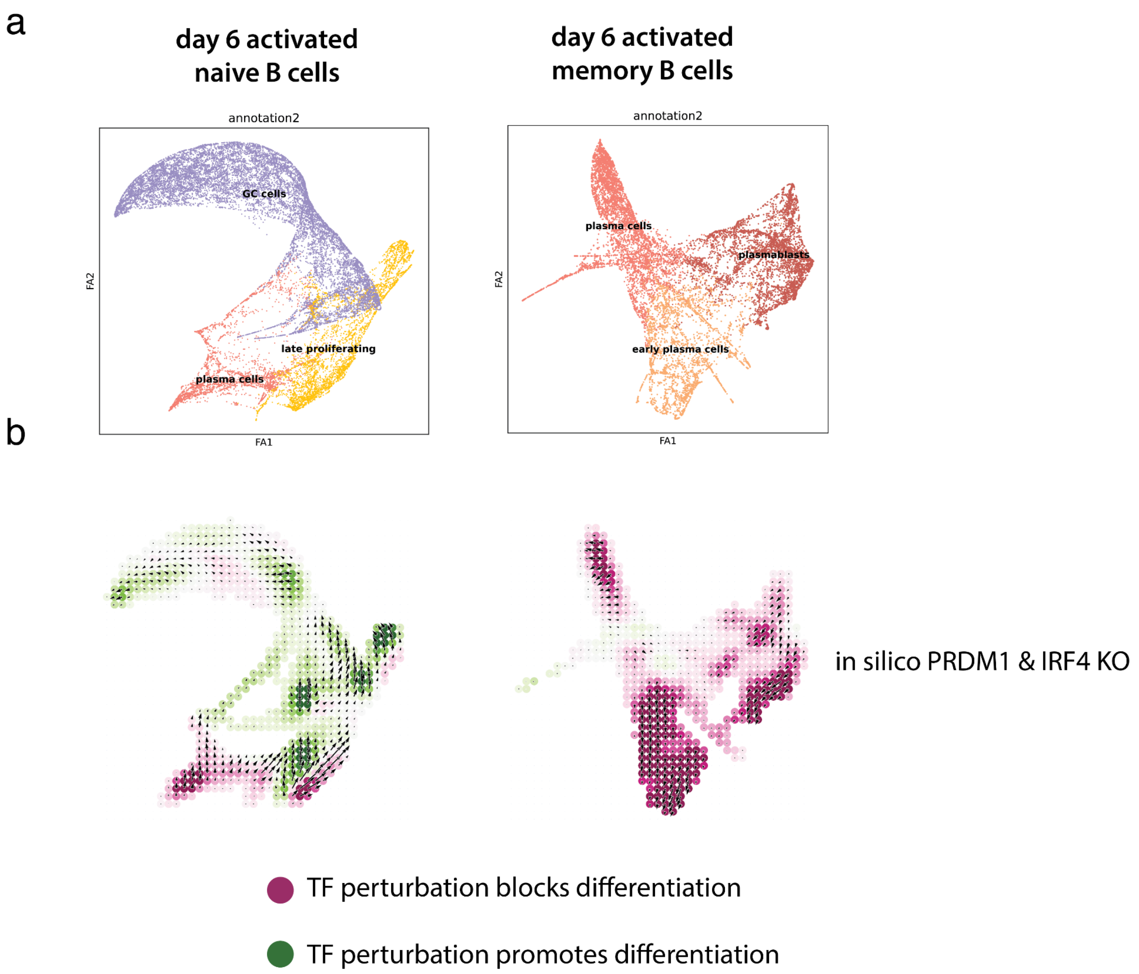

Figure R14. **a,b**) Force-directed graph embedding of naive and memory B cells at day six after activation. Color represents cell states. **b-d**) CellOracle KO simulation vector field with perturbation scores. Positive perturbation score are represented in green and indicate that the transcription factor perturbation promotes differentiation. Negative perturbation scores are represented in purple and indicate that the transcription factor perturbation blocks differentiation. Arrows represent simulated cell state transitions following perturbation.

4. Insufficient justification and benchmarking of the sister-cell neural network
You use a 4-layer FFNN with dropout 0.4 and features selected by interclonal correlation (r>0.25), but there is no justification of this architecture nor reporting of full performance metrics. Please benchmark against baselines (kNN on PCs, logistic regression, random forest), report accuracy/AUROC/F1 and calibration, and include an ablation regressing out cell-cycle and PC/GC markers to show the model is not simply recapitulating cell state.

We thank the reviewer for the valuable suggestion. We have now conducted an extensive validation of the prediction of clonal identity solely using gene expression.
To predict clonal identities, we compared the performances of three different machine learning methods, a neural network (NN), a multinomial logistic regression model (LR) and a

random forest classifier (RF). For all the models we now report k=5 cross validation mean accuracy, F1 weighted, F1 macro and Brier Score loss for calibration.

Firstly, we set out to identify a minimal set of genes which were necessary and sufficient to classify clones only with gene expression. We focused on genes whose expression could be detected in at least 40% of cells because we wanted the feature to be detectable in a substantial fraction of cells in order to be used by the classifiers (**Fig. EV9B**). Next, we reasoned that genes whose expression was clonally correlated could carry the most information that a classifier could learn to assign clones to clonal families. We showed that random sets of genes whose clonal correlation was lower than 0.25 were not informative enough and all classifiers perform poorly when using these sets of genes (average of 100 random sets of genes with r <0.25; LR accuracy 0.276 ± 0.031; NN accuracy 0.274 ± 0.030, RF accuracy 0.246 ± 0.029) (**Fig. R15B** and **Fig. EV10B**). On the contrary, when genes whose clonal correlation coefficient above 0.25 were used as input features, all three classifiers correctly assigned cells to the correct clones with above 80% of accuracy (LR accuracy 0.876 ± 0.012; NN accuracy 0.828 ± 0.019, RF accuracy 0.722 ± 0.019) (**Fig. R15B** and **Fig. EV10B**). Overall, logistic regression performed the best in both classification accuracy (0.876 ± 0.012) and calibration (Brier score loss=0.207 ± 0.015) followed by the neural network model (accuracy= 0.828 ± 0.019, Brier score loss=0.253 ± 0.026) (**Fig. R15B** and **Fig. EV10B**). The random forest classifier had the lowest accuracy (0.722 ± 0.019) and the model was the less calibrated one as shown by the higher Brier score loss (0.727 ± 0.009).

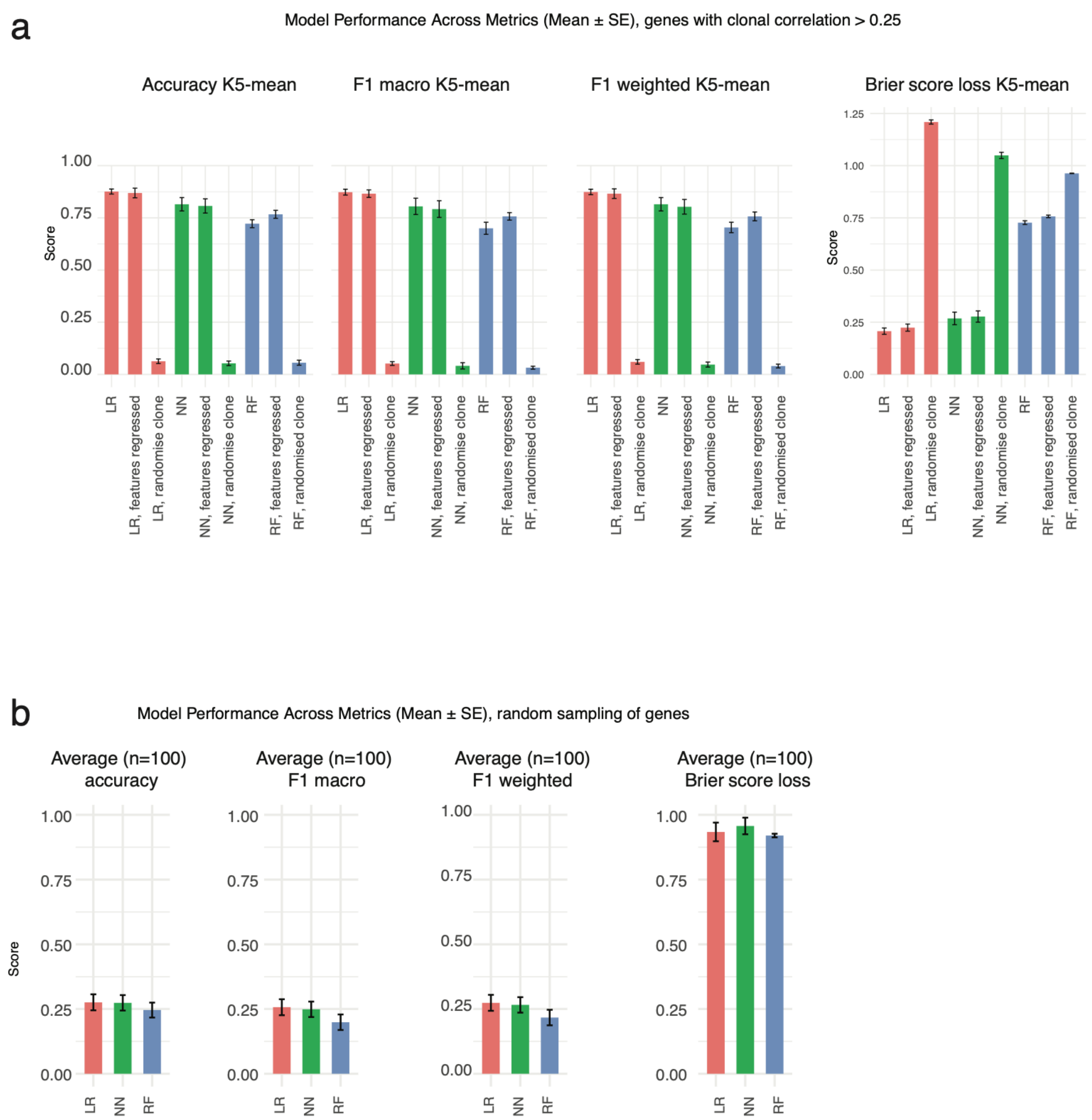

Figure R15. **a**) Bar plot showing the performance (accuracy, F1-score, Brier score loss) of Logistic regression (LR), Neural Network (NN) and Random Forest (RF) models used to predict clonal identity based on gene expression profiles. Each bar represents the mean and standard error of the performance metric across cross-validation folds (K=5). Models compared include logistic regression, random forest and neural network. All of the models used as input the same gene expression profiles from two day 6 post stimulation naive clonal datasets generated for this paper from two human healthy blood donors. Genes with a clonal correlation higher that Pearson correlation coefficient 0.25 were used as input (369 genes). "Feature regressed" indicates cell cycle and cell state specific effects were regressed out from gene expression scaled values "randomised clone" indicates that the clonal assignments were shuffled per donor before training. **b**) Model performance of different machine learning classifiers as in **a**, however the input features were 369 genes randomly sampled from genes with clonal correlation lower than 0.25 ("random sampling of genes"). Average and standard error of 100 different sampling runs are shown.

Next, we randomly shuffled clonal assignments within each donor 1000 times to generate a null distribution of prediction accuracy, demonstrating that the classifiers did not learn any meaningful patterns in this scenario (**Fig. R16** and **Fig. EV11**).

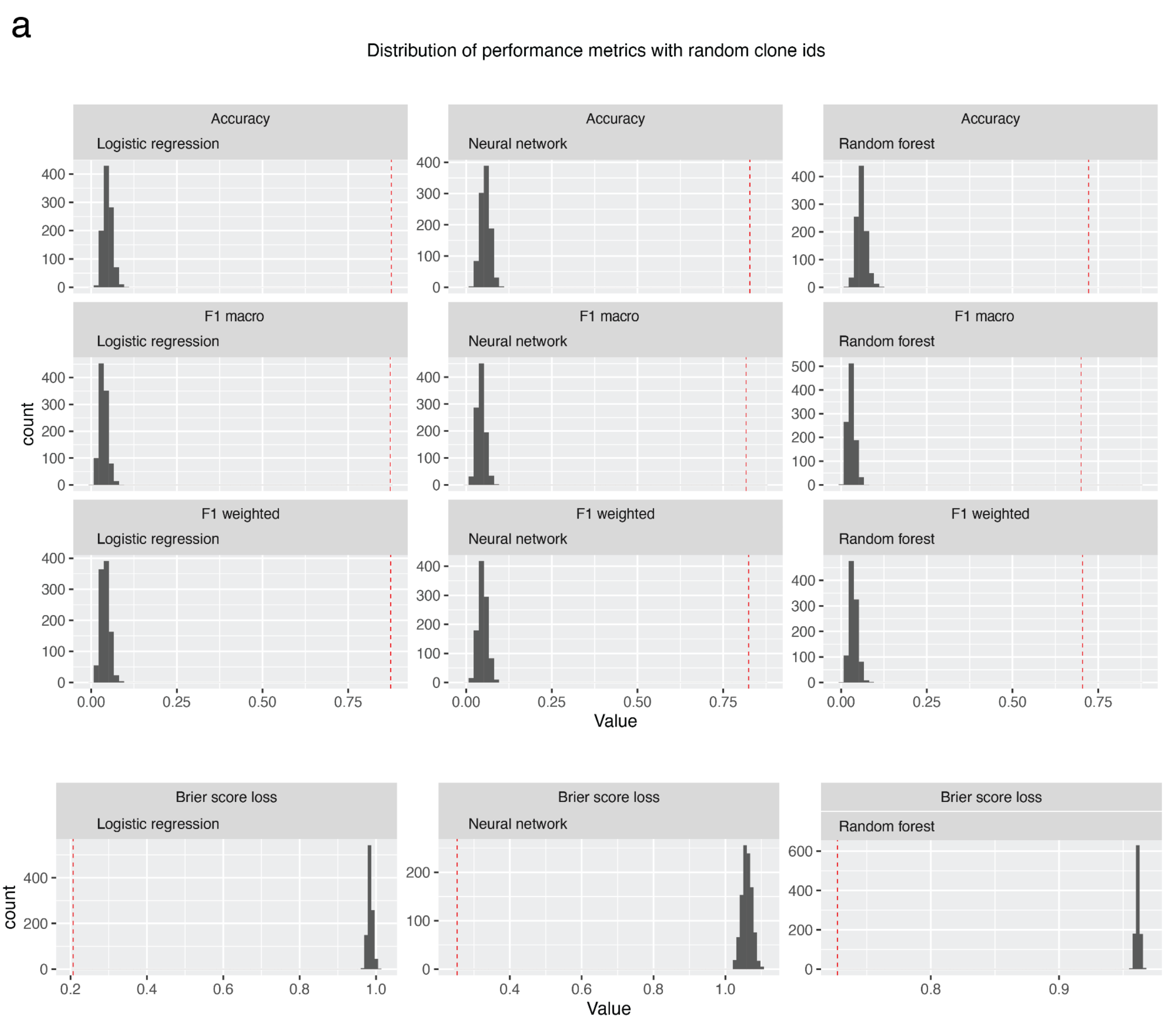

Figure R16. **a)** Distribution of accuracy metrics (accuracy, F1-score, Brier score loss) of various models used to predict clonal identity based on gene expression profiles, estimated 1000 times with randomised clone ids. Random distribution was estimated by shuffling the clone-ids per donor and computing the classification 1000 times. Red lines represent the mean accuracy metric obtained on the non-randomised data. Models compared include logistic regression, random forest and neural network. All of the models used as input the same gene expression profiles from two day 6 post stimulation naive clonal datasets generated for this paper from two human healthy blood donors. Genes with a clonal correlation higher that Pearson correlation coefficient 0.25 were used as input (369 genes).

Finally, to rule out that the models were learning cell cycle, plasma cell or germinal centre specific patterns, we regressed out cell cycle genes as well as XBP1, SPI1 and IRF4 scores from gene expression and performed classification with the regressed out features. Classification performances were unaltered (**Fig. R15B** and **Fig. EV10B**), indicating that the models were able to learn clonal specific signatures that are independent from cell cycle and cell state specific programs.

Moreover we conducted classification on 11 independent in vivo mice B cell clonal datasets, using the logistic regression (LR) and the neural network (NN) models described above. We did not apply random forest as LR and NN performed better in the classification task in our in house generated B clonal datasets. For 7 out of 11 mice datasets, classification accuracy was above 70% with both LR and NN, while for all samples accuracy was above 50% (**Fig. R17** and **Fig. EV12**) (NN accuracy across all mice 0.72 ± 0.098; LR accuracy across all mice 0.71 ± 0.10).

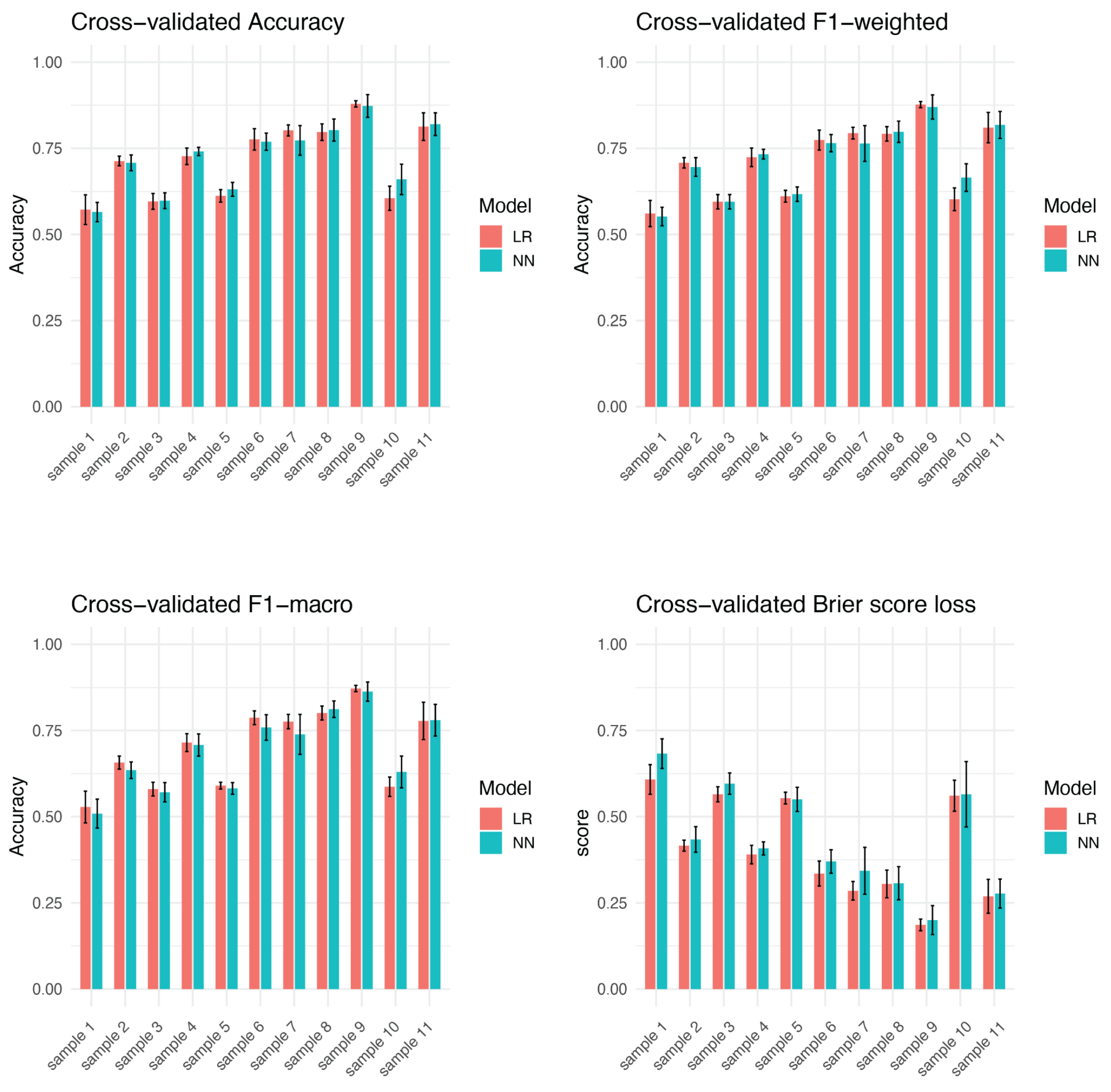

Figure R17. Bar plot showing the performance (accuracy, F1-score, Brier score loss) of various models used to predict clonal identity based on gene expression profiles on 11 mice B cell samples from Basso et al. Each bar represents the mean and standard error of the performance metric across cross-validation folds (K=5). Models compared include logistic regression (LR) and neural network (NN).

Using 100 random clonal assignments within each mice, the classifiers were not able to learn any patterns (**Fig. R18** and **Fig. EV13**), thus indicating the existence of clonal specific signatures that can be learned also in in vivo mice B cells.

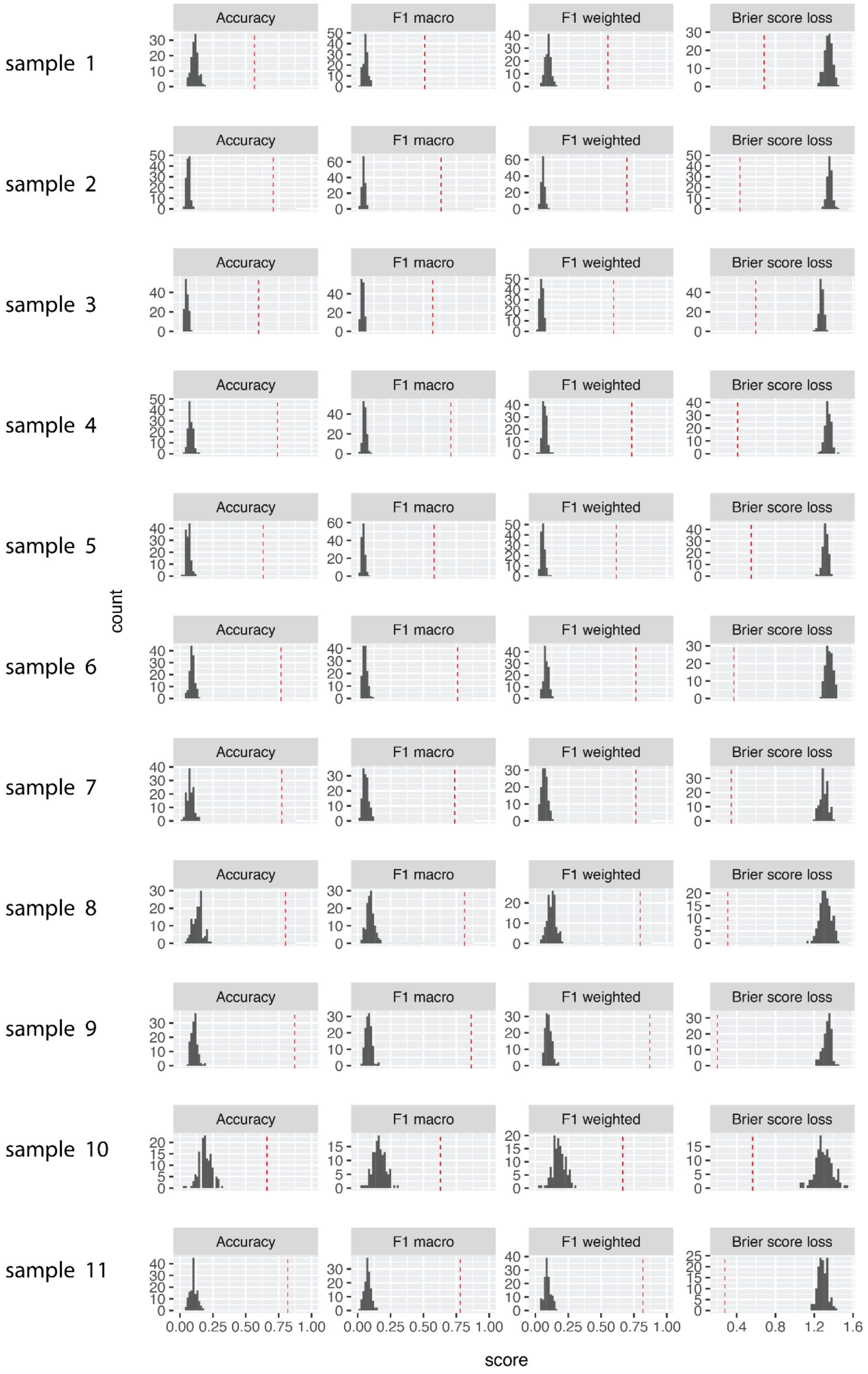

Figure R18. Distribution of accuracy metrics (accuracy, F1-score, Brier score loss) of the neural network used to predict clonal identity based on gene expression profiles, estimated 100 times with randomised clones within each mice B cell clonal dataset. Random distribution was estimated by shuffling the clone-ids within each mouse and computing the classification 100 times. Red lines represent the mean accuracy metric obtained on the non-randomised data.

The lower classification accuracy in in vivo mice samples could be explained with the fact that in the vivo setting, cells from the same clones might have acquired different cell states in distant time frames. Therefore, transcriptomes from different cells in the same clones might have diverged into different cell states thus making the classification harder. Nevertheless, the classifiers are still able to capture a clonal specific signature in in vivo mice B cells, validating our in vitro clonal experimental system.

We detailed the architectures and parameters in the Methods section and expanded the supplementary figures and tables.

Minor concern

1. Missing methodological specifics to ensure reproducibility
a. SCENIC - Add SEAcells parameters (number cells per metacell, k, seed), motif database versions, GRN stability thresholds (80% regulon retention; 70% gene retention) and rationale; exact AUCell settings and RSS calculation.
b. sciCSR - It is cited in Results but absent in Methods. Please add version, reference, alignment/remapping parameters, GLT definitions, minimum read thresholds, and chemistry awareness (10x 5' v2).
c. QC transparency - Report numeric QC cutoffs (minimum genes/UMIs, mitochondrial %, doublet score thresholds) per time point and donor; give post-QC cell counts per group.

We thank the reviewer and agree that providing additional methodological details is key for reproducibility. We have substantially extended the methods sections in the main manuscript. Furthermore, we made available the github repository with all the code and all relevant python environments and additional software versions.

References

Agrafiotis, Andreas, Daniel Neumeier, Kai-Lin Hong, Tasnia Chowdhury, Roy Ehling, Raphael Kuhn, Ioana Sandu, et al. 2023. "Generation of a Single-Cell B Cell Atlas of Antibody Repertoires and Transcriptomes to Identify Signatures Associated with Antigen Specificity." *iScience* 26 (3): 106055.

Basso, Katia, Adam A. Margolin, Gustavo Stolovitzky, Ulf Klein, Riccardo Dalla-Favera, and Andrea Califano. 2005. "Reverse Engineering of Regulatory Networks in Human B Cells." *Nature Genetics* 37 (4): 382–90.

Ding, B. Belinda, Enguang Bi, Hongshan Chen, J. Jessica Yu, and B. Hilda Ye. 2013. "IL-21 and CD40L Synergistically Promote Plasma Cell Differentiation through Upregulation of Blimp-1 in Human B Cells." *The Journal of Immunology* 190 (4): 1827–36.

Ettinger, Rachel, Gary P. Sims, Anna-Marie Fairhurst, Rachel Robbins, Yong Sing da Silva, Rosanne Spolski, Warren J. Leonard, and Peter E. Lipsky. 2005. "IL-21 Induces Differentiation of Human Naive and Memory B Cells into Antibody-Secreting Plasma Cells." *Journal of Immunology* 175 (12): 7867–79.

Hipp, Nicolas, Hannah Symington, Cédric Pastoret, Gersende Caron, Céline Monvoisin, Karin Tarte, Thierry Fest, and Céline Delaloy. 2017. "IL-2 Imprints Human Naive B Cell Fate towards Plasma Cell through ERK/ELK1-Mediated BACH2 Repression." *Nature Communications* 8 (1): 1443.

Kamimoto, Kenji, Blerta Stringa, Christy M. Hoffmann, Kunal Jindal, Lilianna Solnica-Krezel, and Samantha A. Morris. 2023. "Dissecting Cell Identity via Network Inference and in Silico Gene Perturbation." *Nature*, February, 1–10.

Klein, Dominik, Giovanni Palla, Marius Lange, Michal Klein, Zoe Piran, Manuel Gander, Laetitia Meng-Papaxanthos, et al. 2025. "Mapping Cells through Time and Space with Moscot." *Nature* 638 (8052): 1065–75.

Kuchen, Stefan, Rachel Robbins, Gary P. Sims, Chen Sheng, Terence M. Phillips, Peter E. Lipsky, and Rachel Ettinger. 2007. "Essential Role of IL-21 in B Cell Activation, Expansion, and Plasma Cell Generation during CD4+ T Cell-B Cell Collaboration." *The Journal of Immunology* 179 (9): 5886–96.

La Manno, Gioele, Ruslan Soldatov, Amit Zeisel, Emelie Braun, Hannah Hochgerner, Viktor Petukhov, Katja Lidschreiber, et al. 2018. "RNA Velocity of Single Cells." *Nature* 560 (7719): 494–98.

Le Gallou, Simon, Gersende Caron, Céline Delaloy, Delphine Rossille, Karin Tarte, and Thierry Fest. 2012. "IL-2 Requirement for Human Plasma Cell Generation: Coupling Differentiation and Proliferation by Enhancing MAPK-ERK Signaling." *Journal of Immunology (Baltimore, Md. : 1950)* 189 (1): 161–73.

Marsman, Casper, Dorit Verhoeven, Jana Koers, Theo Rispens, Anja Ten Brinke, S. Marieke van Ham, and Taco W. Kuijpers. 2022. "Optimized Protocols for T-Cell-Dependent and T-Cell-Independent Activation for B-Cell Differentiation Studies Using Limited Cells." *Frontiers in Immunology* 13 (June):815449.

Pett, J. Patrick, Martin Prete, Duy Pham, Nick England, Hao Yuan, Elena Prigmore, Liz Tuck, et al. 2025. "An Atlas of TF Driven Gene Programs across Human Cells." *bioRxiv*. https://doi.org/10.1101/2025.05.30.657075.

Setty, Manu, Vaidotas Kiseliovas, Jacob Levine, Adam Gayoso, Linas Mazutis, and Dana Pe'er. 2019. "Characterization of Cell Fate Probabilities in Single-Cell Data with Palantir." *Nature Biotechnology* 37 (4): 451–60.

Wang, J., A. A. Lobito, F. Shen, F. Hornung, A. Winoto, and M. J. Lenardo. 2000. “Inhibition of Fas-Mediated Apoptosis by the B Cell Antigen Receptor through c-FLIP.” *European Journal of Immunology* 30 (1): 155–63.

Wu, Y., J. Xu, S. Shinde, I. Grewal, T. Henderson, R. A. Flavell, and Y. Liu. 1995. “Rapid Induction of a Novel Costimulatory Activity on B Cells by CD40 Ligand.” *Current Biology: CB* 5 (11): 1303–11.

Xu, Chenling, Romain Lopez, Edouard Mehlman, Jeffrey Regier, Michael I. Jordan, and Nir Yosef. 2021. “Probabilistic Harmonization and Annotation of Single-Cell Transcriptomics Data with Deep Generative Models.” *Molecular Systems Biology* 17 (1): e9620.

28th Jan 2026

Manuscript Number: MSB-2025-13265R
Title: Competing gene regulatory networks drive naive and memory B cell differentiation

Dear Dr Soskic,

Thank you for the submission of your revised manuscript to Molecular Systems Biology. We have now received the enclosed reports from the referees that were asked to re-assess it. As you will see the reviewers are now globally supportive and I am pleased to inform you that we will be able to accept your manuscript pending the following final amendments:

1) In the main manuscript file, please include keywords to max. 5.
2) Please ensure that the sequencing data listed in the Data availability section are now released and links updated to the publicly available site.
3) Please rename "Conflict of Interest" to "Disclosure and competing interests statement". We updated our journal's competing interests policy in January 2022 and request authors to consider both actual and perceived competing interests. Please review the policy https://link.springer.com/partners/embo-press/editorial-policies#Competing%20interest%20disclosures and update your competing interests if necessary.
4) We note that affiliation #1 is a company. Employment in a biotech company should be stated in the Disclosure and competing interests statement.
5) Please correct the reference citation such that when there there are more than 10 authors on a paper, only the first 10 should be listed, followed by "et al.". The format should be Last Name, First Name Initial. In addition, DOIs should only be used for preprints and datasets that have not been published yet.
Please check "Author Guidelines" for more information: https://link.springer.com/journal/44320/submission-guidelines#cms-Reference-guidelines
6) Our journal encourages inclusion of *data citations in the reference list* to directly cite datasets that were re-used and obtained from public databases. Data citations in the article text are distinct from normal bibliographical citations and should directly link to the database records from which the data can be accessed. In the main text, data citations are formatted as follows: "Data ref: Smith et al, 2001" or "Data ref: NCBI Sequence Read Archive PRJNA342805, 2017". In the Reference list, data citations must be labeled with "[DATASET]". A data reference must provide the database name, accession number/identifiers and a resolvable link to the landing page from which the data can be accessed at the end of the reference. Further instructions are available at .
7) In the Methods, please take care of the following:
- Please state, in the section where you declare that the study was authorized for human participants, that the experiments conformed to the principles set out in the WMA Declaration of Helsinki and the Department of Health and Human Services Belmont Report. Please note that this is a separate statement from the specific ethics committee approval and informed consent.
8) Please place individual sections of the manuscript in the following order: Title page - Abstract & Keywords - Introduction - Results - Discussion - Methods - Data Availability - Acknowledgements - Disclosure and Competing Interests Statement - References - Figure Legends - Expanded View Figure Legends.
9) For the figures and figure legends, please take care of the following:
- 18 Expanded View figures is far more than we usually allow. We would suggest moving some of these figures into the main figures or into an Appendix file. In the Appendix file, please ensure the word "Appendix" is included in all labels for Appendix Figures/Tables including in the Table of Contents with the nomenclature as "Appendix Figure S1". The title page should have "Appendix for [manuscript title]" followed by a Table of Contents with page numbers. The legends in the Appendix should be placed under each respective figure. Please upload the Appendix as a single PDF (no separate image files are needed). Please ensure that the callouts are also updated in the main manuscript.
- Please note that the exact p values are not provided in the legend of figure 3I
- Please indicate the statistical test used for data analysis in the legend of figure EV14 C
- Please note that the box plots need to be defined in terms of minima, maxima, centre, bounds of box and whiskers, and percentile in the legends of figures 2D, 4A, C; 5B, EV1 B, C; EV9 A
- Please note that information related to n is missing in the legends of figures 1D, 2D, 3F, G; 4A-D, H, I; 5B, EV4 B
10) Please upload all supplementary table/excel files individually (not zipped together) and rename them as Dataset EV1-EV10. Each legend should be provided in each file as a separate sheet/tab, not as a separate text file. The nomenclature needs correction in all places: source file names, legends, titles in the system, as well as callouts in the manuscript.
11) Please ensure that all funding sources are entered into the manuscript submission system separately (Comments box should not be used for this).
12) Synopsis:
- Synopsis image: Please provide a graphic that summarises the main findings of the manuscript on a glance and upload it as a high-resolution jpeg file 550 pixels wide x (300-600) pixels high.
- Synopsis text: Please provide a separate word document including a short standfirst (maximum of 300 characters, including

spaces) and up to 5 bullet points to summarise the key NEW findings. They should be designed to be complementary to the abstract - i.e. not repeat the same text. We encourage inclusion of key acronyms and quantitative information (maximum of 30 words / bullet point). Please use the passive voice.
- Please check your synopsis text and image before submission with your revised manuscript. Please be aware that in the proof stage minor corrections only are allowed (e.g., typos).
13) Source Data: Please organize the Source Data as a single source data file (zipped) per figure for main figures (all EV and/or Appendix figure Source Data can be included in a single folder), with the panels clearly visible in the folder structure instead of a single excel file for all Source Data. e.g. all the Source data files for figure 1 need to be saved in a single folder and this needs to be zipped and then uploaded as "SD figure 1.zip" file.
14) Please upload the raw, unedited image Source Data for Figures 2 and EV8 B & C.
15) Please provide the .fcs Source Data in a format readable by standard softwares.
16) As part of the EMBO Publications transparent editorial process initiative (see our policy here: https://www.embopress.org/transparent-process#Review_Process), Molecular Systems Biology will publish online a Peer Review File (PRF) to accompany accepted manuscripts. This file will be published in conjunction with your paper and will include the anonymous referee reports, your point-by-point response and all pertinent correspondence relating to the manuscript. Let us know whether you agree with the publication of the PRF and as here, if you want to remove or not any figures from it prior to publication. Please note that the Authors checklist will be published at the end of the PRF.
17) After your paper is published, we may promote it on social media. If you have any handles or hashtags for Bluesky you would like included, please let us know.
18) Please provide a point-by-point letter INCLUDING my comments and your detailed responses (as Word file).

I look forward to reading a new revised version of your manuscript as soon as possible.

Yours sincerely,

Poonam Bheda, PhD
Scientific Editor
Molecular Systems Biology

Reviewer #1:

The authors have fully addressed the reviewer's concers.

Reviewer #2:

In this revised edition, the authors have addressed my concerns, and I have no further critical comments.

Reviewer #3:

The authors have thoroughly revised the manuscript and addressed all my previous concerns. They now provide the missing dynamic and benchmarking evidence required to support their central claims. Specifically, the inclusion of Mascot analyses across the naïve and memory B-cell compartments substantially strengthens the bifurcation narrative. The mechanistic framework is further supported by orthogonal TF-activity validation utilizing VIPER with curated B-cell regulons and confirming IRF4-SPI1 anticorrelation. Furthermore, CellOracle in silico perturbations provided causal support that aligns with known knockout phenotypes, such as IRF4/PRDM1 loss suppressing plasma cell differentiation. Finally, the authors performed a more rigorous benchmarking of the clonal-identity predictor against LR/RF baselines. Altogether, the revised manuscript is significantly more robust in its treatment of dynamics, regulation, causality, and model justification.

1) In the main manuscript file, please include keywords to max. 5.
Keywords have now been added to the manuscript: B cell differentiation, gene regulatory networks, plasma cells, IRF4.

2) Please ensure that the sequencing data listed in the Data availability section are now released and links updated to the publicly available site.
All sequencing data listed in the Data Availability section have now been fully released. The raw single-cell gene expression and BCR-seq data have been released via the European Genome-phenome Archive (EGA) under accession numbers EGAD50000002113, EGAD50000002114, and EGAD50000002115. In addition, the processed single-cell data (h5ad files) are publicly available on Zenodo https://doi.org/10.5281/zenodo.17984776 . All scripts required to reproduce the data processing and figures have been made available at https://github.com/SoskicLab/bcelltimecourse . The Data Availability section has been updated accordingly with the relevant links.

3) Please rename "Conflict of Interest" to "Disclosure and competing interests statement". We updated our journal's competing interests policy in January 2022 and request authors to consider both actual and perceived competing interests. Please review the policy https://link.springer.com/partners/embo-press/editorial-policies#Competing%20interest%20disclosures and update your competing interests if necessary.
The section has been renamed. We have also reviewed the updated competing interests policy.

4) We note that affiliation #1 is a company. Employment in a biotech company should be stated in the Disclosure and competing interests statement.
Thank you for the comment. We would like to clarify that Human Technopole is not a biotech company. It is a publicly funded research institute established by the Italian government, operates on a not-for-profit basis, and does not engage in commercial biotech activities.

5) Please correct the reference citation such that when there there are more than 10 authors on a paper, only the first 10 should be listed, followed by "et al.". The format should be Last Name, First Name Initial. In addition, DOIs should only be used for preprints and datasets that have not been published yet.
Please check "Author Guidelines" for more information: https://link.springer.com/journal/44320/submission-guidelines#cms-Reference-guidelines
The reference list has been revised to conform to the journal’s guidelines.

6) Our journal encourages inclusion of *data citations in the reference list* to directly cite datasets that were re-used and obtained from public databases. Data citations in the article text are distinct from normal bibliographical citations and should directly link to the database records from which the data can be accessed. In the main text, data citations are formatted as follows: "Data ref: Smith et al, 2001" or "Data ref: NCBI Sequence Read Archive PRJNA342805, 2017". In the Reference list, data citations must be labeled with "[DATASET]". A data reference must provide the database name, accession number/identifiers and a resolvable link to the landing page from which the data can be accessed at the end of the reference. Further instructions are available at https://www.embopress.org/page/journal/17574684/authorguide#referencesformat.
We have added data citations in the main text and included the corresponding entries in the Reference list, clearly labeled with **[DATASET].**

7) In the Methods, please take care of the following:
- Please state, in the section where you declare that the study was authorized for human participants, that the experiments conformed to the principles set out in the WMA Declaration of Helsinki and the Department of Health and Human Services Belmont Report. Please note that this is a separate statement from the specific ethics committee approval and informed consent.
We have added a statement.

8) Please place individual sections of the manuscript in the following order: Title page - Abstract & Keywords - Introduction - Results - Discussion - Methods - Data Availability - Acknowledgements - Disclosure and Competing Interests Statement - References - Figure Legends - Expanded View Figure Legends.
We placed the sections in the correct order.

9) For the figures and figure legends, please take care of the following:
- 18 Expanded View figures is far more than we usually allow. We would suggest moving some of these figures into the main figures or into an Appendix file. In the Appendix file, please ensure the word "Appendix" is included in all labels for Appendix Figures/Tables including in the Table of Contents with the nomenclature as "Appendix Figure S1". The title page should have "Appendix for [manuscript title]" followed by a Table of Contents with page numbers. The legends in the Appendix should be placed under each respective figure. Please upload the Appendix as a single PDF (no separate image files are needed). Please ensure that the callouts are also updated in the main manuscript.
All Expanded View figures have been converted into an Appendix.

- Please note that the exact p values are not provided in the legend of figure 3I

Exact p-values for Fig. 3I could not be calculated because they fall below the numerical precision limits of the computational tools used. Rather than reporting these values as *p* = 0, as it is sometimes done, we have retained the current notation to accurately reflect this limitation.

- Please indicate the statistical test used for data analysis in the legend of figure EV14 C

Enrichment significance was assessed using a hypergeometric test.

- Please note that the box plots need to be defined in terms of minima, maxima, centre, bounds of box and whiskers, and percentile in the legends of figures 2D, 4A, C; 5B, EV1 B, C; EV9 A

We defined boxplots as indicated.

- Please note that information related to n is missing in the legends of figures 1D, 2D, 3F, G; 4A-D, H, I; 5B, EV4 B

We defined n as indicated.

10) Please upload all supplementary table/excel files individually (not zipped together) and rename them as Dataset EV1-EV10. Each legend should be provided in each file as a separate sheet/tab, not as a separate text file. The nomenclature needs correction in all places: source file names, legends, titles in the system, as well as callouts in the manuscript.

We renamed Dataset as indicated.

11) Please ensure that all funding sources are entered into the manuscript submission system separately (Comments box should not be used for this).

We have declared funding sources.

12) Synopsis:

- Synopsis image: Please provide a graphic that summarises the main findings of the manuscript on a glance and upload it as a high-resolution jpeg file 550 pixels wide x (300-600) pixels high.

We have provided a figure that summarises the main findings.

- Synopsis text: Please provide a separate word document including a short standfirst (maximum of 300 characters, including spaces) and up to 5 bullet points to summarise the key NEW findings. They should be designed to be complementary to the abstract - i.e. not repeat the same text. We encourage inclusion of key acronyms and quantitative information (maximum of 30 words / bullet point). Please use the passive voice.
- Please check your synopsis text and image before submission with your revised

manuscript. Please be aware that in the proof stage minor corrections only are allowed (e.g., typos).
We provided a document containing the synopsis text. This includes a standfirst and five bullet points summarizing the key findings.

13) Source Data: Please organize the Source Data as a single source data file (zipped) per figure for main figures (all EV and/or Appendix figure Source Data can be included in a single folder), with the panels clearly visible in the folder structure instead of a single excel file for all Source Data. e.g. all the Source data files for figure 1 need to be saved in a single folder and this needs to be zipped and then uploaded as "SD figure 1.zip" file.
We have provided the source data as indicated.

14) Please upload the raw, unedited image Source Data for Figures 2 and EV8 B & C.
We have provided the raw data as indicated.

15) Please provide the .fcs Source Data in a format readable by standard softwares.
The flow cytometry source data have been provided as standard files (Flow Cytometry Standard file format, fcs) and we are not aware of any other format for this data. Fcs are compatible with all commonly used flow cytometry analysis software, including free ones or can be analyzed using open source R and Python scripts. The uploaded files have been verified to be readable in standard programs.

16) As part of the EMBO Publications transparent editorial process initiative (see our policy here: https://www.embopress.org/transparent-process#Review_Process), Molecular Systems Biology will publish online a Peer Review File (PRF) to accompany accepted manuscripts. This file will be published in conjunction with your paper and will include the anonymous referee reports, your point-by-point response and all pertinent correspondence relating to the manuscript. Let us know whether you agree with the publication of the PRF and as here, if you want to remove or not any figures from it prior to publication. Please note that the Authors checklist will be published at the end of the PRF.
We agree with the publication of PRF.

17) After your paper is published, we may promote it on social media. If you have any handles or hashtags for Bluesky you would like included, please let us know.
We do not have Bluesky account.

18) Please provide a point-by-point letter INCLUDING my comments and your detailed responses (as Word file).

We have responded to all the comments.

Reviewer comments

Reviewer #1:

The authors have fully addressed the reviewer's concers.
We thank the reviewer for their positive assessment and are pleased that all concerns have been fully addressed.

Reviewer #2:

In this revised edition, the authors have addressed my concerns, and I have no further critical comments.
We thank the reviewer for their evaluation and are pleased to hear that all concerns have been addressed.

Reviewer #3:

The authors have thoroughly revised the manuscript and addressed all my previous concerns. They now provide the missing dynamic and benchmarking evidence required to support their central claims. Specifically, the inclusion of Mascot analyses across the naïve and memory B-cell compartments substantially strengthens the bifurcation narrative. The mechanistic framework is further supported by orthogonal TF-activity validation utilizing VIPER with curated B-cell regulons and confirming IRF4-SPI1 anticorrelation. Furthermore, CellOracle in silico perturbations provided causal support that aligns with known knockout phenotypes, such as IRF4/PRDM1 loss suppressing plasma cell differentiation. Finally, the authors performed a more rigorous benchmarking of the clonal-identity predictor against LR/RF baselines. Altogether, the revised manuscript is significantly more robust in its treatment of dynamics, regulation, causality, and model justification.
We thank the reviewer for their positive assessment. We are grateful for their recognition that the additional analyses strengthened the central conclusions of our study.

23rd Mar 2026
Manuscript number: MSB-2025-13265RR
Title: Competing gene regulatory networks drive naive and memory B cell differentiation

Dear Dr Soskic,

Congratulations on an excellent manuscript, I am pleased to inform you that your manuscript has been accepted for publication in Molecular Systems Biology. Thank you for your comprehensive response to referee concerns. It has been a pleasure to work with you to get this to the acceptance stage.

Your manuscript will be processed for publication by EMBO Press. It will be copy edited and you will receive page proofs prior to publication. Please note that you will be contacted by Springer Nature Author Services to complete licensing and payment information.

You may qualify for financial assistance for your publication charges - either via a Springer Nature fully open access agreement or an EMBO initiative. Check your eligibility: https://link.springer.com/journal/44320/how-to-publish-with-us

Should you be planning a Press Release on your article, please get in contact with embo_production@springernature.com as early as possible in order to coordinate publication and release dates.

If you have any questions, please do not hesitate to contact the Editorial Office. Thank you for your contribution to Molecular Systems Biology.

Yours sincerely,

Poonam Bheda, PhD
Scientific Editor
Molecular Systems Biology

>>> Please note that it is Molecular Systems Biology policy for the transcript of the editorial process (containing referee reports and your response letter) to be published as an online supplement to each paper. If you do NOT want this, you will need to inform the Editorial Office via email immediately. More information is available here: https://link.springer.com/partners/embo-press/editorial-policies#Peer%20review

EMBO Press Author Checklist

Corresponding Author Name: Blagoje Soskic	
Journal Submitted to: Molecular Systems Biology	
Manuscript Number: MSB-2025-13265R	

USEFUL LINKS FOR COMPLETING THIS FORM
The EMBO Journal - Author Guidelines
EMBO Reports - Author Guidelines
Molecular Systems Biology - Author Guidelines
EMBO Molecular Medicine - Author Guidelines

Reporting Checklist for Life Science Articles (updated January
This checklist is adapted from Materials Design Analysis Reporting (MDAR) Checklist for Authors. MDAR establishes a minimum set of requirements in transparent reporting in the life sciences (see Statement of Task: 10.31222/osf.io/9sm4x). Please follow the journal's guidelines in preparing your manuscript.
Please note that a copy of this checklist will be published alongside your article.

Abridged guidelines for figures
1. Data
The data shown in figures should satisfy the following conditions:
- the data were obtained and processed according to the field's best practice and are presented to reflect the results of the experiments in an accurate and unbiased manner.
- ideally, figure panels should include only measurements that are directly comparable to each other and obtained with the same assay.
- plots include clearly labeled error bars for independent experiments and sample sizes. Unless justified, error bars should not be shown for technical
- if n<5, the individual data points from each experiment should be plotted. Any statistical test employed should be justified.
- Source Data should be included to report the data underlying figures according to the guidelines set out in the authorship guidelines on Data

2. Captions
Each figure caption should contain the following information, for each panel where they are relevant:
- a specification of the experimental system investigated (eg cell line, species name).
- the assay(s) and method(s) used to carry out the reported observations and measurements.
- an explicit mention of the biological and chemical entity(ies) that are being measured.
- an explicit mention of the biological and chemical entity(ies) that are altered/varied/perturbed in a controlled manner.
- the exact sample size (n) for each experimental group/condition, given as a number, not a range;
- a description of the sample collection allowing the reader to understand whether the samples represent technical or biological replicates (including how many animals, litters, cultures, etc.).
- a statement of how many times the experiment shown was independently replicated in the laboratory.
- definitions of statistical methods and measures:
  - common tests, such as t-test (please specify whether paired vs. unpaired), simple χ2 tests, Wilcoxon and Mann-Whitney tests, can be unambiguously identified by name only, but more complex techniques should be described in the methods section;
  - are tests one-sided or two-sided?
  - are there adjustments for multiple comparisons?
  - exact statistical test results, e.g., P values = x but not P values < x;
  - definition of 'center values' as median or average;
  - definition of error bars as s.d. or s.e.m.

Please complete ALL of the questions below.
Select "Not Applicable" only when the requested information is not relevant for your study.

Materials

Newly Created Materials	Information included in the manuscript?	In which section is the information available? (Reagents and Tools Table, Materials and Methods, Figures, Data Availability Section)
New materials and reagents need to be available; do any restrictions apply?	Not Applicable	

Antibodies	Information included in the manuscript?	In which section is the information available? (Reagents and Tools Table, Materials and Methods, Figures, Data Availability Section)
For **antibodies** provide the following information:<br>- Commercial antibodies: RRID (if possible) or supplier name, catalogue number and or/clone number<br>- Non-commercial: RRID or citation	Yes	Reagent Table

DNA and RNA sequences	Information included in the manuscript?	In which section is the information available? (Reagents and Tools Table, Materials and Methods, Figures, Data Availability Section)
**Short novel DNA or RNA including primers, probes**: provide the sequences.	Yes	Reagent Table, Materials and Methods

Cell materials	Information included in the manuscript?	In which section is the information available? (Reagents and Tools Table, Materials and Methods, Figures, Data Availability Section)
**Cell lines:** Provide species information, strain. Provide accession number in repository **OR** supplier name, catalog number, clone number, and/**OR** RRID.	Not Applicable	
**Primary cultures:** Provide species, strain, sex of origin, genetic modification status.	Yes	Materials and Methods, Results, Sex is not available as an information provided by the hospital
Report if the cell lines were recently **authenticated** (e.g., by STR profiling) and tested for mycoplasma contamination.	Not Applicable	

Experimental animals	Information included in the manuscript?	In which section is the information available? (Reagents and Tools Table, Materials and Methods, Figures, Data Availability Section)
**Laboratory animals or Model organisms:** Provide species, strain, sex, age, genetic modification status. Provide accession number in repository **OR** supplier name, catalog number, clone number, **OR** RRID.	Not Applicable	
**Animal observed in or captured from the field:** Provide species, sex, and age where possible**.**	Not Applicable	
Please detail **housing and husbandry conditions**.	Not Applicable	

Plants and microbes	Information included in the manuscript?	In which section is the information available? (Reagents and Tools Table, Materials and Methods, Figures, Data Availability Section)
**Plants:** provide species and strain, ecotype and cultivar where relevant, unique accession number if available, and source (including location for collected wild specimens).	Not Applicable	
**Microbes:** provide species and strain, unique accession number if available, and source.	Not Applicable	

Human research participants	Information included in the manuscript?	In which section is the information available? (Reagents and Tools Table, Materials and Methods, Figures, Data Availability Section)
If collected and within the bounds of privacy constraints report on age, sex and gender or ethnicity for all study participants.	Yes	Materials and Methods, information of age, sex and gender identity are not provided by the hospital from which we got the samples

Core facilities	Information included in the manuscript?	In which section is the information available? (Reagents and Tools Table, Materials and Methods, Figures, Data Availability Section)
If your work benefited from core facilities, was their service mentioned in the acknowledgments section?	Yes	Acknowledgments section

Design

Study protocol	Information included in the manuscript?	In which section is the information available? (Reagents and Tools Table, Materials and Methods, Figures, Data Availability Section)
If study protocol has been **pre-registered, provide DOI in the manuscript**. For clinical trials, provide the trial registration number **OR** cite DOI.	Not Applicable	
Report the **clinical trial registration number** (at ClinicalTrials.gov or equivalent), where applicable.	Not Applicable	

Laboratory protocol	Information included in the manuscript?	In which section is the information available? (Reagents and Tools Table, Materials and Methods, Figures, Data Availability Section)
Provide DOI OR other citation details if **external detailed step-by-step protocols** are available.	Not Applicable	

Experimental study design and statistics	Information included in the manuscript?	In which section is the information available? (Reagents and Tools Table, Materials and Methods, Figures, Data Availability Section)
Include a statement about **sample size** estimate even if no statistical methods were used.	Yes	Results, Material and Methods
Were any steps taken to minimize the effects of subjective bias when allocating animals/samples to treatment (e.g. **randomization procedure**)? If yes, have they been described?	Yes	Results, Material and Methods
Include a statement about **blinding** even if no blinding was done.	Yes	Material and Methods
Describe **inclusion/exclusion criteria** if samples or animals were excluded from the analysis. Were the criteria pre-established?<br><br>If sample or data points were omitted from analysis, report if this was due to attrition or intentional exclusion and provide justification.	Yes	Results, Material and Methods
For every figure, are **statistical tests** justified as appropriate? Do the data meet the assumptions of the tests (e.g., normal distribution)? Describe any methods used to assess it. Is there an estimate of variation within each group of data? Is the variance similar between the groups that are being statistically compared?	Yes	Results, Material and Methods

Sample definition and in-laboratory replication	Information included in the manuscript?	In which section is the information available? (Reagents and Tools Table, Materials and Methods, Figures, Data Availability Section)
In the figure legends: state number of times the experiment was **replicated** in laboratory.	Yes	Figures, Results, Materials and Methods
In the figure legends: define whether data describe **technical or biological replicates**.	Yes	Figures, Results, Materials and Methods

Ethics

Ethics	Information included in the manuscript?	In which section is the information available? (Reagents and Tools Table, Materials and Methods, Figures, Data Availability Section)
Studies involving **human participants**: State details of **authority granting ethics approval** (IRB or equivalent committee(s), provide reference number for approval.	Yes	Materials and Methods
Studies involving **human participants**: Include a statement confirming that **informed consent** was obtained from all subjects and that the experiments conformed to the principles set out in the WMA Declaration of Helsinki and the Department of Health and Human Services Belmont Report.	Yes	Materials and Methods
Studies involving **human participants:** For publication of **patient photos**, include a statement confirming that consent to publish was obtained.	Not Applicable	
Studies involving experimental **animals**: State details of **authority granting ethics approva**l (IRB or equivalent committee(s), provide reference number for approval. Include a statement of compliance with ethical regulations.	Not Applicable	
Studies involving **specimen and field samples:** State if relevant **permits** obtained, provide details of authority approving study; if none were required, explain why.	Not Applicable	

Dual Use Research of Concern (DURC)	Information included in the manuscript?	In which section is the information available? (Reagents and Tools Table, Materials and Methods, Figures, Data Availability Section)
Could your study fall under dual use research restrictions? Please check biosecurity documents and list of **select agents and toxins** (CDC): https://www.selectagents.gov/sat/list.htm	Not Applicable	
If you used a select agent, is the security level of the lab appropriate and reported in the manuscript?	Not Applicable	
If a study is subject to dual use research of concern regulations, is the name of the **authority granting approval and reference number** for the regulatory approval provided in the manuscript?	Not Applicable	

Reporting

The MDAR framework recommends adoption of discipline-specific guidelines, established and endorsed through community initiatives. Journals have their own policy about requiring specific guidelines and recommendations to complement MDAR.

Adherence to community standards	Information included in the manuscript?	In which section is the information available? (Reagents and Tools Table, Materials and Methods, Figures, Data Availability Section)
State if relevant guidelines or checklists (e.g., **ICMJE, MIBBI, ARRIVE, PRISMA**) have been followed or provided.	Not Applicable	
For **tumor marker prognostic studies,** we recommend that you follow the **REMARK** reporting guidelines (see link list at top right). See author guidelines, under 'Reporting Guidelines'. Please confirm you have followed these guidelines.	Not Applicable	
For **phase II and III randomized controlled trials**, please refer to the **CONSORT** flow diagram (see link list at top right) and submit the CONSORT checklist (see link list at top right) with your submission. See author guidelines, under 'Reporting Guidelines'. Please confirm you have submitted this list.	Not Applicable	

Data Availability

Data availability	Information included in the manuscript?	In which section is the information available? (Reagents and Tools Table, Materials and Methods, Figures, Data Availability Section)
Have **primary datasets** been deposited according to the journal's guidelines (see 'Data Deposition' section) and the respective accession numbers provided in the Data Availability Section?	Yes	Data availability
Were **human clinical and genomic datasets** deposited in a public access-controlled repository in accordance to ethical obligations to the patients and to the applicable consent agreement?	Yes	Data availability
Are **computational models** that are central and integral to a study available without restrictions in a machine-readable form? Were the relevant accession numbers or links provided?	Yes	Data availability
If publicly available data were reused, provide the respective **data citations in the reference list**.	Yes	Results, Citations
